# Optimizing properties of translocation-enhancing transmembrane proteins

**DOI:** 10.1016/j.bpj.2024.04.009

**Published:** 2024-04-13

**Authors:** Ladislav Bartoš, Martina Drabinová, Robert Vácha

**Affiliations:** 1 CEITEC – Central European Institute of Technology, Masaryk University, Brno, Czech Republic; 2 National Centre for Biomolecular Research, Faculty of Science, Masaryk University, Brno, Czech Republic; 3 Department of Condensed Matter Physics, Faculty of Science, Masaryk University, Brno, Czech Republic

## Abstract

Cell membranes act as semi-permeable barriers, often restricting the entry of large or hydrophilic molecules. Nonetheless, certain amphiphilic molecules, such as antimicrobial and cell-penetrating peptides, can cross these barriers. In this study, we demonstrate that specific properties of transmembrane proteins/peptides can enhance membrane permeation of amphiphilic peptides. Using coarse-grained molecular dynamics with free-energy calculations, we identify key translocation-enhancing attributes of transmembrane proteins/peptides: a continuous hydrophilic patch, charged residues preferably in the membrane center, and aromatic hydrophobic residues. By employing both coarse-grained and atomistic simulations, complemented by experimental validation, we show that these properties not only enhance peptide translocation but also speed up lipid flip-flop. The enhanced flip-flop reinforces the idea that proteins such as scramblases and insertases not only share structural features but also operate through identical biophysical mechanisms enhancing the insertion and translocation of amphiphilic molecules. Our insights offer guidelines for the designing of translocation-enhancing proteins/peptides that could be used in medical and biotechnological applications.

## Significance

Cells are enveloped by a selectively permeable cytoplasmic membrane. Certain peptides can spontaneously penetrate this protective barrier, serving as potential drug carriers or therapeutic agents. In this study, we demonstrate that the translocation of peptides across membrane into cell can be enhanced with the passive assistance of transmembrane proteins. We investigate various properties of these transmembrane proteins that enhance the translocation and we establish guidelines for designing such translocation enhancers. We show that these enhancers also facilitate lipid scrambling, which suggests more general effect on amphiphilic molecules. Therefore, proteins facilitating the insertion of amphiphilic molecules into the membrane’s hydrophobic core, such as translocation enhancers, insertases, translocases, and scramblases, might share a common biophysical mechanism.

## Introduction

Living cells are enclosed by a thin phospholipid bilayer that controls the exchange of matter between the cell and its environment. Typically, only small and uncharged molecules can permeate the cell membrane directly, whereas the transport of other molecules requires specific, tightly regulated channels and transporters. Nonetheless, some larger amphiphilic molecules, including peptides, have been observed to spontaneously cross the cell membrane. Notably, antimicrobial peptides (AMPs) and cell-penetrating peptides (CPPs) stand out as potential therapeutic agents and drug carriers, respectively (1,2).

As research into the characteristics of AMPs and CPPs required for membrane translocation continues (3,4), it becomes clear that proteins within phospholipid/cellular membranes affect the local membrane properties, which could enable other molecules to enter the cell. Our prior study (5) demonstrated that transmembrane proteins or peptides (MPs) with a continuous hydrophilic region significantly enhance the translocation of amphiphilic peptides (TLPs) across the membrane. These MPs, with their hydrophilic patches, locally disrupt the membrane structure, allowing TLPs to more easily cross the membrane’s hydrophobic core. Furthermore, MPs provide enthalpic stabilization of the TLP via their hydrophilic residues. Interestingly, the presence of a hydrophilic patch spanning the membrane has also been observed in proteins known as scramblases, which facilitate lipid flip-flop, the translocation of phospholipids between the membrane’s leaflets (6,7,8,9,10).

**Figure 1:**
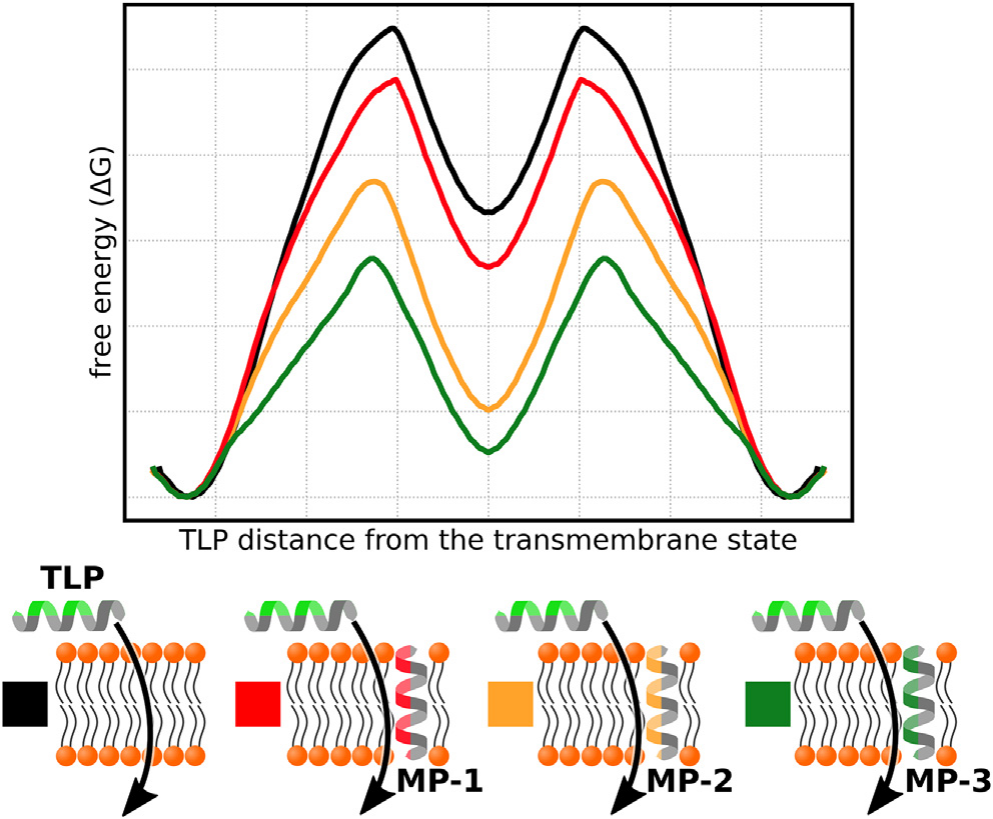
Aim of the study demonstrated on the translocation free-energy profiles, defined along the distance between the TLP and the membrane’s center of mass. MPs with different properties (illustrated by red, orange, and green patches) have different translocation-enhancing abilities. In this study, we searched for an optimal translocation enhancer: an MP that allows for the easiest passage of the TLP across the membrane. To see this figure in color, go online.

In this study, we used coarse-grained simulations to identify properties that maximize translocation enhancement beyond the size of the hydrophilic patch (see Fig. 1). Building on our earlier findings (5), we offer an extensive guide for designing proteins/peptides that enhance the translocation of *α*-helical amphiphilic peptides, such as uncharged AMPs or CPPs. Furthermore, through both coarse-grained and atomistic simulations as well as using experimental approaches, we demonstrate that the properties of MPs promoting peptide translocation can also accelerate lipid flip-flop.

## Materials and Methods

### Peptide translocation simulations

All of our simulations on peptide translocation were conducted using the Gromacs package versions 5.1.4 and 2021.4 (11). We utilized the coarse-grained force field, Martini version 2.2 (12,13,14). Lennard-Jones interactions between peptide and protein beads were downscaled by 10% to prevent overly strong protein-protein interactions (15). We refrained from using the Martini 3 force field because it fails to accurately represent a critical property for our simulations: the stability of transmembrane proteins in the membrane (16). Furthermore, this stability is closely tied to the membrane disruption caused by the MPs and probably arises from the excessively high hydrophilicities of the protein beads.

#### Systems preparation

Most systems consisted of a bacterial-membrane mimic composed of 256 lipids: 96 molecules of 1-palmitoyl-2-oleoyl-*sn*-glycero-3-phosphocholine (POPE) and 32 molecules of 1-palmitoyl-2-oleoyl-*sn*-glycero-3-phosphoglycerol (POPG) in each leaflet. We also prepared several additional systems with a membrane composed of 128 molecules of 1-palmitoyl-2-oleoyl-*sn*-glycero-3-phosphocholine (POPC) in each leaflet (256 lipids in total). Bilayers were was prepared using the CHARMM-GUI web interface (17). Each system had an approximate size of 9×9×11 nm and was solvated with roughly 4400 coarse-grained water beads.

Peptides and proteins were modeled using MODELLER versions 9.11 and 10.4 (18) and subsequently coarse-grained with the martinize.py script version 2.6 (https://github.com/cgmartini/martinize.py). We then minimized the potential energy of each peptide/protein in a vacuum environment using the steepest descent algorithm, setting a maximum force tolerance of 100 kJ mol^−1^ nm^−1^. All the peptides/proteins were generated with an *α*-helical conformation and were restrained to maintain this conformation throughout the simulation. We utilized the HeliQuest ComputParams.py script version 3 (https://heliquest.ipmc.cnrs.fr/cgi-bin/ComputParams.py) to calculate the hydrophobicity of each peptide/protein. The hydrophobicity scale is based on the partitioning of *N*-acetyl-amino-acid amides in octanol (19).

Translocating peptide (TLP) was subsequently placed on the membrane surface, oriented parallel to the membrane plane with its hydrophilic patch directed toward the solvent. The specific TLP sequence utilized was named LS9 (LSSLLSLLSSLLSLLSSLLSL-NH2) with a hydrophobicity value of 0.954. The transmembrane protein/peptide (MP) was centrally placed in the membrane, oriented perpendicular to the membrane plane. The sequences and hydrophobicities (H) of all MPs used can be found in Table 1. Note that the MPs SAGLE1, SAGLE^+^1, and SAGLE^0^1, have variations in the charge of their glutamate residue. Specifically, SAGLE1 possesses a negatively charged glutamate, SAGLE^+^1 has an artificial positively charged glutamate, and SAGLE^0^1 features a protonated (uncharged) glutamate. Additionally, we carried out simulations of TLP LS9 translocation without the presence of an MP as a control.

**Table 1:** Names, Sequences, and Hydrophobicities of MPs Used in the Study

Name	Sequence	H
SAGLS	SAGLSLLSLLLSLLLSLLSLGAS	0.902
SAGLT	SAGLTLLTLLLTLLLTLLTLGAS	0.967
SAGLQ	SAGLQLLQLLLQLLLQLLQLGAS	0.863
SAGLN	SAGLNLLNLLLNLLLNLLNLGAS	0.780
LNd3	NLLLNLLLLLLLLLLLLLLLLLL	1.500
LNd6	LLLNLLLNLLLLLLLLLLLLLLL	1.500
LNd9	LLLLLLNLLLNLLLLLLLLLLLL	1.500
LNd12	LLLLLLLLLNLLLNLLLLLLLLL	1.500
SAGLS9g0	SAGLSLLSSLLSLLSSLLSLGAS	0.750
SAGLS9g1	SAGSSLLLSLLSLLSLLLSSGAS	0.750
SAGLS9g2	SAGSSLLLLLSSSLLLLLSSGAS	0.750
SAGLS9g3	SAGLSSLLLLSSSLLLLSSLGAS	0.750
SAGLS9g4	SAGLSSSLLLLSLLLLSSSLGAS	0.750
SAGLS9g5	SAGLSLSLLSLSLSLLSLSLGAS	0.750
SAGLE1	SAGLLLLLLLLELLLLLLLLGAS	1.178
SAGLE^+^1	SAGLLLLLLLLELLLLLLLLGAS	1.178
SAGLE^0^1	SAGLLLLLLLLELLLLLLLLGAS	1.178
SAGLK1	SAGLLLLLLLLKLLLLLLLLGAS	1.163
SAGLS6E1	SAGLSLLSLLLELLLSLLSLGAS	0.876
F23	FFFFFFFFFFFFFFFFFFFFFFF	1.790
L23	LLLLLLLLLLLLLLLLLLLLLLL	1.700
SAGFS	SAGFSFFSFFFSFFFSFFSFGFS	1.013
SAGVS	SAGVSVVSVVVSVVVSVVSVGAS	0.651
SAGAS	SAGASAASAAASAAASAASAGAS	0.177
ENHTM1	NFFNNFFNNFFEFFNNFFNNFFN	0.645
ENHTM2	NGWFNFFNEFFEFFENFFNFWGN	0.734
ENHTM3	SLLSSLLSSLLKLLSSLLSSLLS	0.827
KLL	KKLLLLLLLLLLLLLLLLLLLLLLLKK	1.301
9Q3H	Ac-WKKLALALQLAHALALALALALKK-NH2	0.666

H, hydrophobicities.

After the addition of the TLP and MP, the system was ionized using Na^+^ and Cl^−^ ions at a physiological concentration of 154 mM, with an excess of ions to ensure system neutrality. Subsequently, each system underwent energy minimization employing the steepest descent algorithm, with a maximum force tolerance set at 100 kJ mol^−1^ nm^−1^.

#### Equilibration

Each system underwent equilibration in five stages with varying simulation time steps and overall durations: 1) dt =2 fs and t =0.5 ns, 2) dt =5 fs and t =1.25 ns, 3) dt =10 fs and t =1 ns, 4) dt =20 fs and t =30 ns, and 5) dt =20 fs and t =15 ns. Throughout stages 1–4, all backbone bead coordinates of both MP and TLP were restrained using a harmonic potential with a force constant of 1000 kJ mol^−1^ nm^−2^. In stage V of the equilibration and in subsequent molecular dynamics, pulling, and umbrella sampling simulations, a flat-bottomed potential ensured that the TLP remained in proximity to the MP. This potential was set between the centers of mass of the TLP and MP backbone beads with a reference distance of 2.5 nm in the xy plane. The applied force constant for this potential was 500 kJ mol^−1^ nm^−2^.

Each equilibration stage was performed in an NPT ensemble, maintaining a temperature of 310 K using the stochastic velocity rescaling thermostat (20) with a coupling constant set at 1 ps. The water and ions, as well as the membrane and peptides/proteins, were coupled separately. Pressure was kept at 1 bar using the Berendsen barostat (21) with a 12-ps coupling constant. A semi-isotropic pressure coupling scheme was used to scale the simulation box independently in the xy plane and along the *z* axis, with a compressibility of $3\times 10^{-4}$ bar^−1^. The Newtonian equations of motion were integrated using the leap-frog algorithm. Nonbonded interactions were cut off at 1.1 nm, and the van der Waals potential was shifted to zero at the cutoff distance. The relative dielectric constant was set at 15.

After the equilibration, a brief 100-ns molecular dynamics simulation was conducted. For this simulation, and all subsequent ones, the Berendsen thermostat was replaced with the Parrinello-Rahman barostat (22,23). All other simulation parameters were consistent with those from stage 5 of the equilibration.

#### Free-energy calculations

We enhanced the sampling of the configuration space using the umbrella sampling method (24,25). For the symmetric MPs (all MPs expect for LNd3, LNd6, and LNd9), we adopted the approach from our prior studies (5,26). Specifically, the TLP translocation process was split into two distinct steps: C terminus insertion and N terminus insertion. We excluded the TLP adsorption/desorption phase, as it previously remained unaffected by the presence of MPs (5). Given that both the membrane and the MP were symmetric, the free-energy profile for TLP’s translocation across the membrane was obtained by joining the free-energy profiles from each insertion process.

For the asymmetric MPs (LNd3, LNd6, LNd9), the translocation process was calculated separately for each possible “direction” of translocation. Given the symmetry of the membrane, we analyzed the insertion of each terminus of the TLP in two different orientations of the MP: once with the MP aligned at $180^{\circ }$ on the *z* axis and once at $0^{\circ }$ on the *z* axis within the membrane. This yielded four distinct insertion profiles labeled as N ↓, N ↑, C ↓, and C ↑. Here, the down arrow (↓) indicates the TLP’s insertion from the upper leaflet to the lower leaflet, and the up arrow (↑) signifies the reverse insertion direction. By pairing the relevant profiles (N ↓ with C ↑ and N ↑ with C ↓), we derived two full translocation profiles, each representing a specific translocation direction.

For each insertion process, we used a collective variable (CV) defined as the oriented distance between the terminus center of mass and the local membrane center of mass along the *z* axis. The terminus refers to either the first (N) or the last (C) three backbone beads of the TLP. The local membrane center of mass was determined using the lipid beads situated within a cylinder that had a radius of 2.0 nm, with its principal axis passing through the TLP terminus along the *z* axis.

In most of the simulated systems, the starting configurations for umbrella sampling simulations were obtained by pulling a single TLP terminus through the membrane. This terminus underwent pulling for 1 *μ*s at a rate of 4.2 nm *μ*s^−1^, starting from an initial reference distance of 2.1 nm. A harmonic potential, having a force constant of 8000 kJ mol^−1^ nm^−2^, was applied during this pulling simulation.

The trajectory from the pulling simulation was split into 64 nonuniformly spaced sampling windows, as detailed in Table S1 (for systems with POPE:POPG membrane) and Table S2 (for systems with POPC membrane). After a brief 30-ns equilibration, each window was subjected to sampling for at least 1 *μ*s.

For certain systems, specifically those with MPs F23, SAGFS, and SAGAS, sampling was further refined using Hamiltonian replica exchange (27) with an exchange attempt made every 5000 integration steps (100 ps) as implemented in the Plumed plugin version 2.3 (28). To ensure diverse system configurations, a “backward-pulling” simulation was performed for each TLP terminus. At the start of each backward pulling, the TLP was placed in a transmembrane state, and the respective terminus was pulled out of the membrane. Initial configurations for the umbrella sampling windows were taken from both forward- and backward-pulling simulations, with the origin of the windows alternating within the designated range of the CV.

The free-energy profile for each N or C terminus insertion was derived from the simulated umbrella sampling windows. This was done using the weighted histogram analysis method (29,30) as implemented in the tool g_wham (31).

#### Calculations of membrane disruption

To determine the impact of MP presence on the membrane’s structure, we simulated one additional system for each MP. In these simulations, a single MP was introduced into the membrane without any TLP. The system was prepared, minimized, and equilibrated following the above-mentioned procedures. The production molecular dynamics phase was 1 *μ*s, from which we computed 1) the local water defect (WD), i.e., the average count of water beads within 2.5 nm from the MP in the xy plane and within 2 nm from the membrane’s global geometric center on the *z* axis; and 2) the tail defect (TD), the average count of lipid tail beads within 2.0 nm from the MP in the xy plane and within 0.5 nm from the membrane’s geometric center on the *z* axis. Additionally, we characterized other defect-related metrics: 1) the upper leaflet water/TD, which exclusively counts water/tail beads positioned above the membrane’s center; and 2) the lower leaflet water/TD, which exclusively counts water/tail beads situated below the membrane’s center. The in-house-developed code used for the calculation of water and TD is available from https://github.com/Ladme/memdian.

### Lipid-scrambling simulations

All of our lipid-scrambling simulations were performed using Gromacs package versions 2021.4 and 2023-dev (11). Two force fields were employed for these simulations: 1) the coarse-grained Martini force field version 2.2 (12,13,14) and 2) the atomistic Amber 99SB-ILDN force field for describing the peptides/proteins combined with the atomistic Slipids 2020 force field for the lipids (32,33,34,35).

#### Martini simulations

Our Martini simulations of lipid scrambling followed a procedure akin to that of the peptide translocation simulations. We constructed a membrane comprising 288 POPC lipids (with 144 lipids in each leaflet) using the CHARMM-GUI web interface (17). This system spanned an approximate size of 10 × 10 × 12 nm and was solvated with roughly 6000 coarse-grained water beads. A transmembrane protein (MP), prepared as outlined in the peptide translocation simulations section, was centrally positioned within the membrane, oriented perpendicular to the membrane plane. Subsequent steps of minimization and equilibration were the same as for translocation simulations.

A lipid positioned close to the MP was selected and its phosphate bead was pulled through the membrane over a duration of 1 *μ*s. The pulling rate was set at 4.6 *μ*s^−1^ with an initial reference distance of 2.3 nm. The pulling was achieved using a harmonic potential with a force constant of 5000 kJ mol^−1^ nm^−2^. Our chosen CV was the oriented distance between the lipid’s phosphate and the local membrane’s center of mass along the *z* axis. This local membrane center of mass was calculated using the same criteria detailed in the peptide translocation simulations section. We restrained the pulled phosphate to the MP in the xy plane using a flat-bottomed potential, with a force constant of 500 kJ mol^−1^ nm^−2^ and a reference distance of 1.5 nm from the MP’s center of mass. In contrast to the peptide translocation simulations, the MP was fixed at the membrane’s center by employing a harmonic potential with a force constant of 100 kJ mol^−1^ nm^−2^. This maintained a reference distance of 0.0 nm from the local membrane center of mass along the *z* axis. The same protocol was used for MP free membrane, but without MP restraints.

Subsequently, the pulling trajectory was split into 67 nonuniform umbrella sampling windows (see Table S3). Each window was simulated for either 1 or 8 *μ*s (for POPC and POPC with ENHTM3, respectively). The initial 10 ns of these simulations were used for equilibration. The calculation of the free-energy profiles followed the procedure outlined in the “peptide translocation simulations” section, employing the weighted histogram analysis method (29,30).

#### Atomistic simulations

In the atomistic simulations, we utilized a pre-equilibrated POPC membrane obtained from the Slipids website (http://www.fos.su.se/~sasha/SLipids/Downloads_files/POPC_303K.gro). This starting membrane consisted of 128 POPC lipids (divided evenly with 64 lipids in each membrane leaflet) accompanied by approximately 5100 water molecules. We introduced NaCl ions into the system to achieve a concentration of 0.154 mol dm^−3^. After this, a small pore was created within the membrane during a 200-ps molecular dynamics simulation. This was accomplished with an inverted flat-bottomed potential, which had a force constant of 50 kJ mol^−1^ nm^−2^ and a reference distance of 1.1 nm from the box center. This potential was applied to the heavy atoms of all POPC lipids. After creating the pore, the MP (constructed using Avogadro version 1.2 (36)) was positioned within it. The membrane, with the embedded MP, was then energy minimized with a force tolerance of 1000 kJ mol^−1^ nm^−1^ and underwent equilibration. During this equilibration phase, the pore quickly sealed itself around the MP. For subsequent lipid-scrambling simulations where the MP was absent, we utilized a POPC membrane that did not have a pore at any point during the process.

The equilibration process for the membrane containing the MP spanned five distinct stages. In stage I, which was 2 ns long, position restraints, with a force constant of 1000 kJ mol^−1^ nm^−2^, were applied to all heavy protein atoms and to the phosphorus atoms of the lipids. During stage II, the position restraints previously applied to the lipids were removed, and the simulation ran for an additional 5 ns. In stage III, position restraints were placed only on the backbone atoms of the MP, which ran for 5 ns. In the subsequent stage IV, these restraints were confined only to the C_*α*_ atoms of the MP and the simulation ran for 5 ns. Finally, in stage V, no position restraints were enforced, and this final stage continued for 10 ns. All stages used a simulation time step of 2 fs. We employed the stochastic velocity rescaling thermostat (20) with a coupling constant of 0.5 ps to ensure a consistent temperature of 310 K. Separate thermal baths were designated for the water with ions and for the membrane with the MP. The pressure was set at 1 bar using the Berendsen barostat (21) with semi-isotropic pressure coupling, a coupling time of 2 ps, and a compressibility factor of 4.5 × 10^−5^ bar^−1^. The LINCS (37) method was used to constrain all bonds. Short-ranged, nonbonded interactions had a cutoff at 1.2 nm, whereas long-range electrostatic interactions employed the fast smooth particle-mesh Ewald method (38). The removal of translational velocity was performed separately for the membrane containing the MP and the water with ions.

After the equilibration, both the pure POPC and the POPC + MP membranes underwent 100 ns of molecular dynamics simulations with production parameters. At this stage, and in all subsequent simulations, we replaced the Berendsen barostat with the Parrinello-Rahman barostat (22,23). All other simulation settings remained consistent with those from stage V of equilibration. It is important to note that we did not artificially maintain the *α*-helical conformation of the MP.

Next, we selected a lipid located near the MP. We pulled this lipid from the upper leaflet to the lower leaflet by its phosphorus atom. This pulling lasted for 500 ns, with a pulling rate of 8.4 *μ*s^−1^ and an initial reference distance of 2.1 nm. We employed a harmonic potential with a force constant of 5000 kJ mol^−1^ nm^−2^. The CV mirrored the one used in the Martini 2 simulations of lipid scrambling; however, we used a phosphorus atom instead of a phosphate bead. In scenarios where an MP was present, we restrained the phosphorus to the MP in the xy plane using a flat-bottomed potential with a force constant of 500 kJ mol^−1^ nm^−2^ and a reference distance of 1.5 nm. For systems that included the MP, we also conducted pulling in the reverse direction—from the lower to the upper leaflet. In such cases, the initial reference distance was set to −2.1 nm, and the pulling direction was reversed, although all other simulation parameters remained unchanged.

The pulling trajectory obtained for the pure POPC system was then split into 59 nonuniformly distributed umbrella sampling windows (see Table S4 for details). To augment the sampling, we used Hamiltonian replica exchange (27) as implemented in the Plumed plugin version 2.7.2 (28) to 16 windows situated near the center of the membrane. Configuration exchanges were attempted every 100,000 integration steps (200 ps). Each window underwent simulation for 800 ns, with the initial 50 ns designated solely for equilibration. We calculated the free energy utilizing the weighted histogram analysis method (29,30).

For the POPC + ENHTM3 system, we divided the pulling trajectories into 99 nonuniformly distributed umbrella sampling windows. The initial configurations for the 42 windows nearest the upper membrane leaflet were taken from the pulling where the lipid moved from the upper to the lower leaflet. Conversely, the initial configurations for the 41 windows closest to the lower membrane leaflet were taken from the pulling in the reverse direction. For the 16 windows located near the membrane’s center, the configurations’ origin alternated. Refer to Table S5 for further details. Sampling within these central windows was enhanced using the Hamiltonian replica exchange (27). Configuration exchanges in these windows were attempted every 100,000 integration steps (200 ps). Every window underwent simulation for 600 ns, with the initial 200 ns designated solely for equilibration. The free energy was calculated from the entire set of umbrella sampling windows using the weighted histogram analysis method (29,30).

### Lipid-scrambling experiments

#### Large unilamellar vesicles

POPC and POPG (13.6 *μ*L and 1.5 *μ*L of 25 mg/mL stock solutions in chloroform) (Avanti Lipids), together with 1-myristoyl-2-C6-NBD-PC (1.5 *μ*L of a 1 mg/mL stock solution in chloroform) (Avanti lipids) were added to a glass test tube. The solvent was evaporated using a gentle stream of nitrogen gas. The test tube was placed in a desiccator attached to a vacuum pump for at least half an hour. Then the peptides (CASLO, Denmark) dissolved in methanol were added in an appropriate amount according to the desired peptide:lipid ratio. Methanol was again evaporated by a gentle stream of nitrogen gas. The test tube was again placed in a desiccator attached to a vacuum pump for at least another 4 h. The dried lipid film was resuspended by vortexing in 0.5 mL of HBS (50 mM HEPES, 150 mM NaCl, pH 7.4) and went through 10 cycles of freeze and thaw. Then the lipid solution was extruded 30 times through a 0.2-*μ*m membrane using an extruder (Avestin). Final lipid concentration is 1 mM.

#### Fluorescence assay

Liposomes were diluted into final concentration of 0.13 mM into HPS buffer (50 mM HEPES pH 7.4, 150 mM NaCl) in a fluorimetric cuvette, and fluorescence was monitored under constant stirring (1000 rpm) at 20°C in a temperature-controlled Spectrofluorometer Horiba Duetta (lex = 470 nm, lem = 530 nm, excitation band pass 5 nm, emission band pass 10 nm, time increment 10 s, integration time 0.1 s, emission increment 0.5 nm). The sample was equilibrated at least for 45 min before proceeding with the assays. Then 25 *μ*L of 1 M sodium dithionite (Sigma Aldrich) (final concentration of dithionite was 20 mM), freshly prepared in 0.5 M Tris (pH = 10), was added after 500 s of measurement. Three independent measurements were performed for each system.

## Results

**Figure 2:**
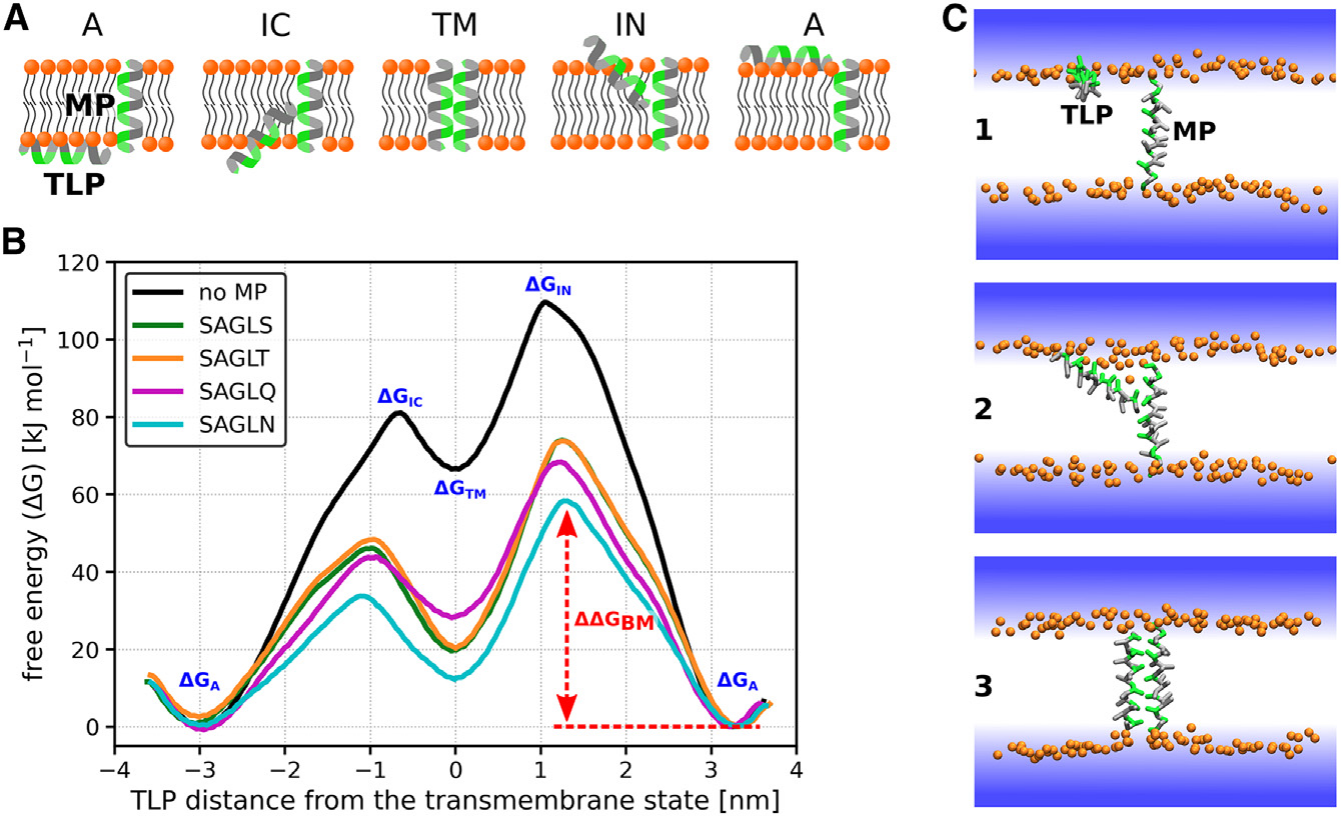
Translocation of the LS9 peptide along MPs containing various hydrophilic residues. *A*) The schematic mechanism of the TLP translocation along the MP. Initially, the TLP is adsorbed on the membrane’s surface, aligned parallel with the surface. As it inserts, it changes orientation to become perpendicular to the membrane surface. Throughout this process, the TLP’s hydrophilic patch orients toward the hydrophilic patch of the MP. (*B*) Free-energy profiles for TLP LS9 translocating alone or along MPs featuring various hydrophilic patch residues: serines (SAGLS), threonines (SAGLT), glutamines (SAGLQ), or asparagines (SAGLN). Notations $\Delta G_{A}$, $\Delta G_{\text{IC}}$, $\Delta G_{\text{IN}}$, and $\Delta G_{\text{TM}}$ refer to the free-energy differences for the adsorbed state (A), local maxima for N and C terminus insertion (IN and IC), and the TLP’s transmembrane state (TM), respectively, with the adsorbed state as a reference. The translocation free-energy barrier in the presence of MP SAGLN, $\Delta \Delta G_{\text{BM}}$, is highlighted with a red arrow, representing the difference between the profile’s highest and lowest free-energy value. Profile errors are estimated to be under 5 kJ mol^−1^, based on the differences between adsorbed states on both sides of the symmetric membrane. (*C*) Simulation snapshots depicting typical configurations of the system when the TLP is 1) in the adsorbed state, 2) inserting into the membrane, and 3) in the transmembrane state. Orange spheres signify lipid phosphates. Lipid tails are omitted for clarity, whereas TLPs’ and MPs’ hydrophilic and hydrophobic residues appear in green and gray, respectively. Water is indicated by a blue gradient. To see this figure in color, go online.

Using the coarse-grained Martini 2 force field (12,13,14), we explored the translocation of an amphiphilic *α*-helical peptide (TLP) across a phospholipid POPE:POPG (3:1) bilayer in the presence of various *α*-helical transmembrane proteins/peptides (MPs). Our study utilized a specific 21-amino acid long TLP, referred to as LS9 due to its composition containing nine serines and 12 leucines. We examined over 20 different MPs with diverse amino acid compositions. Our investigation centered on pinpointing the optimal translocation-enhancing sequence by evaluating several key properties of the MPs: 1) the type of hydrophilic residues, 2) depth of hydrophilic residues within the structure, 3) compactness of the hydrophilic patch, 4) presence of charged residues, and 5) type of hydrophobic residues. In all cases, the TLP translocated the membrane employing the same pathway described previously (5,39,40,41,42) and depicted in Fig. 2 *A*.

### Type of hydrophilic residues

In a prior study (5), we found that the presence of hydrophilic residues in the MP sequence was crucial for enhancing translocation. Consequently, our primary focus was optimizing the hydrophilic amino acids within the MP. We ran simulations on four distinct systems. Each system featured an MP with a hydrophilic patch made of a unique set of hydrophilic residues organized close to each other along the main helical axis. The tested hydrophilic resides were: serines (SAGLS), threonines (SAGLT), glutamines (SAGLQ), and asparagines (SAGLN). The sequences of the MPs used can be found in Table 1.

As illustrated in Fig. 2 *B*, all MPs reduce the free energy of the TLP throughout the membrane region, aiding in the stabilization of the TLP during both its insertion and in its transmembrane state. To measure the ease of TLP translocation across the membrane, we utilized the translocation barrier ($\Delta \Delta G_{\text{BM}}$), which is determined by the difference between the highest and lowest free energy in the profile. Each of the four MPs lowers the $\Delta \Delta G_{\text{BM}}$ in comparison to translocation without an MP, indicating that TLP’s passage through the membrane is facilitated in the presence of these MPs.

The amino acid composition of the MP can influence the free-energy profiles of the TLP. Specifically, asparagines (in MP SAGLN) offer considerably greater stabilization for the TLP compared to serines, threonines, and glutamines. Although the glutamine MP (SAGLQ) reduces the insertion barrier of the TLP relative to the serine and threonine MPs, it slightly undermines the stability of the TLP’s transmembrane state, meaning that the free energy of the TLP’s transmembrane state is higher. This decreased stability arises from the reduced enthalpic stabilization provided by the glutamines within the MP (see Fig. S1). A summary of free-energy differences in the translocation profiles can be found in Table S6.

The reduction in the translocation barrier is primarily due to the membrane disruption induced by the MP. This membrane disruption manifests itself as membrane thinning, slight disorder in the acyl chains (see Fig. S2), and water insertion into the membrane. Note that we observed no continuous water channels or pores around any of the simulated MPs in the membrane (see Fig. S3). We measured the membrane disruption by calculating the water defect in the lipid bilayer surrounding each simulated MP (see Table S6 and the “materials and methods” section for details). A strong correlation between the observed water defects and the $\Delta \Delta G_{\text{BM}}$ values is evident, with a correlation coefficient of −0.98. Generally, increased hydrophilicity in the residues that constitute the hydrophilic patch results in a more pronounced water defect, which, in turn, contributes to decreased translocation barriers.

We opted not to determine the free energy of TLP adsorption/desorption, as our prior research (5) showed that similar *α*-helical MPs did not influence the stability of the TLP’s adsorbed states. Consequently, we view these adsorbed states as consistent reference points for the membrane translocation process.

#### Depth of hydrophilic residues

To explore the connection between translocation enhancement and the placement of hydrophilic residues along the *α* helix, we designed four *α*-helical MPs. Each MP was made up of 21 leucines and two asparagines. These MPs were labeled as LNd3, 6, 9, and 12, where the number indicates the average residue depth of the asparagines. A value of 3 signifies that the asparagines are positioned near the N terminus of the MP, whereas 12 indicates proximity to the MP’s center. The sequences of these MPs are detailed in Table 1.

**Figure 3:**
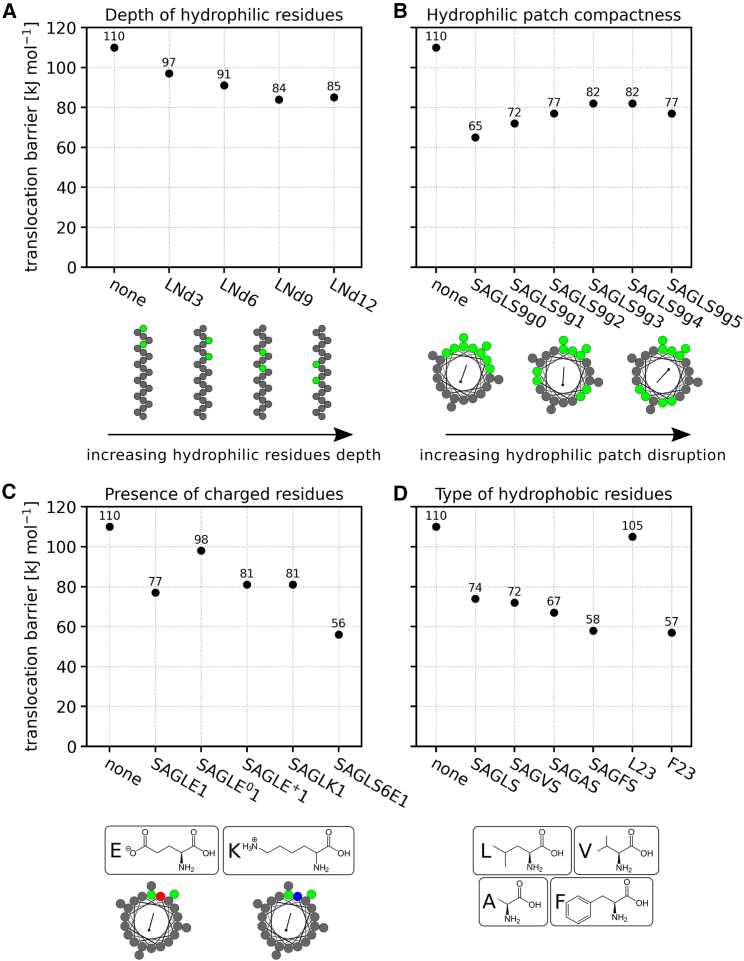
Translocation barriers for TLP LS9 in systems with various MPs. (*A*) Dependence of translocation barriers on the depth of MP’s hydrophilic residues. As the hydrophilic residues become positioned deeper in the membrane, barriers for TLP translocation generally decrease. (*B*) Dependence of translocation barriers on the compactness of the MP’s hydrophilic patch. Increasing disruption of the hydrophilic patch results in increased translocation barriers. (*C*) Dependence of translocation barriers on the presence of a charged residue in the MP. The introduction of charged amino acid side chains significantly decreases the translocation barrier for the TLP. (*D*) Dependence of translocation barriers on the hydrophobic residues present in the MP. Phenylalanine stands out as the most effective in enhancing TLP translocation among the residues studied. For detailed free-energy profiles of TLP translocation in the presence of these MPs, see the supporting material. To see this figure in color, go online.

Placing the hydrophilic residues closer to the MP’s center, and thus closer to the center of the membrane, results in reduced translocation barriers compared to when these residues are situated near the MP’s termini. Refer to Fig. 3 *A* and Table S8 for an overview of the relevant free-energy differences.

The decrease of translocation barriers with the deeper insertion of the MP’s hydrophilic residues does not align with the water defects observed around each MP. For example, LNd12 produces a notably smaller water defect (42.1 arbitrary units) compared to MP LNd3 (44.0 arbitrary units), even though LNd12 exhibits significantly greater translocation-enhancing properties. As illustrated in Fig. S4 *A*, hydrophilic and charged residues located either at the membrane surface or at its center lead to smaller water defects compared to those placed at an intermediate depth within the membrane. On the other hand, MPs with centrally located hydrophilic/charged residues reduce the density of lipid tails in the membrane’s center, as indicated in Fig. S4 *B* and in Fig. S5, but do not affect the water’s ability to insert into the membrane core. See Table S7 for the sequences of MPs used for this additional characterization. By utilizing artificially disrupted membranes, we show that this tail defect (TD) accounts for a considerable reduction in the translocation barrier, especially for MPs with hydrophilic/charged residues located near the membrane’s center (see Fig. S4 *D* and *E*).

Interestingly, even though the MPs discussed in this section were asymmetric, we did not find significant variations in the free energies when comparing different directions of translocation through the membrane, as shown in Fig. S6.

#### Hydrophilic patch compactness

In this section, we explored the effect of hydrophilic patch compactness, i.e., the arrangement of hydrophilic residues around the *α* helix of the MP, on the translocation enhancement of TLP. By analyzing six MPs composed of leucine and serine residues with different distributions around the *α* helix, we determined that the compactness of the hydrophilic patch is crucial for effective translocation enhancement. The MPs examined are labeled as SAGLS9g0–5, where the concluding number represents the extent of disruption in their hydrophilic patch. Refer to Table 1 for the sequences of these MPs and to Fig. S7 *A* for their helical wheel diagrams.

MPs with hydrophilic residues oriented in the same direction (those with compact patches) enhance translocation more effectively than MPs where hydrophilic residues are oriented in diverse directions (those with disrupted patches). Even a minor disruption in the hydrophilic patch’s compactness, as seen in SAGLS9g1, can lead to substantially elevated translocation barriers compared to a fully compact patch (see Fig. 3 *B*). As the level of patch disruption intensifies, $\Delta \Delta G_{\text{BM}}$ values continue to rise up to a certain threshold. $\Delta \Delta G_{\text{BM}}$ value for the TLP in the presence of SAGLS9g5 is then somewhat reduced again, which can be attributed to the formation of a secondary hydrophilic patch by the MP’s hydrophilic residues (see Fig. S8). The free-energy profiles of TLP translocation along these MPs can be found in Fig. S7 and a breakdown of the free-energy differences is provided in Table S9.

Two primary factors are responsible for the reduced translocation enhancement by MPs with disrupted hydrophilic patches. Firstly, the membrane disruption induced by the hydrophilic residues is not as concentrated in the region where the TLP translocates. Secondly, MPs with notably disrupted hydrophilic patches have weaker interactions with the TLP, resulting in less enthalpic stabilization for the TLP during its translocation, as depicted in Fig. S7 *C*.

#### Presence of charged residues

To understand the influence of charged residues on translocation enhancement, we examined five distinct polyleucine MPs containing one charged residue in their center. Among these, SAGLE1 featured a negatively charged glutamate, whereas SAGLK1 incorporated a positively charged lysine. Two variations of SAGLE1 were also investigated: SAGLE^0^1 with its glutamate protonated (rendering it uncharged) and SAGLE^+^1, which contained an artificial positively charged counterpart termed “anti-glutamate.” Additionally, MP SAGLS6E1 was derived from SAGLS, where one centrally located serine was substituted with a negatively charged glutamate. The sequences of these MPs are detailed in Table 1.

A single charged residue within MPs significantly affects TLP translocation (see Fig. 3 *C*). The inclusion of just one charged residue, be it glutamate, lysine, or anti-glutamate, affects the translocation barrier nearly as much as having five serines in the SAGLS sequence. Incorporating a charged residue into an MP already possessing a hydrophilic patch, as with MP SAGLS6E1, further amplifies its ability to enhance translocation. For computed free-energy profiles and free-energy differences, refer to Fig. S9 *A* and Table S10, respectively.

The pronounced influence of glutamate, lysine, and anti-glutamate primarily arises from the charge of their side chains. Neutralizing the charge markedly diminishes the MP’s capability to enhance translocation. These charged side chains induce a significant TD, as detailed in Table S10, accounting for this enhancement in translocation. However, the charge of these residues does not notably alter the interaction strength between the TLP and the MP, as indicated in Fig. S9 *B*.

#### Type of hydrophobic residues

To assess the impact of hydrophobic residues on translocation enhancement, we designed six MPs primarily varying in their hydrophobic residue composition. We compared the effects of leucine (SAGLS, L23), phenylalanine (SAGFS, F23), valine (SAGVS), and alanine (SAGAS). The MPs L23 and F23 consisted solely of leucines and phenylalanines, respectively. In contrast, the other MPs incorporated a hydrophilic serine patch. Refer to Table 1 for the sequences of the MPs under consideration.

Rather unexpectedly, hydrophobic residues can markedly influence translocation enhancement, as illustrated in Fig. 3 *D*. Although leucines and valines demonstrate similar translocation-enhancing capabilities, alanines marginally reduce the TLP’s translocation barrier when compared to leucines. Phenylalanines, however, have the most pronounced impact on TLP translocation. This is further demonstrated by MP F23, which consists solely of phenylalanines. F23 proved to be a very strong translocation enhancer, in contrast to MP L23, a fully hydrophobic peptide composed of 23 leucines. See Fig. S10 *A* for the calculated free-energy profiles and Table S11 for the relevant free-energy differences in the profiles.

Phenylalanine’s marked influence on translocation enhancement arises from a combination of two factors. Firstly, the presence of phenylalanines induces a notable membrane disruption, as observed with both F23 and SAGFS (see Table S11). This disruption is likely attributed to the bulky nature of phenylalanine’s side chains, which interfere with the lipid packing of the membrane. Secondly, the aromatic nature of the phenylalanine side chains facilitates strong interactions with the hydrophobic residues of the TLP, resulting in substantial enthalpic stabilization, as shown in Fig. S10 *B*.

#### Optimizing the MP sequence

We designed several MPs that combined multiple translocation-enhancing properties and evaluated their effect on TLP translocation. ENHTM1 features a compact hydrophilic patch composed of asparagines, a centrally positioned charged glutamate, and phenylalanines as hydrophobic residues. ENHTM2 is similar to ENHTM1 but has a smaller hydrophilic patch and contains three glutamates instead of one. ENHTM3 possesses more modest features, with leucines, a large and compact hydrophilic patch composed of serines, and a single positively charged lysine at the center of the peptide. The sequences of these MPs are detailed in Table 1.

**Figure 4:**
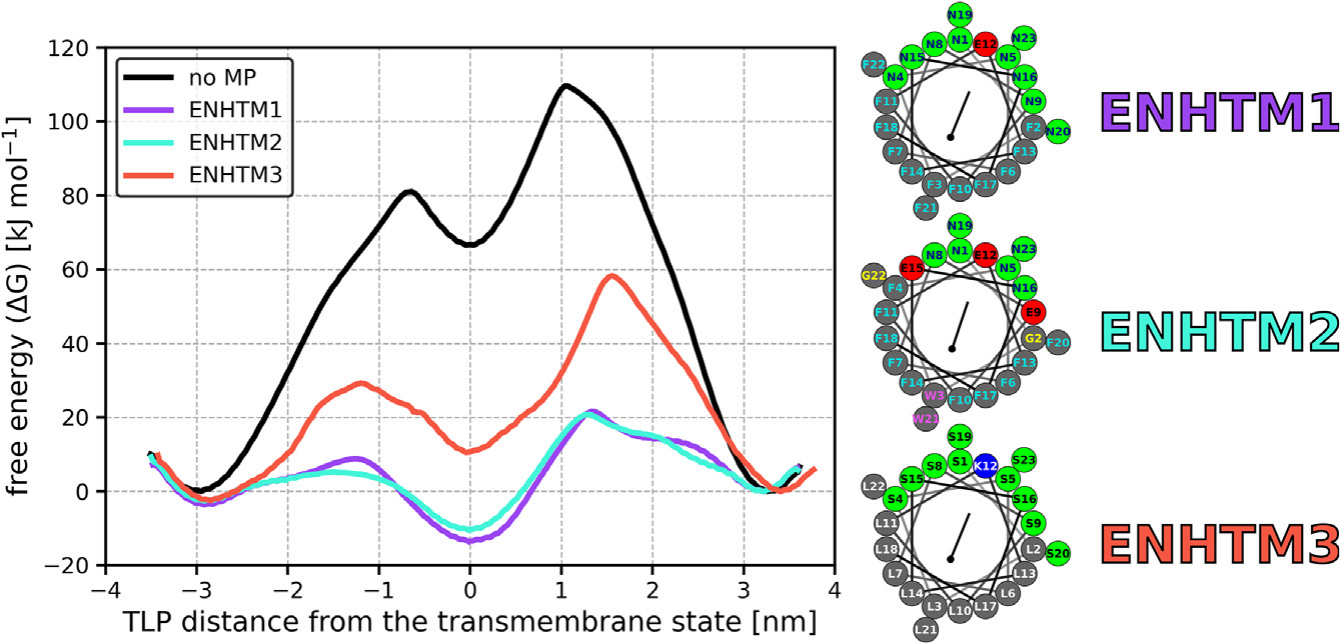
Comparison of MPs combining multiple translocation-enhancing properties. Free-energy profiles of TLP LS9 translocating through the membrane alone (*black*) or in the presence of MP ENHTM1 (*purple*), ENHTM2 (*teal*), or ENHTM3 (*red*). Profile errors are below 5 kJ mol^−1^ based on the asymmetry of the adsorbed states. ENHTM1 and ENHTM2 decrease the translocation barrier more than any other simulated MP. The helical wheels showing the composition of the MPs and the position of their residues are shown to the right of the chart. To see this figure in color, go online.

As demonstrated in Fig. 4, all of the MPs significantly reduce the translocation free-energy barrier of the TLP. ENHTM1 and ENHTM2 offer similar stabilization with translocation barriers of just 35 and 31 kJ mol^−1^, respectively. ENHTM3 has a translocation barrier of 58 kJ mol^−1^, which is comparable to the MP SAGLS6E1 mentioned earlier.

Overall, these results suggest that the translocation-enhancing properties described herein are largely additive and can be synergistically combined for further translocation enhancement.

#### Exploring the relationship between $\Delta \Delta G_{\text{BM}}$ and $\Delta G_{\text{TM}}$

To further explore the translocation-enhancing abilities of the MPs, we examined the relationship between the translocation barriers, $\Delta \Delta G_{\text{BM}}$, and the free energy of transmembrane states, $\Delta G_{\text{TM}}$, across all simulated systems. As shown in Fig. S11, we found that these properties are linearly correlated, indicating that there are no MPs that stabilize the insertion barrier of the TLP without also stabilizing its transmembrane state, or vice versa. This implies that the difficulty of the TLP translocating along an MP, given by the height of the insertion maxima, can be estimated by analyzing the stability of the TLP’s transmembrane state compared to its adsorbed state.

#### Translocation across POPC membranes

All of our above simulations employed a POPE:POPG (3:1) membrane to mimic the bacterial inner membrane. For several MPs, we also explored TLP translocation across a POPC membrane. We observed that trends for POPC membranes mirrored those for POPE:POPG membranes, with stronger translocation enhancement being associated with larger membrane disruption. Overall, translocation of the LS peptide through the POPC membrane was easier, with translocation barriers consistently roughly 10 kJ mol^−1^ lower than those in the corresponding POPE:POPG systems. See Fig. S12 and Table S12 for the free-energy profiles and free-energy differences calculated for systems with the POPC membrane.

#### Lipid scrambling by translocation enhancers

Finally, we probed the potential of a chosen MP, ENHTM3, to promote the translocation of other amphiphilic molecules, specifically phospholipids. We opted for the ENHTM3 over the ENHTM1 and ENHTM2 MPs. This decision was underpinned by its feasibility for synthesis for subsequent experimental validation and by its predicted greater propensity to form an *α* helix, attributed to the presence of leucines rather than phenylalanines. Indeed, the ENHTM3 remained in *α*-helical conformation during our atomistic simulations described below.

**Figure 5:**
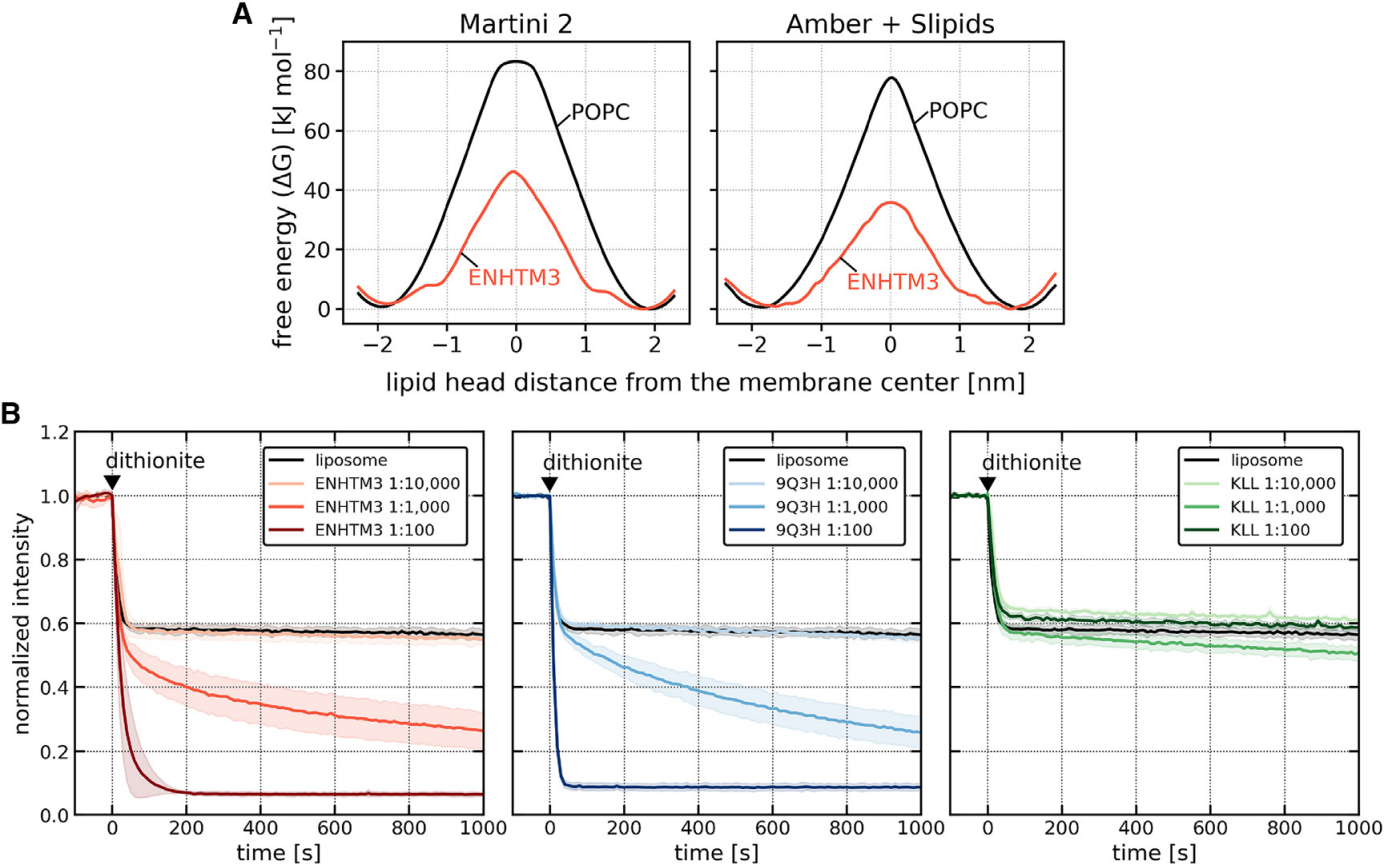
Lipid flip-flop facilitated by MPs. (*A*) Free-energy profiles of lipid flip-flop in a pure POPC bilayer (*black curve*) versus a POPC membrane with the embedded MP ENHTM3 (*red curve*). The left chart presents results from the coarse-grained Martini 2 force field, whereas the right chart corresponds to the atomistic Amber + Slipids force field. (*B*) Time course of NBD-PC fluorescence, normalized to its initial value, after dithionite addition. Black curves reflect fluorescence in pure LUVs, whereas colored curves represent LUVs containing peptide ENHTM3 (*left*), 9Q3H (*middle*), or KLL (*right*) at three distinct peptide:lipid (mol:mol) ratios. Decreased fluorescence intensity is indicative of increased scrambling rate. Each line represents the mean normalized intensity derived from three independent measurements, with the shading indicating ±1 standard deviation from the mean. To see this figure in color, go online.

Our initial step involved calculating the free energy associated with the flip-flop of a POPC lipid when facilitated by the MP ENHTM3, employing both the coarse-grained Martini 2 force field and the atomistic Amber 99SB-ILDN force field complemented with Slipids 2020 force field. Fig. 5 *A* illustrates a pronounced reduction in the free-energy barrier for the flip-flop of POPC in the presence of ENHTM3 across both computational models (with a decrease from 83 to 46 kJ mol^−1^ in Martini 2, and from 78 to 36 kJ mol^−1^ in Amber + Slipids).

This computational observation was subsequently validated through a fluorescence assay employing NBD-PC lipids embedded within large unilamellar vesicles (LUVs) (10,43). For this experimental validation, we also used a polyleucine MP KLL, devoid of any translocation-enhancing attributes, as a benchmark for negative control. Simultaneously, lipid-scrambling peptide 9Q3H (8) was introduced as a positive control. See Table 1 for the sequences of these peptides. Experimentally, the scrambling rate recorded for ENHTM3 was similar to that observed for the positive control 9Q3H, as seen in Fig. 5 *B*. In contrast, the KLL peptide, our negative control, exhibited negligible lipid scrambling regardless of its concentration.

These findings demonstrate that the general features of translocation-enhancing MPs assist not only in peptide translocation but also in inducing lipid flip-flop, and the simulation results of decreased free-energy barriers are in agreement with experiments.

## Discussion

We investigated the effect of various properties of *α*-helical transmembrane proteins/peptides (MPs) on the membrane translocation of a representative leucine-serine *α*-helical TLP utilizing the Martini 2 coarse-grained model. Our analysis centered on the type of the hydrophilic residues, their position along the *α* helix (residue depth), the compactness of the hydrophilic patch of the MP, the incorporation of charged residues, and the type of hydrophobic residues within the MP. Each simulation specifically addressed the translocation of one TLP along one MP, underscoring our focus on the translocation process at low peptide concentrations. We focused on peptide translocation across POPE:POPG (3:1) membranes, which mimic bacterial inner membranes, and also conducted several simulations with POPC membranes. No qualitative differences were observed between the two. We postulate that our general conclusions are valid for all biologically relevant phospholipid membrane types.

We observed the same translocation pathway as in our prior research (5), where the TLP, initially adsorbed on the membrane surface in orientation parallel with the membrane plane, shifts to a perpendicular orientation in the transmembrane state while interacting with the MP. This mode of translocation aligns with findings from previous peptide translocation studies (26,39,40,42,44). However, it is worth noting that nonamphiphilic or non-*α*-helical TLPs might navigate through the phospholipid bilayer differently based on their distinct attributes, such as using the mechanism outlined for NAF-1 (45).

Based on our simulations, peptide translocation through biological membranes can be facilitated by incorporating an MP with specific properties into the membrane. These properties encompass the presence of hydrophilic, charged, and/or aromatic hydrophobic residues. Although we have tested only phenylalanines among the aromatic residues, it is reasonable to assume that other similar aromatic residues, such as tryptophan, would exhibit similar behavior.

As previously demonstrated, increasing the number of hydrophilic residues in the MP’s sequence increases its translocation-enhancing abilities (5). In addition to this, the translocation enhancement can further be improved by incorporating hydrophilic residues with lower hydrophobicity, such as asparagines in place of serines. This observation is somewhat applicable to hydrophobic residues as well; for instance, using the less hydrophobic alanines instead of leucines slightly elevates the MP’s translocation-enhancing abilities. Nonetheless, it is important to highlight that phenylalanine remains a significantly more effective translocation-enhancing residue than alanine, despite its higher hydrophobicity.

The arrangement of translocation-enhancing residues within the MP’s structure is also crucial. For optimal translocation enhancement, the hydrophilic residues need to form a patch that guides the TLP’s translocation. Spreading hydrophilic residues in varying patterns around the MP’s *α* helix consistently results in reduced translocation enhancement. Additionally, for superior translocation enhancement, it is more beneficial to position the hydrophilic or charged residues near the membrane’s center rather than its surface.

We observed that the employed MPs generally stabilize both the insertion barrier and the transmembrane state of the TLP. This indicates that the translocation ability of the TLP can be estimated by examining the stability of its transmembrane state, eliminating the need to calculate the entire translocation pathway. However, this method should be limited to comparing the translocation of a single TLP along various MPs. It is not suitable for comparing the translocation abilities of different TLPs, as the linear relationship between translocation difficulty and the stability of the transmembrane state often does not apply to them (26).

Increasing the translocation-enhancing capabilities of an MP can often result in two outcomes: 1) reduced MP stability within the membrane, and 2) a decrease in the *α*-helical propensity of the MP. Concerning the first challenge, it is essential to strike a careful balance between the MP’s stability in the membrane and its translocation-enhancing properties. In our studies, all the MPs from Table 1 remained stable in their transmembrane states in the POPE:POPG (3:1) membrane. Additionally, we propose that MPs with even more potent translocation-enhancing features, perhaps due to the addition of more hydrophilic/charged residues, might be viable if incorporated into a larger, adequately hydrophobic protein structure. This would anchor the translocation-enhancing segment securely within the membrane.

Given the constraints of the employed coarse-grained model, which maintains the secondary structure of our MPs and TLPs during simulations, we have not explored the second concern regarding the *α*-helical propensity of the MPs. Still, we postulate that the identified translocation-enhancing attributes are also applicable to MPs without an *α*-helical conformation in their transmembrane part as well as to more extensive protein structures. This notion stems from the fact that the guiding geometrical principles, such as the ideal position and orientation of the translocation-enhancing residues within the structure, are not reliant on a specific secondary structure. Instead, they are based on the relative positioning of residues concerning one another and the membrane’s normal.

In addition to establishing the guidelines for designing translocation-enhancing MPs, we delved into the mechanistic underpinnings of the translocation enhancement. In our previous work (5), we demonstrated that translocation-enhancing MPs disrupt the membrane, leading to water insertion into the membrane without the formation of continuous water channels. This phenomenon can be measured by evaluating the water defect (i.e., the average water density surrounding the MP). Disrupted membrane offers a more accessible path for the TLP, resulting in a strong correlation between the water defect and the MP’s translocation-enhancing capacities. Formation of a water defect, even if formed by the TLP itself, has, in fact, been recently suggested to enhance the translocation of charged peptides (46). Additionally, water defect is related to membrane thinning, which has been proposed to enhance the insertion and translocation of proteins by certain protein translocases (47). Other than water defect, our study also revealed that the MP offers enthalpic stabilization to the TLP during translocation, stemming from the attractive interactions between the residues of the TLP and MP.

In this study, we not only validate our prior observations but also demonstrate that, when the membrane is disrupted away from its surface, this disturbance does not always appear as a water defect. Instead, it can manifest as a reduced density of lipid tails at the membrane’s core. Such a tail defect (TD) still plays a pivotal role in translocation by making it easier for the TLP to traverse the membrane. Furthermore, we underscore that enthalpic stabilization does not solely depend on interactions between hydrophilic residues. Aromatic residues, particularly phenylalanine, can also provide potent enthalpic stabilization to the TLP.

We propose that enhancement of translocation originates from three primary factors: 1) wet membrane disruption, i.e., membrane thinning, which relates to water insertion into the membrane core and arises due to translocation-enhancing residues positioned sufficiently close to the membrane surface; 2) dry membrane disruption, which does not relate to water insertion and is attributed to centrally positioned translocation-enhancing residues; and 3) stabilizing interactions between the TLP and the MP.

It should be noted that the membranes used in this study were all symmetric in their lipid composition, whereas many biological membranes are asymmetric (48,49). In asymmetric membranes, the disruption caused by MPs in each membrane leaflet could vary, leading to complex effects on the TLP translocation. Furthermore, TLPs have been reported to be affected by membrane asymmetry during the translocation process (50). Although we expect that our general conclusions would hold for all phospholipid membranes, a detailed exploration of the relationship between MPs and asymmetric membranes would need further research.

The design of our MPs drew inspiration from scramblases, proteins that enable the bidirectional flip-flop of phospholipids (6,7,8,9,10). We therefore tested scrambling activity of one of our MPs using coarse-grained simulations, all-atom simulations, and fluorescence experiments. In the presence of this MP, we saw a substantial reduction in the flip-flop barrier and an accelerated rate of scrambling. These results indicate that translocation-enhancing properties can also boost phospholipid flip-flop. We propose that the translocation-enhancing properties and the structural mechanisms we discussed might be typical for proteins aiding in both peptide membrane insertion and lipid scrambling. Indeed, recent experimental data, unbiased molecular dynamics simulations (51), and free-energy calculations (52) suggest that insertases, which assist in embedding proteins or protein domains into membranes, can also promote lipid scrambling through their hydrophilic patches or cavities. Additionally, a very recent preprint hints at scramblases being able to also facilitate the translocation of amphipathic drug molecules (53). This introduces the possibility that the mechanisms facilitating peptide translocation and lipid scrambling might extend to the membrane translocation of amphiphiles in general.

Finally, we suggest that scramblases, insertases, or similar proteins naturally occurring in biological membranes could be exploited by antimicrobial peptides or CPPs to more easily enter the interior of the cell. Alternatively, peptide mixtures containing translocation enhancers could be designed for enhanced peptide transport into cells. Given that the majority of natural AMPs carry a positive charge (54), utilizing specifically charged MPs—either positive or negative—could result in selective translocation enhancement, offering more precise control over the peptide translocation process. Exploring this option, however, falls outside the scope of this study.

## Conclusions

We explored the influence of transmembrane proteins/peptides with diverse properties on peptide translocation using coarse-grained molecular dynamics coupled with free-energy calculations. Our findings reveal that the presence of a compact hydrophilic patch, charged residues, and aromatic residues with bulky side chains in the transmembrane protein/peptide significantly enhances peptide translocation across the membrane. Based on this, we offer detailed guidelines for designing translocation-enhancing transmembrane proteins/peptides.

Moreover, we pinpoint three primary mechanisms underlying this enhanced translocation: membrane disruption linked to water defects, membrane disruption without water defects but with reduced lipid tail density, and stabilizing interactions between peptides/proteins.

Through both coarse-grained and atomistic free-energy calculations, complemented by experimental fluorescence assays, we demonstrate that these translocation-enhancing properties also aid in phospholipid scrambling. This leads us to postulate that the characteristics we have outlined might be ubiquitous among proteins that facilitate the insertion or translocation of amphiphilic molecules into or across phospholipid membranes, such as scramblases and insertases.

We further propose that scramblases and insertases could be exploited to enhance the transport of antimicrobial peptides or CPPs through cell membranes. Another prospect is the potential synergistic effect of using a pair of peptides: a highly hydrophobic peptide with a hydrophilic patch and/or charged residues could embed into the membrane and subsequently enhance the translocation of another, more hydrophilic peptide.

## Author contributions

L.B. carried out the molecular dynamics simulations and analyzed the data. M.D. performed the fluorescence assay experiments. R.V. designed the research. L.B., M.D., and R.V. wrote the article.

## Supporting Material

Document S1. Figures S1–S12 and Tables S1–S12

Document S2. Article plus Supporting Material
