# Supplementary material for: Optimizing properties of translocation-enhancing transmembrane proteins
Source: Biophys J. 2024 Apr 13;123(10):1240–52. doi: 10.1016/j.bpj.2024.04.009 (PMC11140465; doi:10.1016/j.bpj.2024.04.009)
Supplement: Document S1. Figures S1–S12 and Tables S1–S12 [file mmc1.pdf]

Biophysical Journal, Volume *123*

Supplemental information

Optimizing properties of translocation-enhancing transmembrane proteins

Ladislav Bartoš, Martina Drabinová, and Robert Vácha

SUPPORTING MATERIAL

Optimizing properties of translocation-enhancing transmembrane proteins

Ladislav Bartoš, Martina Drabinová, Robert Vácha

Table S1: Distribution of umbrella sampling windows along the collective variable with biasing force constants used for the peptide translocation simulations with POPE:POPG (3:1) membranes. Reference distances are in nm, force constants in kJ mol^{-1} nm^{-2}.

Reference distance	Force constant	Reference distance	Force constant
2.10	3000	0.08	8000
2.00	3000	0.04	8000
1.90	3000	0.00	8000
1.80	3000	−0.04	8000
1.70	3000	−0.08	8000
1.60	3000	−0.12	8000
1.50	3000	−0.16	8000
1.40	3000	−0.20	8000
1.30	3000	−0.24	8000
1.20	3000	−0.28	8000
1.10	3000	−0.32	8000
1.00	3000	−0.36	8000
0.90	3000	−0.40	8000
0.80	3000	−0.43	8000
0.70	3000	−0.46	8000
0.60	3000	−0.50	8000
0.55	5000	−0.55	8000
0.50	8000	−0.60	8000
0.46	8000	−0.65	5000
0.42	8000	−0.70	3000
0.38	8000	−0.80	3000
0.36	8000	−0.90	3000
0.34	8000	−1.00	3000
0.32	8000	−1.10	3000
0.30	8000	−1.20	3000
0.28	8000	−1.30	3000
0.26	8000	−1.40	3000
0.24	8000	−1.50	3000
0.22	8000	−1.60	3000
0.20	8000	−1.70	3000
0.16	8000	−1.80	3000
0.12	8000	−1.90	3000

Table S2: Distribution of umbrella sampling windows along the collective variable with biasing force constants used for the peptide translocation simulations with POPC membranes. Reference distances are in nm, force constants in kJ mol^{-1} nm^{-2}.

Reference distance	Force constant	Reference distance	Force constant
2.10	1000	0.15	5000
2.00	1000	0.10	5000
1.90	1000	0.05	5000
1.80	1000	0.00	5000
1.70	1000	−0.05	5000
1.60	1000	−0.10	5000
1.50	1000	−0.15	5000
1.40	1000	−0.20	5000
1.35	3000	−0.25	5000
1.30	3000	−0.30	5000
1.25	3000	−0.35	5000
1.20	3000	−0.40	5000
1.15	3000	−0.45	5000
1.10	3000	−0.50	3000
1.05	3000	−0.55	3000
1.00	3000	−0.60	3000
0.95	3000	−0.65	3000
0.90	3000	−0.70	3000
0.85	3000	−0.75	3000
0.80	3000	−0.80	3000
0.75	3000	−0.85	3000
0.70	3000	−0.90	3000
0.65	3000	−0.95	3000
0.60	3000	−1.00	1000
0.55	3000	−1.10	1000
0.50	3000	−1.20	1000
0.45	5000	−1.30	1000
0.40	5000	−1.40	1000
0.35	5000	−1.50	1000
0.30	5000	−1.60	1000
0.25	5000	−1.70	1000
0.20	5000	−1.80	1000

Table S3: Distribution of umbrella sampling windows along the collective variable with biasing force constants used for Martini 2 lipid scrambling simulations. Reference distances are in nm, force constants in kJ mol^{-1} nm^{-2}.

Reference distance	Force constant	Reference distance	Force constant
2.30	1000	−0.05	4000
2.20	1000	−0.10	4000
2.10	1000	−0.15	3000
2.00	1000	−0.20	3000
1.90	1000	−0.25	3000
1.80	1000	−0.30	3000
1.70	1000	−0.35	2000
1.60	1000	−0.40	2000
1.50	1000	−0.45	2000
1.40	1000	−0.50	2000
1.30	1000	−0.55	2000
1.20	1000	−0.60	2000
1.10	1000	−0.65	2000
1.00	2000	−0.70	2000
0.95	2000	−0.75	2000
0.90	2000	−0.80	2000
0.85	2000	−0.85	2000
0.80	2000	−0.90	2000
0.75	2000	−0.95	2000
0.70	2000	−1.00	2000
0.65	2000	−1.10	1000
0.60	2000	−1.20	1000
0.55	2000	−1.30	1000
0.50	2000	−1.40	1000
0.45	2000	−1.50	1000
0.40	2000	−1.60	1000
0.35	2000	−1.70	1000
0.30	2000	−1.80	1000
0.25	3000	−1.90	1000
0.20	3000	−2.00	1000
0.15	3000	−2.10	1000
0.10	4000	−2.20	1000
0.05	4000	−2.30	1000
0.00	4000		

Table S4: Distribution of umbrella sampling windows along the collective variable with biasing force constants used for atomistic lipid scrambling simulations with pure POPC membrane. Reference distances are in nm, force constants in kJ mol^{-1} nm^{-2}. ^{R} identifies windows in which the Hamiltonian replica exchange was applied.

Reference distance	Force constant	Reference distance	Force constant
2.30	1000	−0.03	2000^{R}
2.20	1000	−0.06	2000^{R}
2.10	1000	−0.09	2000^{R}
2.00	1000	−0.12	2000^{R}
1.90	1000	−0.15	2000^{R}
1.80	1000	−0.18	2000^{R}
1.70	1000	−0.21	2000^{R}
1.60	1000	−0.25	2000^{R}
1.50	1000	−0.30	1000
1.40	1000	−0.40	1000
1.30	1000	−0.50	1000
1.20	1000	−0.60	1000
1.10	1000	−0.70	1000
1.00	1000	−0.80	1000
0.90	1000	−0.90	1000
0.80	1000	−1.00	1000
0.70	1000	−1.10	1000
0.60	1000	−1.20	1000
0.50	1000	−1.30	1000
0.40	1000	−1.40	1000
0.30	1000	−1.50	1000
0.25	2000	−1.60	1000
0.21	2000^{R}	−1.70	1000
0.18	2000^{R}	−1.80	1000
0.15	2000^{R}	−1.90	1000
0.12	2000^{R}	−2.00	1000
0.09	2000^{R}	−2.10	1000
0.06	2000^{R}	−1.20	1000
0.03	2000^{R}	−1.30	1000
0.00	2000^{R}		

Table S5: Distribution of umbrella sampling windows along the collective variable with biasing force constants used for atomistic lipid scrambling simulations with POPC membrane containing ENHTM3. Reference distances are in nm, force constants in kJ mol^{-1} nm^{-2}. ↓ indicates windows with initial configurations from simulations where the lipid translocated from the upper to lower leaflet. ↑ signifies the opposite direction, and ^{R} denotes windows using Hamiltonian replica exchange.

Reference distance	Force constant	Reference distance	Force constant
2.30	1000↓	−0.03	2000↑^{R}
2.25	1000↓	−0.06	2000↓^{R}
2.20	1000↓	−0.09	2000↑^{R}
2.15	1000↓	−0.12	2000↓^{R}
2.10	1000↓	−0.15	2000↑^{R}
2.05	1000↓	−0.18	2000↓^{R}
2.00	1000↓	−0.21	2000↑^{R}
1.95	1000↓	−0.25	2000↓^{R}
1.90	1000↓	−0.30	1000↑
1.85	1000↓	−0.35	1000↑
1.80	1000↓	−0.40	1000↑
1.75	1000↓	−0.45	1000↑
1.70	1000↓	−0.50	1000↑
1.65	1000↓	−0.55	1000↑
1.60	1000↓	−0.60	1000↑
1.55	1000↓	−0.65	1000↑
1.50	1000↓	−0.70	1000↑
1.45	1000↓	−0.75	1000↑
1.40	1000↓	−0.80	1000↑
1.35	1000↓	−0.85	1000↑
1.30	1000↓	−0.90	1000↑
1.25	1000↓	−0.95	1000↑
1.20	1000↓	−1.00	1000↑
1.15	1000↓	−1.05	1000↑
1.10	1000↓	−1.10	1000↑
1.05	1000↓	−1.15	1000↑
1.00	1000↓	−1.20	1000↑
0.95	1000↓	−1.25	1000↑
0.90	1000↓	−1.30	1000↑
0.85	1000↓	−1.35	1000↑
0.80	1000↓	−1.40	1000↑
0.75	1000↓	−1.45	1000↑
0.70	1000↓	−1.50	1000↑
0.65	1000↓	−1.55	1000↑
0.60	1000↓	−1.60	1000↑
0.55	1000↓	−1.65	1000↑
0.50	1000↓	−1.70	1000↑
0.45	1000↓	−1.75	1000↑
0.40	1000↓	−1.80	1000↑
0.35	1000↓	−1.85	1000↑
0.30	1000↓	−1.90	1000↑
0.25	2000↓	−1.95	1000↑
0.21	2000↑^{R}	−2.00	1000↑
0.18	2000↓^{R}	−2.05	1000↑
0.15	2000↑^{R}	−2.10	1000↑
0.12	2000↓^{R}	−2.15	1000↑
0.09	2000↑^{R}	−2.20	1000↑
0.06	2000↓^{R}	−2.25	1000↑
0.03	2000↑^{R}	−2.30	1000↑
0.00	2000↓^{R}		

Type of hydrophilic residues

Table S6: Free energy differences for important points in the translocation profiles of TLP LS9 translocating through a pure membrane or in the presence of MPs containing different hydrophilic residues. Free energy values are shown relative to the TLP in the adsorbed state [kJ mol^{-1}]. The error was estimated to be below 5 kJ mol^{-1} based on the profile asymmetry. Water defect (WD) and tail defect (TD) [arb. u.] observed around the specific MP and the hydrophobicity (H) of the MP are shown in the last three columns of the table.

MP	ΔG_{IC}	ΔG_{TM}	ΔG_{IN}	$\Delta\Delta G_{\mathrm{BM}}$	WD	TD	H
none	81	67	110	110	41.5	N/A	N/A
SAGLS	46	20	74	74	47.3	102.6	0.902
SAGLT	48	20	74	74	47.0	102.6	0.967
SAGLQ	43	28	68	69	48.4	102.2	0.863
SAGLN	34	12	58	58	49.7	102.6	0.780

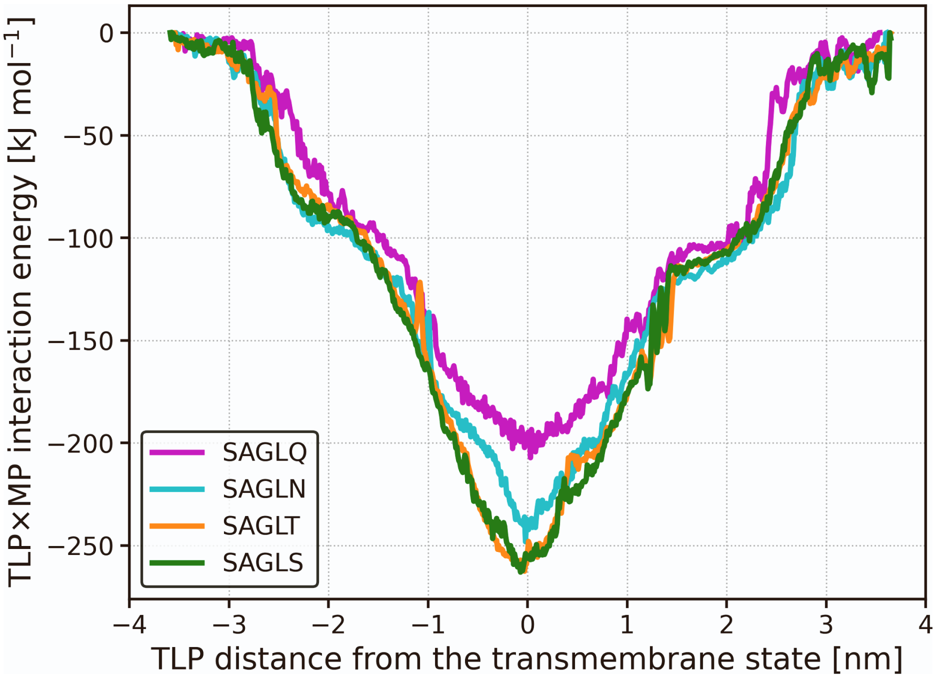

Figure S1: Average strength of the total interaction (Lennard-Jones + coulombic) between the TLP LS9 and MP SAGLQ, SAGLN, SAGLT, or SAGLS calculated from the simulated set of umbrella sampling windows and shown as a function of TLP distance from the transmembrane state. Interaction strength increases as the TLP inserts into the membrane. Note that MP SAGLQ provides significantly weaker enthalpic stabilization for the TLP in the transmembrane state than the other MPs.

Membrane disruption

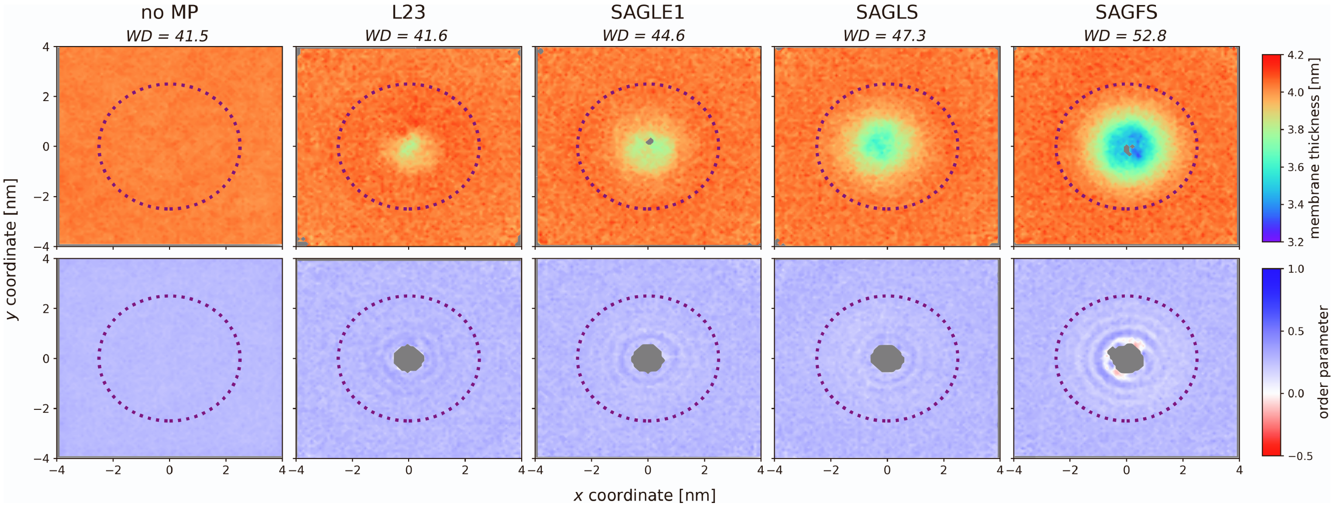

Figure S2: Average membrane thickness (upper row) and coarse-grained order parameters for the C3B-C4B bond of POPE lipids (lower row) calculated for systems both without an MP and with various MPs (L23, SAGLE1, SAGLS, and SAGFS). The water defect calculated for each system is indicated above each chart, beneath the MP's name. The purple circle in each chart highlights the area where the water defect was calculated. Areas in gray indicate insufficient data for calculating the property of interest. A clear correlation is visible between membrane thinning around the MP and the water defect. Disorder in the lipid acyl chains near the MPs, especially notable for the phenylalanine-containing SAGFS peptide, is also visible, though it is generally minor. The membrane thickness maps were generated using the `memthick` tool, available at github.com/Ladme/memdian, and the maps of coarse-grained order parameters using the `ordermap` tool, available at github.com/Ladme/order.

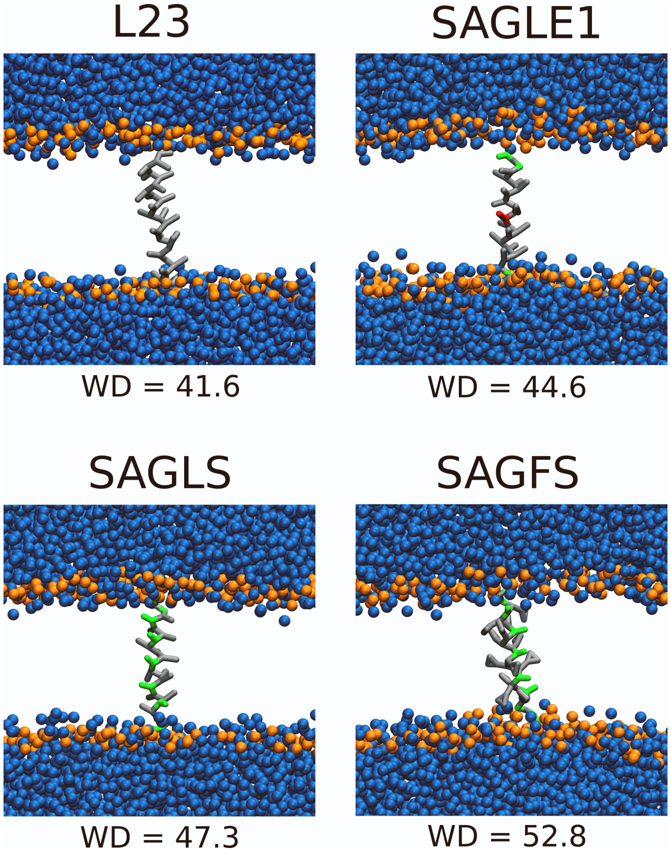

Figure S3: Representative snapshots of MPs L23, SAGLE1, SAGLS, and SAGFS simulated in POPE:POPG (3:1) membranes without the presence of TLP showing that no water channels or pores develop during the simulations with any MPs. Orange and blue spheres represent lipid phosphates and water, respectively. Lipid tails are omitted for clarity, while TLP and MP's hydrophilic and hydrophobic residues appear in green and gray, respectively. Despite displaying a wide range of water defect values, the differences in membrane disruption caused by these MPs are difficult to visually discern.

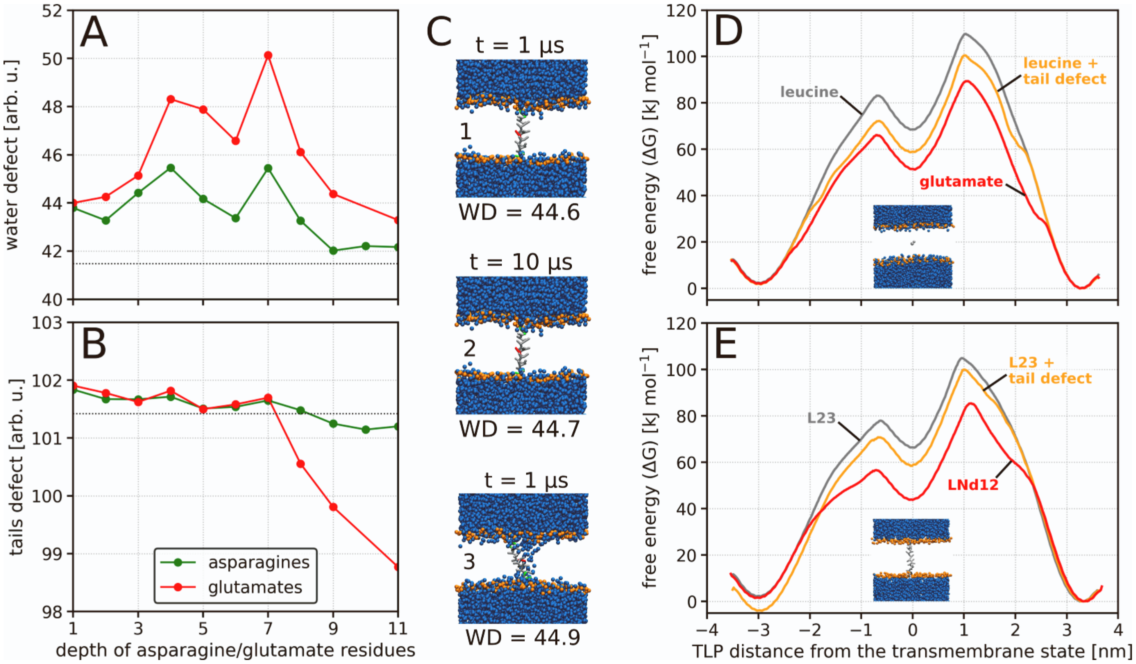

Figure S4: A. Water defects observed around symmetric leucine MPs with two asparagines (green line) or two glutamates (red line) positioned at various sequence depths (different membrane depths). Refer to Table S7 for sequences of these auxiliary MPs. The black dotted line represents the water defect in a pure membrane. The data point for glutamates at depth 10 is absent, as this specific MP (LE2d10) was unstable in the transmembrane state during simulation. B. Tail defects observed for the same MPs as in A. The black dotted line indicates the reference tail defect around MP L23, composed solely of leucines. Note that while MPs with "shallow" or "intermediate" depths of asparagines/glutamates exhibit higher tail defects than L23, those with centrally located asparagines/glutamates lead to a notable reduction in lipid tail density in the membrane center. C. To ensure the reduced water defect seen around MPs with centrally located hydrophilic/charged residues is not a result of initial conditions, we analyzed three simulations of MP SAGLE1 in a POPE:POPG (3:1) membrane: a standard 1 μs simulation (1), an extended 10 μs simulation (2), and a simulation where an artificial pore was introduced at the start of a 1 μs unbiased run (3). All simulations showed similar water defects (44.6 – 44.9 arb. u.). Note that the snapshots show *initial* configurations of the three simulated systems. In system 3, the artificially created pore quickly disappears and the system adopts configurations indistinguishable from systems 1 and 2. The initial pore in system 3 was created using an inverted cylindrical flat-bottomed potential applied to all lipid beads, centered on the membrane's center with a 1.4 nm reference distance in the xy plane and a 50 kJ mol^{-1} nm^{-2} force constant. D. Translocation profiles of the TLP LS9 in the presence of a single leucine or glutamate residue within the membrane. These amino acids were restrained to the membrane's local center of mass using a harmonic potential with a force constant of 5000 kJ mol^{-1} nm^{-2}. We also created an artificial tail defect near a leucine bead, reflecting the tail defect observed around a glutamate bead. This defect was achieved with an inverted spherical flat-bottomed potential applied to all lipid tail beads, centered on the leucine backbone bead with a 0.69 nm reference distance in xyz and a force constant of 500 kJ mol^{-1} nm^{-2}. While this disturbance did not impact the water defect or membrane thickness, the translocation barrier for TLP LS9 decreased in comparison to systems featuring leucine in a stable membrane. However, glutamate resulted in a more significant barrier reduction, indicating important enthalpic contributions and potentially other unidentified effects. E. Translocation profiles of the TLP LS9 with MP L23 or LNd12. We also created an artificial tail defect around L23, reflecting the tail defect observed around LNd12. The defect creation mirrored that in D, but with a 0.66 nm reference distance. There was a noticeable reduction in free energy caused by the artificial tail defect, yet this decrease was smaller than that caused by the charged MP LNd12. It is worth noting that replicating the tail defect around MPs is challenging, as the defect is primarily on one side of the MP, which our defect generation method does not account for.

Table S7: Names and sequences of auxiliary MPs for analysis of relationships between residue depth and water defect and between residue depth and tail defect. The number in the d* suffix denotes the residue depth as depicted in Figure S4 A, C. Although the data for LE2d10 were not utilized due to its instability in the transmembrane state, its sequence is provided for completeness.

Name	Sequence	Name	Sequence
LN2d1	NLLLLLLLLLLLLLLLLLLLLLN	LE2d1	ELLLLLLLLLLLLLLLLLLLLLE
LN2d2	LNLLLLLLLLLLLLLLLLLLLNL	LE2d2	LELLLLLLLLLLLLLLLLLLLEL
LN2d3	LLNLLLLLLLLLLLLLLLLLNLL	LE2d3	LLELLLLLLLLLLLLLLLLLELL
LN2d4	LLLNLLLLLLLLLLLLLLLNLLL	LE2d4	LLLELLLLLLLLLLLLLLLELLL
LN2d5	LLLLNLLLLLLLLLLLLLNLLLL	LE2d5	LLLLELLLLLLLLLLLLLELLLL
LN2d6	LLLLLNLLLLLLLLLLLNLLLLL	LE2d6	LLLLLELLLLLLLLLLLELLLLL
LN2d7	LLLLLLNLLLLLLLLLNLLLLLL	LE2d7	LLLLLLELLLLLLLLLELLLLLL
LN2d8	LLLLLLLNLLLLLLLNLLLLLLL	LE2d8	LLLLLLLELLLLLLLELLLLLLL
LN2d9	LLLLLLLLNLLLLLNLLLLLLLL	LE2d9	LLLLLLLLELLLLLELLLLLLLL
LN2d10	LLLLLLLLLNLLLNLLLLLLLLL	LE2d10	LLLLLLLLLELLLELLLLLLLLL
LN2d11	LLLLLLLLLLNLNLLLLLLLLLL	LE2d11	LLLLLLLLLLELELLLLLLLLLL

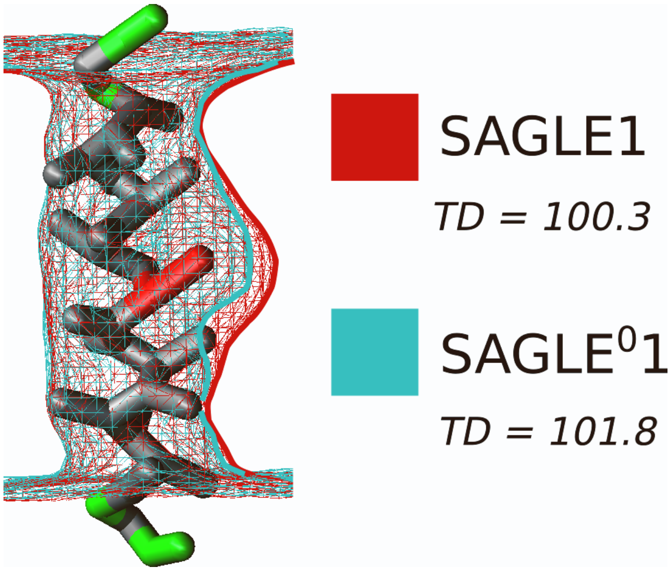

Figure S5: Isosurface representation of lipid tail densities calculated around the MPs SAGLE1 (dark red) and SAGLE^{0}1 (teal). The peptides differ only in the charge of the central glutamate residue, with SAGLE1's glutamate being charged and SAGLE^{0}1's being uncharged. As a result, the lipid density near SAGLE1 is lower ("tail defect" of 100.3 arb. u.) compared to that near SAGLE^{0}1 ("tail defect" of 101.8 arb. u.). This decrease in lipid tail density near SAGLE1 manifests as a pronounced protrusion in the density isosurface at the membrane's center. This density change is minor and not visible in individual simulation snapshots, but still affects peptide translocation.

Depth of hydrophilic residues

Table S8: Free energy differences for important points in the translocation profiles of TLP LS9 translocating in the presence of MPs with different depths of hydrophilic residues. Both translocation directions are shown separately. Free energy values are shown relative to the TLP in the adsorbed state [kJ mol^{-1}]. The error was estimated to be below 5 kJ mol^{-1}. Water defect (WD), upper leaflet water defect (uWD), lower leaflet water defect (lWD), tail defect (TD), upper leaflet tail defect (uTD), and lower leaflet tail defect (lTD), all in arb. u., are shown in the last six columns.

MP	direction	ΔG_{IC}	ΔG_{TM}	ΔG_{IN}	$\Delta\Delta G_{\mathrm{BM}}$	WD	uWD	lWD	TD	uTD	lTD
LNd3	N↓C↑	73	58	98	98	44.0	22.2	21.8	101.6	51.0	50.6
	N↑C↓	69	56	96	97						
LNd6	N↓C↑	68	50	91	91	46.0	23.2	22.8	101.6	50.9	50.7
	N↑C↓	68	55	92	92						
LNd9	N↓C↑	59	42	84	84	44.6	22.7	21.9	101.4	50.8	50.6
	N↑C↓	59	46	83	83						
LNd12	both	57	44	85	85	42.2	21.9	20.3	101.2	50.7	50.5

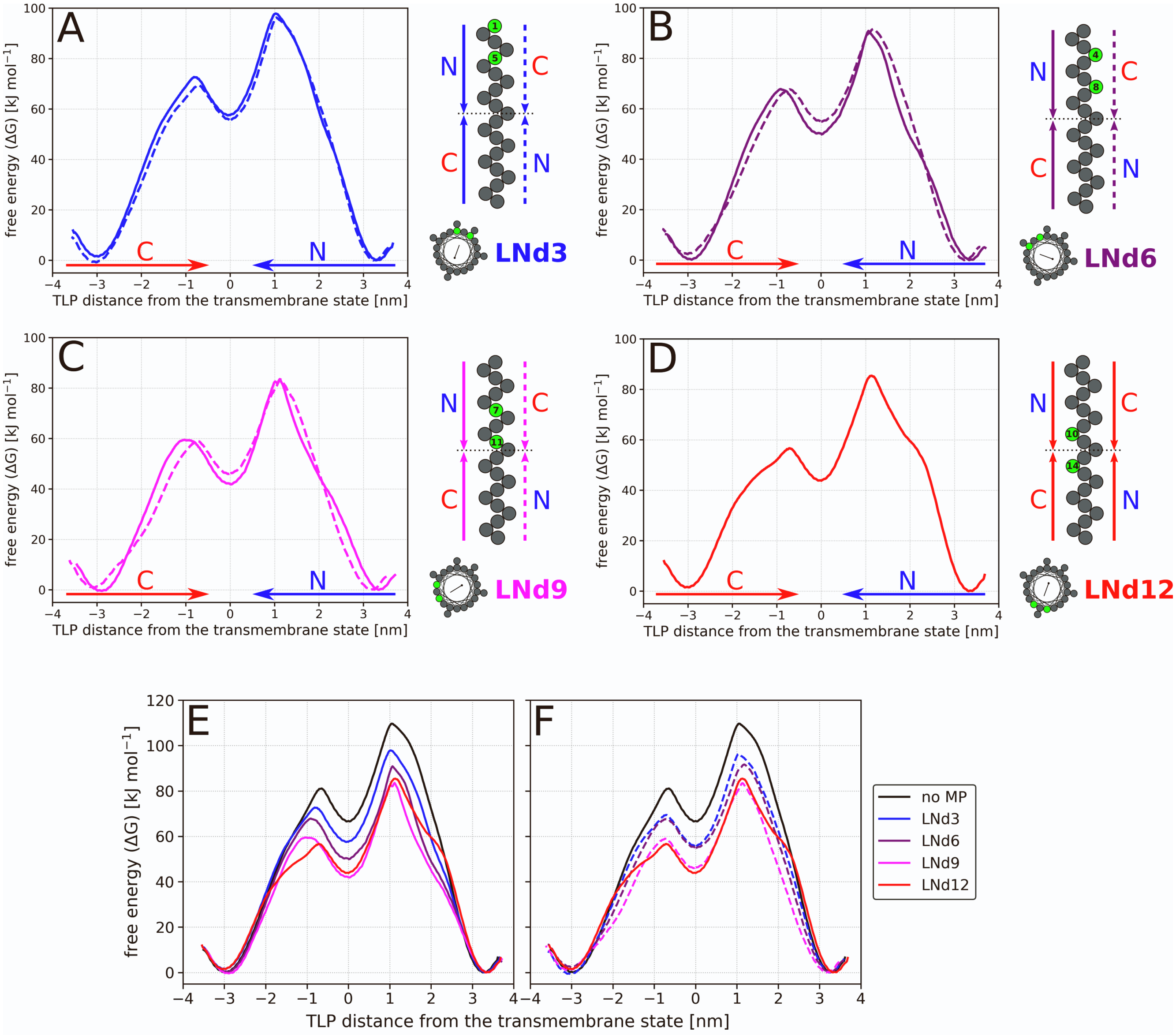

Figure S6: A—D. Translocation profiles of the TLP LS9 in the presence of MP LNd3 (A), LNd6 (B), LNd9 (C), and LNd12 (D). For asymmetric MPs (A–C), both translocation directions are presented. The N↓C↑ direction is represented with solid lines, and the N↑C↓ direction with dashed lines. The negative portion of each profile indicates C-terminus insertion, and the positive indicates N-terminus insertion. A schematic representation of the respective MP, highlighting the depth of its hydrophilic residues and the translocation directions, is positioned to the right of each chart. The MP's name and its helical wheel are displayed beneath the schematic. It is noteworthy that with asymmetric MPs (LNd3, LNd6, LNd9), the free energy profiles of the TLP for both translocation directions appear quite similar, potentially due to the apparent symmetrization of membrane disruption when these MPs are present. E—F. Translocation profiles of TLP LS9 traversing the membrane alone (in black) or along MP LNd3 (in blue), LNd6 (in purple), LNd9 (in pink), or LNd12 (in red). E outlines the translocation profiles for the N↓C↑ direction, while F depicts the N↑C↓ translocation direction. A deeper average position of the asparagines results in enhanced translocation.

Hydrophilic patch compactness

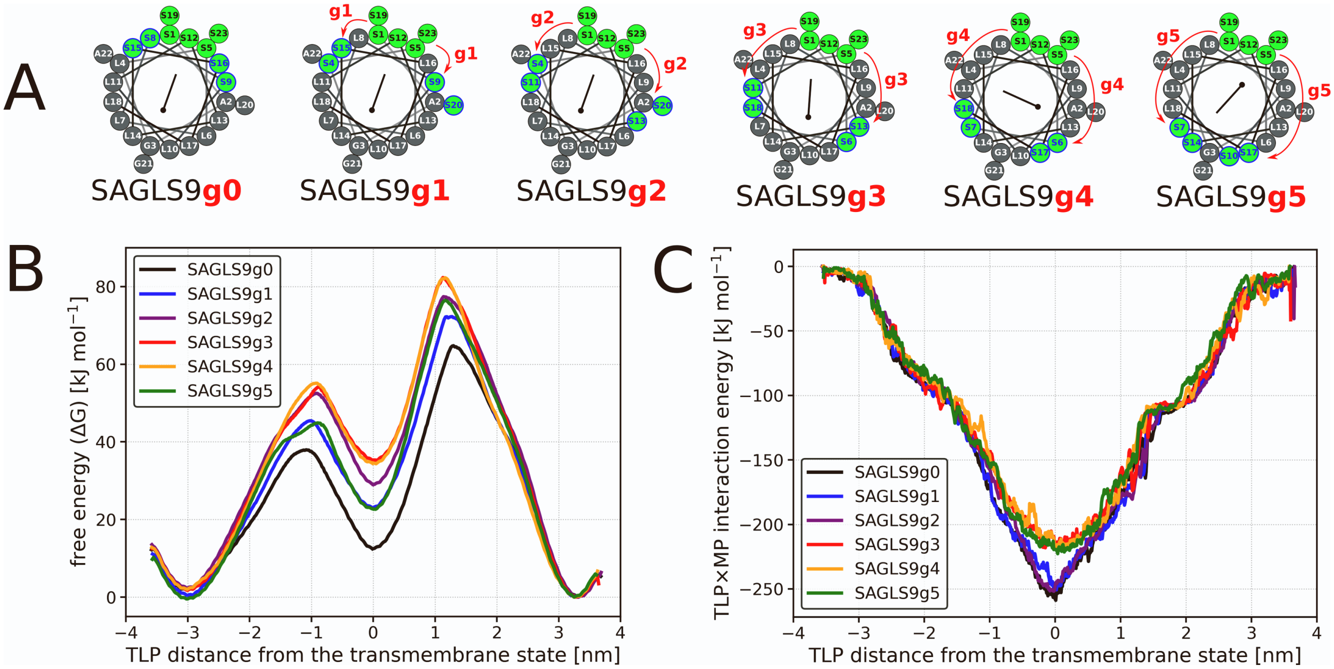

Figure S7: A. Helical wheel diagrams of the MPs SAGLS9g0–5. Each MP features a hydrophilic patch composed of serines 1, 5, 12, 19, and 23, with additional four serines (marked in blue) which positions vary. The g* suffix designates the number of hydrophobic residues in the helical wheel that separate the primary hydrophilic patch from the supplementary serines, termed a "gap". B. The translocation profiles of TLP LS9 as it moves through the membrane in the presence of MPs that have a disrupted hydrophilic patch, SAGLS9g0–5. Disrupting the hydrophilic patch of the MP notably diminishes the MP's ability to enhance translocation. C. The average strength of the total interaction (Lennard-Jones + coulombic) between TLP LS9 and MP SAGLS9g0–5. The interaction strength is computed from the simulated set of umbrella sampling windows and is represented as a function of the TLP's distance from its transmembrane state. It is worth noting that MPs with considerable disruptions to their hydrophilic patches (such as SAGLS9g3–5) offer significantly reduced enthalpic stabilization for the TLP when in a transmembrane state. This is due to the diminished count of hydrophilic residues in optimal positions that can interact with the TLP.

Table S9: Free energy differences for important points in the translocation profiles of TLP LS9 translocating in the presence of MPs with various hydrophilic patch compactness. Free energy values are shown relative to the TLP in the adsorbed state [kJ mol^{-1}]. The error was estimated to be below 5 kJ mol^{-1}. Water defect (WD) and tail defect (TD) [arb. u.] are shown in the last two columns of the table.

MP	ΔG_{IC}	ΔG_{TM}	ΔG_{IN}	$\Delta\Delta G_{\mathrm{BM}}$	WD	TD
SAGLS9g0	38	12	65	65	48.5	102.8
SAGLS9g1	45	23	72	72	48.4	103.0
SAGLS9g2	53	29	77	77	47.8	102.9
SAGLS9g3	54	35	82	82	48.5	102.9
SAGLS9g4	55	35	82	82	49.2	103.1
SAGLS9g5	45	23	77	77	47.9	102.8

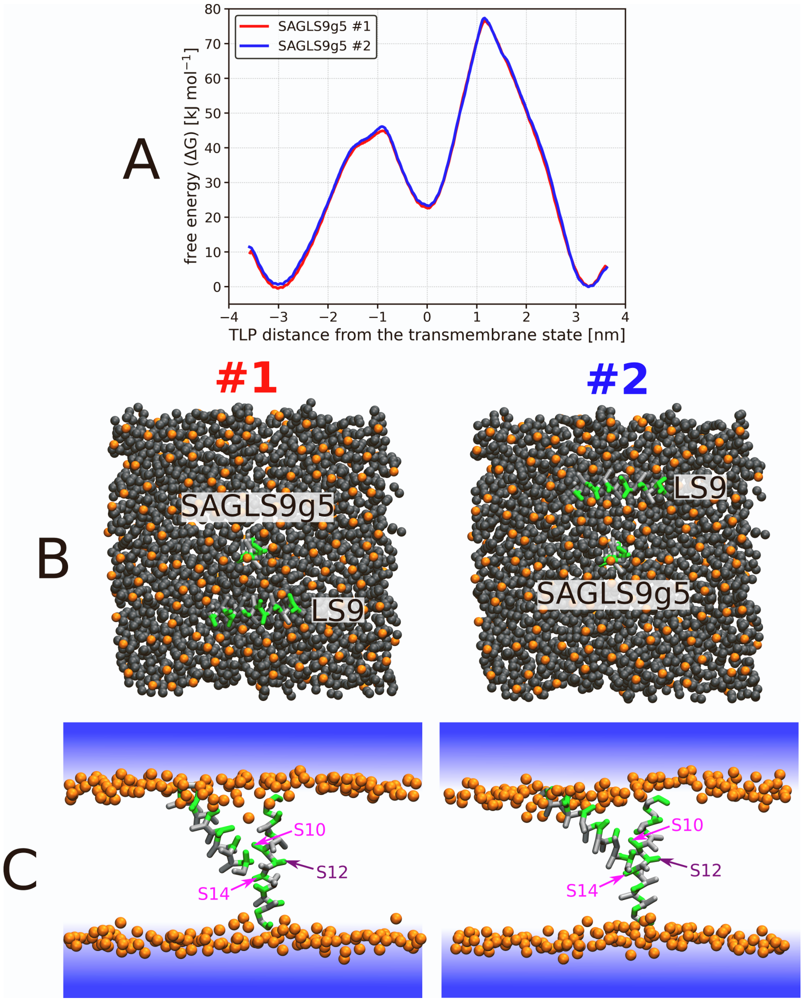

Figure S8: The ability of MP SAGLS9g5 to enhance translocation is not as diminished as we anticipated, considering the disruption of its hydrophilic patch. This phenomenon can be attributed to the four supplementary serines with varied positions that come very close to one another and generally point in the same direction (as illustrated in Figure S7 A). These additional serines create a secondary hydrophilic patch on the opposite side of the primary patch. Given that these specific serines (S7, S10, S14, and S17) are, on average, more centrally positioned within the membrane than those of the primary patch (S1, S5, S12, S19, S23), they offer greater stabilization for the TLP. As a result, the TLP tends to translocate along this secondary hydrophilic patch. To ensure that the TLP's preference for the secondary patch over the primary one is not merely an artifact of initial conditions, we performed two simulations of the TLP with SAGLS9g5, each with a different initial position of the TLP in its adsorbed state (B). The translocation profiles calculated from these two systems with differing starting conditions are nearly indistinguishable (A). In both simulations, the TLP selects the same translocation pathway (C). For all subsequent analyses, we have chosen to use the free energy profile from system #1 as the standard translocation profile for TLP LS9 when in the presence of SAGLS9g5.

Presence of charged residues

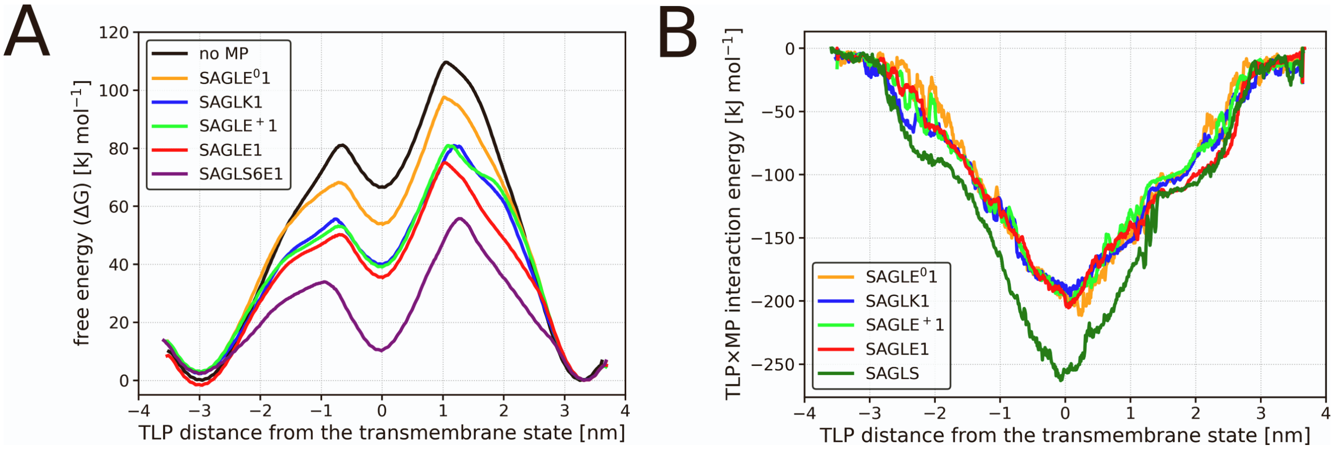

Figure S9: A. Translocation profiles of the TLP LS9 moving through the membrane alone or in the presence of MP SAGLE1, SAGLK1, SAGLE^{0}1, SAGLE$^+$1, or SAGLS6E1. Even a single charged residue in the MP reduces the translocation barrier, but its effect on the TLP's stability in the transmembrane state is less pronounced. Incorporating a charged residue into an MP with a hydrophilic patch can also enhance translocation. B. Average strength of the total interaction (Lennard-Jones + coulombic) between the TLP LS9 and the MP SAGLK1, SAGLE^{0}1, SAGLE$^+$1, SAGLE1, or SAGLS, calculated from simulated umbrella sampling windows and represented as a function of the TLP's distance from its transmembrane state. Note that the interaction between the TLP and the MP SAGLS is notably stronger than interaction between the TLP and the other MPs, especially in the transmembrane state of the TLP. This results in decreased stability of the TLP in its transmembrane state during translocation along MPs like SAGLK1, SAGLE$^+$1, SAGLE^{0}1, or SAGLE1, compared to the MP SAGLS.

Table S10: Free energy differences for important points in the translocation profiles of TLP LS9 translocating in the presence of MPs with charged residues or with a protonated glutamate. Free energy values are shown relative to the TLP in the adsorbed state [kJ mol^{-1}]. The error was estimated to be below 5 kJ mol^{-1}. Water defect (WD) and tail defect (TD) [arb. u.] are shown in the last two columns of the table.

MP	ΔG_{IC}	ΔG_{TM}	ΔG_{IN}	$\Delta\Delta G_{\mathrm{BM}}$	WD	TD
SAGLE1	50	35	75	77	44.6	100.3
SAGLE^{0}1	68	54	98	98	44.4	101.8
SAGLE$^+$1	53	39	81	81	44.9	100.3
SAGLK1	55	40	81	81	45.3	100.8
SAGLS6E1	34	10	56	56	47.8	101.3

Type of hydrophobic residues

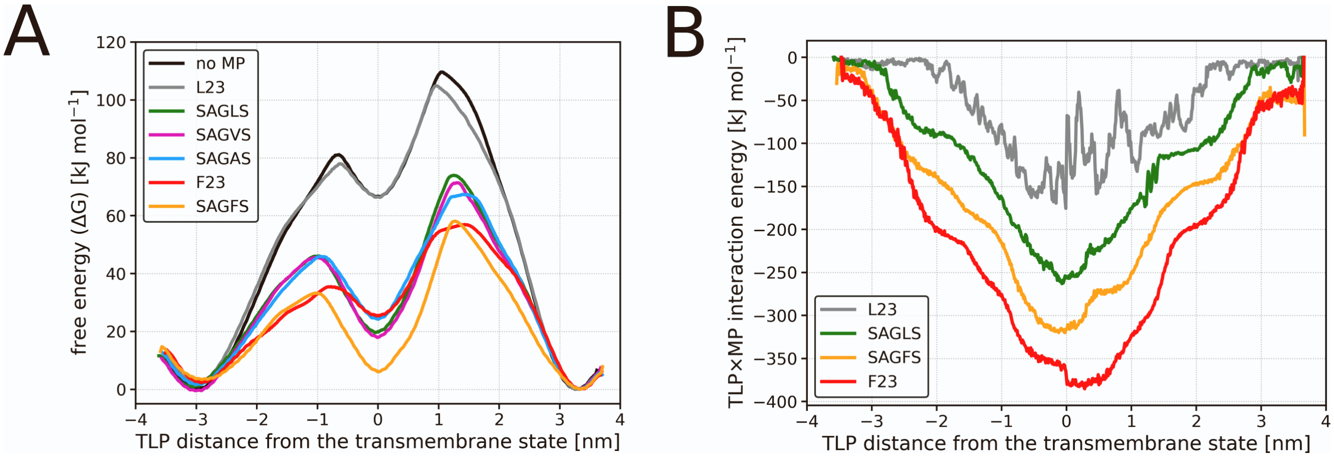

Figure S10: A. Translocation profiles of the TLP LS9 moving through the membrane alone or in the presence of MPs L23, SAGLS, SAGVS, SAGAS, F23, or SAGFS. The character of the hydrophobic residues composing the MP can in many cases significantly affect the translocation-enhancing abilities of the MP. B. Average strength of the total interaction (Lennard-Jones + coulombic) between the TLP LS9 and MP L23, SAGLS, SAGFS, or F23 calculated from the simulated set of umbrella sampling windows and shown as a function of the TLP distance from the transmembrane state. Observe the difference in enthalpic stabilization between MPs L23 and F23, as well as between SAGLS and SAGFS.

Table S11: Free energy differences for important points in the translocation profiles of TLP LS9 translocating in the presence of MPs containing different hydrophobic residues. Free energy values are shown relative to the TLP in the adsorbed state [kJ mol^{-1}]. The error was estimated to be below 5 kJ mol^{-1}. Water defect (WD) and tail defect (TD) [arb. u.] are shown in the last two columns of the table. Note that the values of the tail defect are heavily biased for this set of MPs due to the different sizes of the sidechains of the hydrophobic residues.

MP	ΔG_{IC}	ΔG_{TM}	ΔG_{IN}	$\Delta\Delta G_{\mathrm{BM}}$	WD	TD
SAGLS	46	20	74	74	47.3	102.6
SAGVS	46	18	71	72	47.2	103.3
SAGAS	41	18	66	67	47.3	106.5
SAGFS	33	6	58	58	52.8	100.0
F23	35	25	57	57	45.9	97.3
L23	78	66	105	105	41.6	101.4

Relationship between $\Delta\Delta G_{\mathbf{BM}}$ and $\Delta G_{\mathbf{TM}}$

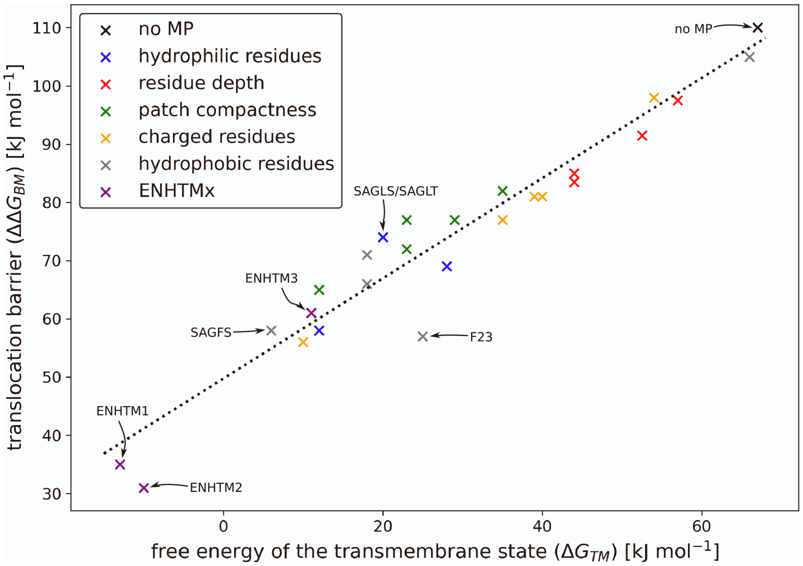

Figure S11: The relationship between the translocation barrier, $\Delta\Delta G_{\mathrm{BM}}$, and the free energy of the transmembrane state, ΔG_{TM}, calculated for all simulated systems. Individual points represent the systems, with colors indicating the "type" of MP present. Several MPs of interest are labeled. The relationship between these properties is largely linear, as evidenced by a correlation coefficient of 0.96.

Translocation across POPC membranes

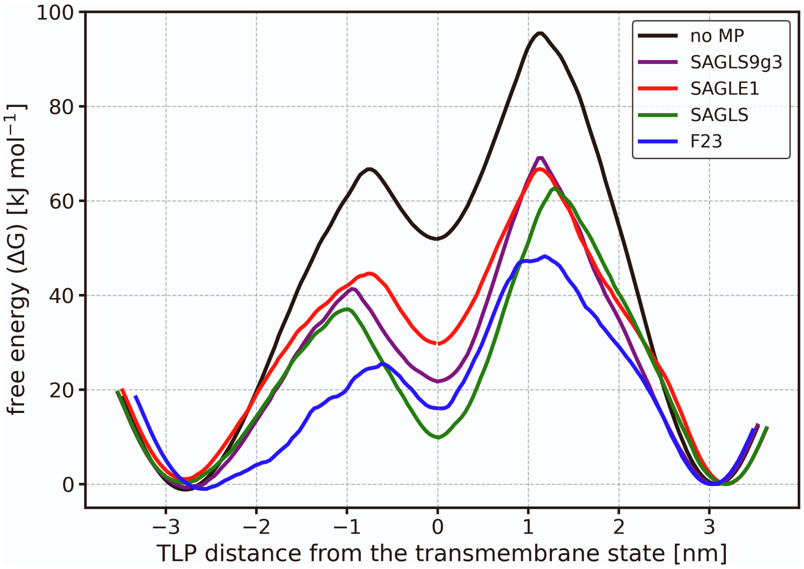

Figure S12: Translocation profiles of the TLP LS9 moving through a POPC membrane alone or in the presence of various MPs (SAGLS9g3, SAGLE1, SAGLS, F23). The translocation barriers across the POPC membrane are generally lower than those across the POPE:POPG (3:1) membrane, but MPs continue to exhibit their translocation-enhancing abilities.

Table S12: Free energy differences for important points in the translocation profiles of TLP LS9 translocating through pure POPC membrane or through POPC membrane in the presence of various MPs. Free energy values are shown relative to the TLP in the adsorbed state [kJ mol^{-1}]. The error was estimated to be below 5 kJ mol^{-1}. Water defect (WD) and tail defect (TD) [arb. u.] are shown in the last two columns of the table.

MP	ΔG_{IC}	ΔG_{TM}	ΔG_{IN}	$\Delta\Delta G_{\mathrm{BM}}$	WD	TD
no MP	67	52	95	97	64.0	N/A
SAGLS9g3	41	22	69	70	72.0	102.9
SAGLE1	45	30	67	67	66.0	100.3
SAGLS	37	10	63	63	69.6	102.6
F23	25	16	48	49	66.4	97.1
