# Supplementary material for: Optimizing properties of translocation-enhancing transmembrane proteins
Source: Biophys J. 2024 Apr 13;123(10):1240–52. doi: 10.1016/j.bpj.2024.04.009 (PMC11140465; doi:10.1016/j.bpj.2024.04.009)
Supplement: Document S2. Article plus Supporting Material [file mmc2.pdf]

Biophysical Journal
Article

Optimizing properties of translocation-enhancing transmembrane proteins

Ladislav Bartoš,[1,2] Martina Drabinová,[1] and Robert Vácha[1,2,3,*]
[1]CEITEC – Central European Institute of Technology, Masaryk University, Brno, Czech Republic; [2]National Centre for Biomolecular Research, Faculty of Science, Masaryk University, Brno, Czech Republic; and [3]Department of Condensed Matter Physics, Faculty of Science, Masaryk University, Brno, Czech Republic

ABSTRACT Cell membranes act as semi-permeable barriers, often restricting the entry of large or hydrophilic molecules. Nonetheless, certain amphiphilic molecules, such as antimicrobial and cell-penetrating peptides, can cross these barriers. In this study, we demonstrate that specific properties of transmembrane proteins/peptides can enhance membrane permeation of amphiphilic peptides. Using coarse-grained molecular dynamics with free-energy calculations, we identify key translocation-enhancing attributes of transmembrane proteins/peptides: a continuous hydrophilic patch, charged residues preferably in the membrane center, and aromatic hydrophobic residues. By employing both coarse-grained and atomistic simulations, complemented by experimental validation, we show that these properties not only enhance peptide translocation but also speed up lipid flip-flop. The enhanced flip-flop reinforces the idea that proteins such as scramblases and insertases not only share structural features but also operate through identical biophysical mechanisms enhancing the insertion and translocation of amphiphilic molecules. Our insights offer guidelines for the designing of translocation-enhancing proteins/peptides that could be used in medical and biotechnological applications.

SIGNIFICANCE Cells are enveloped by a selectively permeable cytoplasmic membrane. Certain peptides can spontaneously penetrate this protective barrier, serving as potential drug carriers or therapeutic agents. In this study, we demonstrate that the translocation of peptides across membrane into cell can be enhanced with the passive assistance of transmembrane proteins. We investigate various properties of these transmembrane proteins that enhance the translocation and we establish guidelines for designing such translocation enhancers. We show that these enhancers also facilitate lipid scrambling, which suggests more general effect on amphiphilic molecules. Therefore, proteins facilitating the insertion of amphiphilic molecules into the membrane's hydrophobic core, such as translocation enhancers, insertases, translocases, and scramblases, might share a common biophysical mechanism.

INTRODUCTION

Living cells are enclosed by a thin phospholipid bilayer that controls the exchange of matter between the cell and its environment. Typically, only small and uncharged molecules can permeate the cell membrane directly, whereas the transport of other molecules requires specific, tightly regulated channels and transporters. Nonetheless, some larger amphiphilic molecules, including peptides, have been observed to spontaneously cross the cell membrane. Notably, antimicrobial peptides (AMPs) and cell-penetrating peptides (CPPs) stand out as potential therapeutic agents and drug carriers, respectively (1,2).

As research into the characteristics of AMPs and CPPs required for membrane translocation continues (3,4), it becomes clear that proteins within phospholipid/cellular membranes affect the local membrane properties, which could enable other molecules to enter the cell. Our prior study (5) demonstrated that transmembrane proteins or peptides (MPs) with a continuous hydrophilic region significantly enhance the translocation of amphiphilic peptides (TLPs) across the membrane. These MPs, with their hydrophilic patches, locally disrupt the membrane structure, allowing TLPs to more easily cross the membrane's hydrophobic core. Furthermore, MPs provide enthalpic stabilization of the TLP via their hydrophilic residues. Interestingly, the presence of a hydrophilic patch spanning the membrane

Submitted January 7, 2024, and accepted for publication April 9, 2024.
*Correspondence: robert.vacha@muni.cz
Editor: Rainer Boeckmann.
https://doi.org/10.1016/j.bpj.2024.04.009
© 2024 Biophysical Society.
This is an open access article under the CC BY license (http://creativecommons.org/licenses/by/4.0/).

has also been observed in proteins known as scramblases, which facilitate lipid flip-flop, the translocation of phospholipids between the membrane's leaflets (6–10).

In this study, we used coarse-grained simulations to identify properties that maximize translocation enhancement beyond the size of the hydrophilic patch (see Fig. 1). Building on our earlier findings (5), we offer an extensive guide for designing proteins/peptides that enhance the translocation of α-helical amphiphilic peptides, such as uncharged AMPs or CPPs. Furthermore, through both coarse-grained and atomistic simulations as well as using experimental approaches, we demonstrate that the properties of MPs promoting peptide translocation can also accelerate lipid flip-flop.

MATERIALS AND METHODS

Peptide translocation simulations

All of our simulations on peptide translocation were conducted using the Gromacs package versions 5.1.4 and 2021.4 (11). We utilized the coarse-grained force field, Martini version 2.2 (12–14). Lennard-Jones interactions between peptide and protein beads were downscaled by 10% to prevent overly strong protein-protein interactions (15). We refrained from using the Martini 3 force field because it fails to accurately represent a critical property for our simulations: the stability of transmembrane proteins in the membrane (16). Furthermore, this stability is closely tied to the membrane disruption caused by the MPs and probably arises from the excessively high hydrophilicities of the protein beads.

Systems preparation

Most systems consisted of a bacterial-membrane mimic composed of 256 lipids: 96 molecules of 1-palmitoyl-2-oleoyl-*sn*-glycero-3-phosphocholine (POPE) and 32 molecules of 1-palmitoyl-2-oleoyl-*sn*-glycero-3-phosphoglycerol (POPG) in each leaflet. We also prepared several additional systems with a membrane composed of 128 molecules of 1-palmitoyl-2-oleoyl-*sn*-glycero-3-phosphocholine (POPC) in each leaflet (256 lipids in total). Bilayers were was prepared using the CHARMM-GUI web interface (17). Each system had an approximate size of $9 \times 9 \times 11$ nm and was solvated with roughly 4400 coarse-grained water beads.

Peptides and proteins were modeled using MODELLER versions 9.11 and 10.4 (18) and subsequently coarse-grained with the martinize.py script version 2.6 (https://github.com/cgmartini/martinize.py). We then minimized the potential energy of each peptide/protein in a vacuum environment using the steepest descent algorithm, setting a maximum force tolerance of 100 kJ mol^{-1} nm^{-1}. All the peptides/proteins were generated with an α-helical conformation and were restrained to maintain this conformation throughout the simulation. We utilized the HeliQuest ComputParams.py script version 3 (https://heliquest.ipmc.cnrs.fr/cgi-bin/ComputParams.py) to calculate the hydrophobicity of each peptide/protein. The hydrophobicity scale is based on the partitioning of *N*-acetyl-amino-acid amides in octanol (19).

Translocating peptide (TLP) was subsequently placed on the membrane surface, oriented parallel to the membrane plane with its hydrophilic patch directed toward the solvent. The specific TLP sequence utilized was named LS9 (LSSLLSLLSSLLSLLSSLLSL-NH2) with a hydrophobicity value of 0.954. The transmembrane protein/peptide (MP) was centrally placed in the membrane, oriented perpendicular to the membrane plane. The sequences and hydrophobicities (H) of all MPs used can be found in Table 1. Note that the MPs SAGLE1, $SAGLE^{+}1$, and $SAGLE^{0}1$, have variations in the charge of their glutamate residue. Specifically, SAGLE1 possesses a negatively charged glutamate, $SAGLE^{+}1$ has an artificial positively charged glutamate, and $SAGLE^{0}1$ features a protonated (uncharged) glutamate. Additionally, we carried out simulations of TLP LS9 translocation without the presence of an MP as a control.

After the addition of the TLP and MP, the system was ionized using Na^{+} and Cl^{-} ions at a physiological concentration of 154 mM, with an excess of ions to ensure system neutrality. Subsequently, each system underwent energy minimization employing the steepest descent algorithm, with a maximum force tolerance set at 100 kJ mol^{-1} nm^{-1}.

Equilibration

Each system underwent equilibration in five stages with varying simulation time steps and overall durations: 1) dt = 2 fs and t = 0.5 ns, 2) dt = 5 fs and t = 1.25 ns, 3) dt = 10 fs and t = 1 ns, 4) dt = 20 fs and t = 30 ns, and 5) dt = 20 fs and t = 15 ns. Throughout stages 1–4, all backbone bead coordinates of both MP and TLP were restrained using a harmonic potential with a force constant of 1000 kJ mol^{-1} nm^{-2}. In stage V of the equilibration and in subsequent molecular dynamics, pulling, and umbrella sampling simulations, a flat-bottomed potential ensured that the TLP remained in proximity to the MP. This potential was set between the centers of mass of the TLP and MP backbone beads with a reference distance of 2.5 nm in the xy plane. The applied force constant for this potential was 500 kJ mol^{-1} nm^{-2}.

Each equilibration stage was performed in an NPT ensemble, maintaining a temperature of 310 K using the stochastic velocity rescaling thermostat (20) with a coupling constant set at 1 ps. The water and ions, as well as the membrane and peptides/proteins, were coupled separately. Pressure was kept at 1 bar using the Berendsen barostat (21) with a 12-ps coupling constant. A semi-isotropic pressure coupling scheme was used to scale the simulation box independently in the xy plane and along the z axis, with a compressibility of 3×10^{-4} bar^{-1}. The Newtonian equations of motion were integrated using the leap-frog algorithm. Nonbonded interactions were cut off at 1.1 nm, and the van der Waals potential was shifted to zero at the cutoff distance. The relative dielectric constant was set at 15.

After the equilibration, a brief 100-ns molecular dynamics simulation was conducted. For this simulation, and all subsequent ones, the Berendsen thermostat was replaced with the Parrinello-Rahman barostat (22,23). All other simulation parameters were consistent with those from stage 5 of the equilibration.

Free-energy calculations

We enhanced the sampling of the configuration space using the umbrella sampling method (24,25). For the symmetric MPs (all MPs expect for

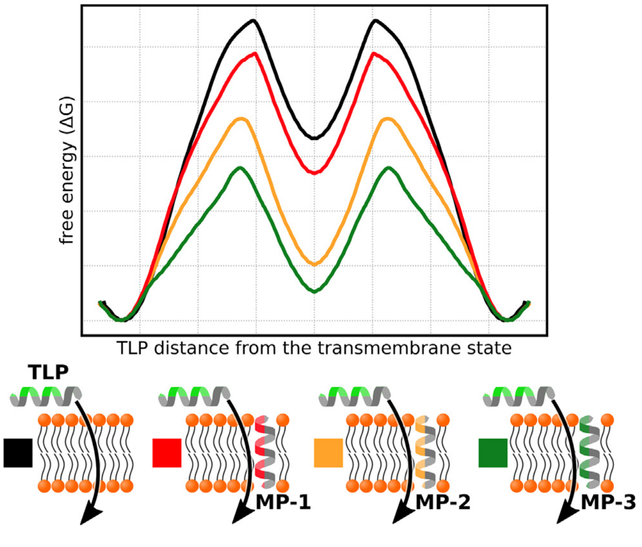

FIGURE 1 Aim of the study demonstrated on the translocation free-energy profiles, defined along the distance between the TLP and the membrane's center of mass. MPs with different properties (illustrated by red, orange, and green patches) have different translocation-enhancing abilities. In this study, we searched for an optimal translocation enhancer: an MP that allows for the easiest passage of the TLP across the membrane. To see this figure in color, go online.

TABLE 1 Names, Sequences, and Hydrophobicities of MPs Used in the Study

Name	Sequence	H
SAGLS	SAGLSLLSLLLSLLLSLLSLGAS	0.902
SAGLT	SAGLTLLTLLLTLLLTLLTLGAS	0.967
SAGLQ	SAGLQLLQLLLQLLLQLLQLGAS	0.863
SAGLN	SAGLNLLNLLLNLLLNLLNLGAS	0.780
LNd3	NLLLNLLLLLLLLLLLLLLLLLL	1.500
LNd6	LLLNLLLNLLLLLLLLLLLLLLL	1.500
LNd9	LLLLLLNLLLNLLLLLLLLLLLL	1.500
LNd12	LLLLLLLLLNLLLNLLLLLLLLL	1.500
SAGLS9g0	SAGLSLLSSLLSLLSSLLSLGAS	0.750
SAGLS9g1	SAGSSLLLSLLSLLSLLLSSGAS	0.750
SAGLS9g2	SAGSSLLLLLSSSLLLLLSSGAS	0.750
SAGLS9g3	SAGLSSLLLLSSSLLLLSSLGAS	0.750
SAGLS9g4	SAGLSSSLLLLSLLLLSSSLGAS	0.750
SAGLS9g5	SAGLSLSLLSLSLSLLSLSLGAS	0.750
SAGLE1	SAGLLLLLLLLELLLLLLLLLGAS	1.178
SAGLE^{+}1	SAGLLLLLLLLELLLLLLLLLGAS	1.178
SAGLE^{0}1	SAGLLLLLLLLELLLLLLLLLGAS	1.178
SAGLK1	SAGLLLLLLLLKLLLLLLLLLGAS	1.163
SAGLS6E1	SAGLSLLSLLLELLLSLLSLGAS	0.876
F23	FFFFFFFFFFFFFFFFFFFFFFF	1.790
L23	LLLLLLLLLLLLLLLLLLLLLLL	1.700
SAGFS	SAGFSFFSFFFSFFFSFFSFGFS	1.013
SAGVS	SAGVSVVSVVVSVVVSVVSVGAS	0.651
SAGAS	SAGASAASAAASAAASAASAGAS	0.177
ENHTM1	NFFNNFFNNFFEFFNNFFNNFFN	0.645
ENHTM2	NGWFNFFNEFFEFFENFFNFWGN	0.734
ENHTM3	SLLSSLLSSLLKLLSSLLSSLLS	0.827
KLL	KKLLLLLLLLLLLLLLLLLLLLLLLLLLKK	1.301
9Q3H	Ac-WKKLALALQLAHALALALALALKK-NH2	0.666

H, hydrophobicities.

LNd3, LNd6, and LNd9), we adopted the approach from our prior studies (5,26). Specifically, the TLP translocation process was split into two distinct steps: C terminus insertion and N terminus insertion. We excluded the TLP adsorption/desorption phase, as it previously remained unaffected by the presence of MPs (5). Given that both the membrane and the MP were symmetric, the free-energy profile for TLP's translocation across the membrane was obtained by joining the free-energy profiles from each insertion process.

For the asymmetric MPs (LNd3, LNd6, LNd9), the translocation process was calculated separately for each possible "direction" of translocation. Given the symmetry of the membrane, we analyzed the insertion of each terminus of the TLP in two different orientations of the MP: once with the MP aligned at 180° on the z axis and once at 0° on the z axis within the membrane. This yielded four distinct insertion profiles labeled as N ↓, N ↑, C ↓, and C ↑. Here, the down arrow (↓) indicates the TLP's insertion from the upper leaflet to the lower leaflet, and the up arrow (↑) signifies the reverse insertion direction. By pairing the relevant profiles (N ↓ with C ↑ and N ↑ with C ↓), we derived two full translocation profiles, each representing a specific translocation direction.

For each insertion process, we used a collective variable (CV) defined as the oriented distance between the terminus center of mass and the local membrane center of mass along the z axis. The terminus refers to either the first (N) or the last (C) three backbone beads of the TLP. The local membrane center of mass was determined using the lipid beads situated within a cylinder that had a radius of 2.0 nm, with its principal axis passing through the TLP terminus along the z axis.

In most of the simulated systems, the starting configurations for umbrella sampling simulations were obtained by pulling a single TLP terminus through the membrane. This terminus underwent pulling for 1 μs at a rate of 4.2 nm μs^{-1}, starting from an initial reference distance of 2.1 nm. A harmonic potential, having a force constant of 8000 kJ mol^{-1} nm^{-2}, was applied during this pulling simulation.

The trajectory from the pulling simulation was split into 64 nonuniformly spaced sampling windows, as detailed in Table S1 (for systems with POPE:POPG membrane) and Table S2 (for systems with POPC membrane). After a brief 30-ns equilibration, each window was subjected to sampling for at least 1 μs.

For certain systems, specifically those with MPs F23, SAGFS, and SAGAS, sampling was further refined using Hamiltonian replica exchange (27) with an exchange attempt made every 5000 integration steps (100 ps) as implemented in the Plumed plugin version 2.3 (28). To ensure diverse system configurations, a "backward-pulling" simulation was performed for each TLP terminus. At the start of each backward pulling, the TLP was placed in a transmembrane state, and the respective terminus was pulled out of the membrane. Initial configurations for the umbrella sampling windows were taken from both forward- and backward-pulling simulations, with the origin of the windows alternating within the designated range of the CV.

The free-energy profile for each N or C terminus insertion was derived from the simulated umbrella sampling windows. This was done using the weighted histogram analysis method (29,30) as implemented in the tool g_wham (31).

Calculations of membrane disruption

To determine the impact of MP presence on the membrane's structure, we simulated one additional system for each MP. In these simulations, a single MP was introduced into the membrane without any TLP. The system was prepared, minimized, and equilibrated following the above-mentioned procedures. The production molecular dynamics phase was 1 μs, from which we computed 1) the local water defect (WD), i.e., the average count of water beads within 2.5 nm from the MP in the xy plane and within 2 nm from the membrane's global geometric center on the z axis; and 2) the tail defect (TD), the average count of lipid tail beads within 2.0 nm from the MP in the xy plane and within 0.5 nm from the membrane's geometric center on the z axis. Additionally, we characterized other defect-related metrics: 1) the upper leaflet water/TD, which exclusively counts water/tail beads positioned above the membrane's center; and 2) the lower leaflet water/TD, which exclusively counts water/tail beads situated below the membrane's center. The in-house-developed code used for the calculation of water and TD is available from https://github.com/Ladme/memdian.

Lipid-scrambling simulations

All of our lipid-scrambling simulations were performed using Gromacs package versions 2021.4 and 2023-dev (11). Two force fields were employed for these simulations: 1) the coarse-grained Martini force field version 2.2 (12–14) and 2) the atomistic Amber 99SB-ILDN force field for describing the peptides/proteins combined with the atomistic Slipids 2020 force field for the lipids (32–35).

Martini simulations

Our Martini simulations of lipid scrambling followed a procedure akin to that of the peptide translocation simulations. We constructed a membrane comprising 288 POPC lipids (with 144 lipids in each leaflet) using the CHARMM-GUI web interface (17). This system spanned an approximate size of 10 × 10 × 12 nm and was solvated with roughly 6000 coarse-grained water beads. A transmembrane protein (MP), prepared as outlined in the peptide translocation simulations section, was centrally positioned within the membrane, oriented perpendicular to the membrane plane. Subsequent steps of minimization and equilibration were the same as for translocation simulations.

A lipid positioned close to the MP was selected and its phosphate bead was pulled through the membrane over a duration of 1 μs. The pulling rate was set at 4.6 μs^{-1} with an initial reference distance of 2.3 nm. The pulling was achieved using a harmonic potential with a force constant of 5000 kJ mol^{-1} nm^{-2}. Our

chosen CV was the oriented distance between the lipid's phosphate and the local membrane's center of mass along the z axis. This local membrane center of mass was calculated using the same criteria detailed in the peptide translocation simulations section. We restrained the pulled phosphate to the MP in the xy plane using a flat-bottomed potential, with a force constant of 500 kJ mol^{-1} nm^{-2} and a reference distance of 1.5 nm from the MP's center of mass. In contrast to the peptide translocation simulations, the MP was fixed at the membrane's center by employing a harmonic potential with a force constant of 100 kJ mol^{-1} nm^{-2}. This maintained a reference distance of 0.0 nm from the local membrane center of mass along the z axis. The same protocol was used for MP free membrane, but without MP restraints.

Subsequently, the pulling trajectory was split into 67 nonuniform umbrella sampling windows (see Table S3). Each window was simulated for either 1 or 8 μs (for POPC and POPC with ENHTM3, respectively). The initial 10 ns of these simulations were used for equilibration. The calculation of the free-energy profiles followed the procedure outlined in the "peptide translocation simulations" section, employing the weighted histogram analysis method (29,30).

Atomistic simulations

In the atomistic simulations, we utilized a pre-equilibrated POPC membrane obtained from the Slipids website (http://www.fos.su.se/~sasha/SLipids/Downloads_files/POPC_303K.gro). This starting membrane consisted of 128 POPC lipids (divided evenly with 64 lipids in each membrane leaflet) accompanied by approximately 5100 water molecules. We introduced NaCl ions into the system to achieve a concentration of 0.154 mol dm^{-3}. After this, a small pore was created within the membrane during a 200-ps molecular dynamics simulation. This was accomplished with an inverted flat-bottomed potential, which had a force constant of 50 kJ mol^{-1} nm^{-2} and a reference distance of 1.1 nm from the box center. This potential was applied to the heavy atoms of all POPC lipids. After creating the pore, the MP (constructed using Avogadro version 1.2 (36)) was positioned within it. The membrane, with the embedded MP, was then energy minimized with a force tolerance of 1000 kJ mol^{-1} nm^{-1} and underwent equilibration. During this equilibration phase, the pore quickly sealed itself around the MP. For subsequent lipid-scrambling simulations where the MP was absent, we utilized a POPC membrane that did not have a pore at any point during the process.

The equilibration process for the membrane containing the MP spanned five distinct stages. In stage I, which was 2 ns long, position restraints, with a force constant of 1000 kJ mol^{-1} nm^{-2}, were applied to all heavy protein atoms and to the phosphorus atoms of the lipids. During stage II, the position restraints previously applied to the lipids were removed, and the simulation ran for an additional 5 ns. In stage III, position restraints were placed only on the backbone atoms of the MP, which ran for 5 ns. In the subsequent stage IV, these restraints were confined only to the C_α atoms of the MP and the simulation ran for 5 ns. Finally, in stage V, no position restraints were enforced, and this final stage continued for 10 ns. All stages used a simulation time step of 2 fs. We employed the stochastic velocity rescaling thermostat (20) with a coupling constant of 0.5 ps to ensure a consistent temperature of 310 K. Separate thermal baths were designated for the water with ions and for the membrane with the MP. The pressure was set at 1 bar using the Berendsen barostat (21) with semi-isotropic pressure coupling, a coupling time of 2 ps, and a compressibility factor of 4.5×10^{-5} bar^{-1}. The LINCS (37) method was used to constrain all bonds. Short-ranged, nonbonded interactions had a cutoff at 1.2 nm, whereas long-range electrostatic interactions employed the fast smooth particle-mesh Ewald method (38). The removal of translational velocity was performed separately for the membrane containing the MP and the water with ions.

After the equilibration, both the pure POPC and the POPC + MP membranes underwent 100 ns of molecular dynamics simulations with production parameters. At this stage, and in all subsequent simulations, we replaced the Berendsen barostat with the Parrinello-Rahman barostat (22,23). All other simulation settings remained consistent with those from stage V of equilibration. It is important to note that we did not artificially maintain the α-helical conformation of the MP.

Next, we selected a lipid located near the MP. We pulled this lipid from the upper leaflet to the lower leaflet by its phosphorus atom. This pulling lasted for 500 ns, with a pulling rate of 8.4 μs^{-1} and an initial reference distance of 2.1 nm. We employed a harmonic potential with a force constant of 5000 kJ mol^{-1} nm^{-2}. The CV mirrored the one used in the Martini 2 simulations of lipid scrambling; however, we used a phosphorus atom instead of a phosphate bead. In scenarios where an MP was present, we restrained the phosphorus to the MP in the xy plane using a flat-bottomed potential with a force constant of 500 kJ mol^{-1} nm^{-2} and a reference distance of 1.5 nm. For systems that included the MP, we also conducted pulling in the reverse direction—from the lower to the upper leaflet. In such cases, the initial reference distance was set to -2.1 nm, and the pulling direction was reversed, although all other simulation parameters remained unchanged.

The pulling trajectory obtained for the pure POPC system was then split into 59 nonuniformly distributed umbrella sampling windows (see Table S4 for details). To augment the sampling, we used Hamiltonian replica exchange (27) as implemented in the Plumed plugin version 2.7.2 (28) to 16 windows situated near the center of the membrane. Configuration exchanges were attempted every 100,000 integration steps (200 ps). Each window underwent simulation for 800 ns, with the initial 50 ns designated solely for equilibration. We calculated the free energy utilizing the weighted histogram analysis method (29,30).

For the POPC + ENHTM3 system, we divided the pulling trajectories into 99 nonuniformly distributed umbrella sampling windows. The initial configurations for the 42 windows nearest the upper membrane leaflet were taken from the pulling where the lipid moved from the upper to the lower leaflet. Conversely, the initial configurations for the 41 windows closest to the lower membrane leaflet were taken from the pulling in the reverse direction. For the 16 windows located near the membrane's center, the configurations' origin alternated. Refer to Table S5 for further details. Sampling within these central windows was enhanced using the Hamiltonian replica exchange (27). Configuration exchanges in these windows were attempted every 100,000 integration steps (200 ps). Every window underwent simulation for 600 ns, with the initial 200 ns designated solely for equilibration. The free energy was calculated from the entire set of umbrella sampling windows using the weighted histogram analysis method (29,30).

Lipid-scrambling experiments

Large unilamellar vesicles

POPC and POPG (13.6 μL and 1.5 μL of 25 mg/mL stock solutions in chloroform) (Avanti Lipids), together with 1-myristoyl-2-C6-NBD-PC (1.5 μL of a 1 mg/mL stock solution in chloroform) (Avanti lipids) were added to a glass test tube. The solvent was evaporated using a gentle stream of nitrogen gas. The test tube was placed in a desiccator attached to a vacuum pump for at least half an hour. Then the peptides (CASLO, Denmark) dissolved in methanol were added in an appropriate amount according to the desired peptide:lipid ratio. Methanol was again evaporated by a gentle stream of nitrogen gas. The test tube was again placed in a desiccator attached to a vacuum pump for at least another 4 h. The dried lipid film was resuspended by vortexing in 0.5 mL of HBS (50 mM HEPES, 150 mM NaCl, pH 7.4) and went through 10 cycles of freeze and thaw. Then the lipid solution was extruded 30 times through a 0.2-μm membrane using an extruder (Avestin). Final lipid concentration is 1 mM.

Fluorescence assay

Liposomes were diluted into final concentration of 0.13 mM into HPS buffer (50 mM HEPES pH 7.4, 150 mM NaCl) in a fluorimetric cuvette, and fluorescence was monitored under constant stirring (1000 rpm) at 20°C in a temperature-controlled Spectrofluorometer Horiba Duetta (lex = 470 nm, lem = 530 nm, excitation band pass 5 nm, emission band pass 10 nm, time increment 10 s, integration time 0.1 s, emission increment 0.5 nm). The sample was equilibrated at least for 45 min before proceeding

with the assays. Then 25 μL of 1 M sodium dithionite (Sigma Aldrich) (final concentration of dithionite was 20 mM), freshly prepared in 0.5 M Tris (pH = 10), was added after 500 s of measurement. Three independent measurements were performed for each system.

RESULTS

Using the coarse-grained Martini 2 force field (12–14), we explored the translocation of an amphiphilic α-helical peptide (TLP) across a phospholipid POPE:POPG (3:1) bilayer in the presence of various α-helical transmembrane proteins/peptides (MPs). Our study utilized a specific 21-amino acid long TLP, referred to as LS9 due to its composition containing nine serines and 12 leucines. We examined over 20 different MPs with diverse amino acid compositions. Our investigation centered on pinpointing the optimal translocation-enhancing sequence by evaluating several key properties of the MPs: 1) the type of hydrophilic residues, 2) depth of hydrophilic residues within the structure, 3) compactness of the hydrophilic patch, 4) presence of charged residues, and 5) type of hydrophobic residues. In all cases, the TLP translocated the membrane employing the same pathway described previously (5,39–42) and depicted in Fig. 2 *A*.

Type of hydrophilic residues

In a prior study (5), we found that the presence of hydrophilic residues in the MP sequence was crucial for enhancing translocation. Consequently, our primary focus was optimizing the hydrophilic amino acids within the MP. We ran simulations on four distinct systems. Each system featured an MP with a hydrophilic patch made of a unique set of hydrophilic residues organized close to each other along the main helical axis. The tested hydrophilic resides were: serines (SAGLS), threonines (SAGLT), glutamines (SAGLQ), and asparagines (SAGLN). The sequences of the MPs used can be found in Table 1.

As illustrated in Fig. 2 *B*, all MPs reduce the free energy of the TLP throughout the membrane region, aiding in the stabilization of the TLP during both its insertion and in its transmembrane state. To measure the ease of TLP translocation across the membrane, we utilized the translocation barrier ($\Delta\Delta G_{\rm BM}$), which is determined by the difference between the highest and lowest free energy in the profile. Each of the four MPs lowers the $\Delta\Delta G_{\rm BM}$ in comparison to translocation without an MP, indicating that TLP's passage through the membrane is facilitated in the presence of these MPs.

The amino acid composition of the MP can influence the free-energy profiles of the TLP. Specifically, asparagines (in MP SAGLN) offer considerably greater stabilization for the TLP compared to serines, threonines, and glutamines. Although the glutamine MP (SAGLQ) reduces the insertion barrier of the TLP relative to the serine and threonine MPs, it slightly undermines the stability of the TLP's transmembrane state, meaning that the free energy of the TLP's transmembrane state is higher. This decreased stability arises from the reduced enthalpic stabilization provided by the glutamines within the MP (see Fig. S1). A summary of free-energy differences in the translocation profiles can be found in Table S6.

The reduction in the translocation barrier is primarily due to the membrane disruption induced by the MP. This membrane disruption manifests itself as membrane thinning,

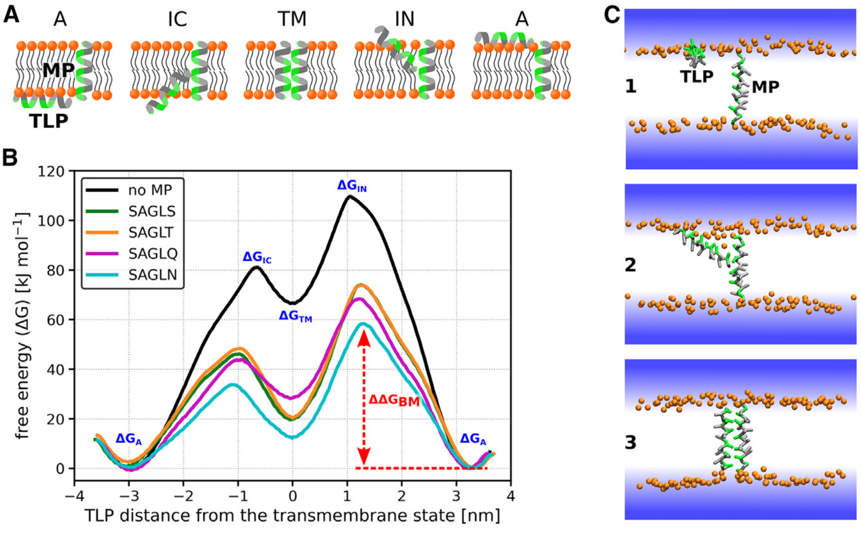

FIGURE 2 Translocation of the LS9 peptide along MPs containing various hydrophilic residues. *A*) The schematic mechanism of the TLP translocation along the MP. Initially, the TLP is adsorbed on the membrane's surface, aligned parallel with the surface. As it inserts, it changes orientation to become perpendicular to the membrane surface. Throughout this process, the TLP's hydrophilic patch orients toward the hydrophilic patch of the MP. (*B*) Free-energy profiles for TLP LS9 translocating alone or along MPs featuring various hydrophilic patch residues: serines (SAGLS), threonines (SAGLT), glutamines (SAGLQ), or asparagines (SAGLN). Notations $\Delta G_{\rm A}$, $\Delta G_{\rm IC}$, $\Delta G_{\rm IN}$, and $\Delta G_{\rm TM}$ refer to the free-energy differences for the adsorbed state (A), local maxima for N and C terminus insertion (IN and IC), and the TLP's transmembrane state (TM), respectively, with the adsorbed state as a reference. The translocation free-energy barrier in the presence of MP SAGLN, $\Delta\Delta G_{\rm BM}$, is highlighted with a red arrow, representing the difference between the profile's highest and lowest free-energy value. Profile errors are estimated to be under 5 kJ mol^{-1}, based on the differences between adsorbed states on both sides of the symmetric membrane. (*C*) Simulation snapshots depicting typical configurations of the system when the TLP is 1) in the adsorbed state, 2) inserting into the membrane, and 3) in the transmembrane state. Orange spheres signify lipid phosphates. Lipid tails are omitted for clarity, whereas TLPs' and MPs' hydrophilic and hydrophobic residues appear in green and gray, respectively. Water is indicated by a blue gradient. To see this figure in color, go online.

slight disorder in the acyl chains (see Fig. S2), and water insertion into the membrane. Note that we observed no continuous water channels or pores around any of the simulated MPs in the membrane (see Fig. S3). We measured the membrane disruption by calculating the water defect in the lipid bilayer surrounding each simulated MP (see Table S6 and the "materials and methods" section for details). A strong correlation between the observed water defects and the $\Delta\Delta G_{BM}$ values is evident, with a correlation coefficient of -0.98. Generally, increased hydrophilicity in the residues that constitute the hydrophilic patch results in a more pronounced water defect, which, in turn, contributes to decreased translocation barriers.

We opted not to determine the free energy of TLP adsorption/desorption, as our prior research (5) showed that similar α-helical MPs did not influence the stability of the TLP's adsorbed states. Consequently, we view these adsorbed states as consistent reference points for the membrane translocation process.

Depth of hydrophilic residues

To explore the connection between translocation enhancement and the placement of hydrophilic residues along the α helix, we designed four α-helical MPs. Each MP was made up of 21 leucines and two asparagines. These MPs were labeled as LNd3, 6, 9, and 12, where the number indicates the average residue depth of the asparagines. A value of 3 signifies that the asparagines are positioned near the N terminus of the MP, whereas 12 indicates proximity to the MP's center. The sequences of these MPs are detailed in Table 1.

Placing the hydrophilic residues closer to the MP's center, and thus closer to the center of the membrane, results in reduced translocation barriers compared to when these residues are situated near the MP's termini. Refer to Fig. 3 *A* and Table S8 for an overview of the relevant free-energy differences.

The decrease of translocation barriers with the deeper insertion of the MP's hydrophilic residues does not align with the water defects observed around each MP. For example, LNd12 produces a notably smaller water defect (42.1 arbitrary units) compared to MP LNd3 (44.0 arbitrary units), even though LNd12 exhibits significantly greater translocation-enhancing properties. As illustrated in Fig. S4 *A*, hydrophilic and charged residues located either at the membrane surface or at its center lead to smaller water defects compared to those placed at an intermediate depth within the membrane. On the other hand, MPs with centrally located hydrophilic/charged residues reduce the density of lipid tails in the membrane's center, as indicated in Fig. S4 *B* and in Fig. S5, but do not affect the water's ability to insert into the membrane core. See Table S7 for the sequences of MPs used for this additional characterization. By utilizing artificially disrupted membranes, we show that this tail defect (TD) accounts for a considerable reduction in the translocation barrier, especially for MPs with hydrophilic/charged residues located near the membrane's center (see Fig. S4 *D* and *E*).

Interestingly, even though the MPs discussed in this section were asymmetric, we did not find significant variations in the free energies when comparing different directions of translocation through the membrane, as shown in Fig. S6.

Hydrophilic patch compactness

In this section, we explored the effect of hydrophilic patch compactness, i.e., the arrangement of hydrophilic residues around the α helix of the MP, on the translocation enhancement of TLP. By analyzing six MPs composed of leucine and serine residues with different distributions around the α helix, we determined that the compactness of the hydrophilic patch is crucial for effective translocation enhancement. The MPs examined are labeled as SAGLS9g0–5, where the concluding number represents the extent of disruption in their hydrophilic patch. Refer to Table 1 for the sequences of these MPs and to Fig. S7 *A* for their helical wheel diagrams.

MPs with hydrophilic residues oriented in the same direction (those with compact patches) enhance translocation more effectively than MPs where hydrophilic residues are oriented in diverse directions (those with disrupted patches). Even a minor disruption in the hydrophilic patch's compactness, as seen in SAGLS9g1, can lead to substantially elevated translocation barriers compared to a fully compact patch (see Fig. 3 *B*). As the level of patch disruption intensifies, $\Delta\Delta G_{BM}$ values continue to rise up to a certain threshold. $\Delta\Delta G_{BM}$ value for the TLP in the presence of SAGLS9g5 is then somewhat reduced again, which can be attributed to the formation of a secondary hydrophilic patch by the MP's hydrophilic residues (see Fig. S8). The free-energy profiles of TLP translocation along these MPs can be found in Fig. S7 and a breakdown of the free-energy differences is provided in Table S9.

Two primary factors are responsible for the reduced translocation enhancement by MPs with disrupted hydrophilic patches. Firstly, the membrane disruption induced by the hydrophilic residues is not as concentrated in the region where the TLP translocates. Secondly, MPs with notably disrupted hydrophilic patches have weaker interactions with the TLP, resulting in less enthalpic stabilization for the TLP during its translocation, as depicted in Fig. S7 *C*.

Presence of charged residues

To understand the influence of charged residues on translocation enhancement, we examined five distinct polyleucine MPs containing one charged residue in their center. Among these, SAGLE1 featured a negatively charged glutamate, whereas SAGLK1 incorporated a positively charged lysine. Two variations of SAGLE1 were also investigated: SAGLE^{0}1 with its glutamate protonated (rendering it uncharged) and SAGLE^{+}1, which contained an artificial positively charged counterpart termed "anti-glutamate." Additionally, MP SAGLS6E1 was derived from SAGLS,

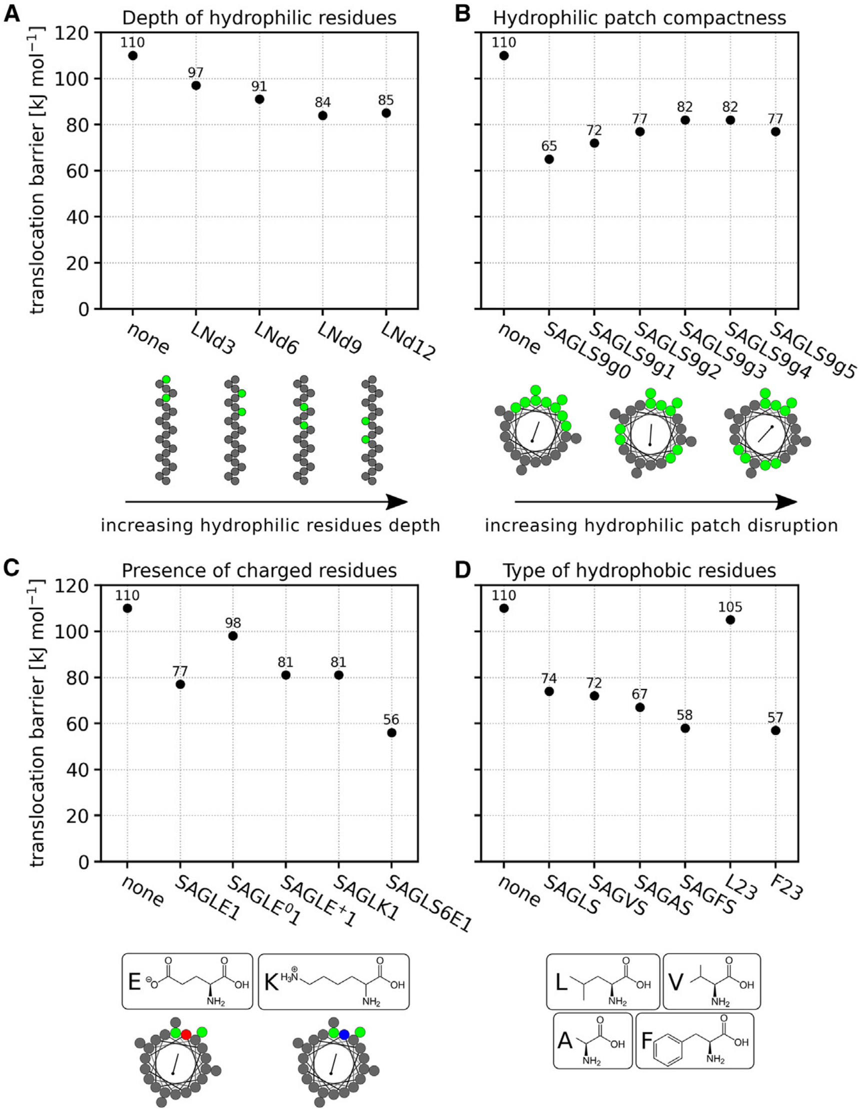

FIGURE 3 Translocation barriers for TLP LS9 in systems with various MPs. (*A*) Dependence of translocation barriers on the depth of MP's hydrophilic residues. As the hydrophilic residues become positioned deeper in the membrane, barriers for TLP translocation generally decrease. (*B*) Dependence of translocation barriers on the compactness of the MP's hydrophilic patch. Increasing disruption of the hydrophilic patch results in increased translocation barriers. (*C*) Dependence of translocation barriers on the presence of a charged residue in the MP. The introduction of charged amino acid side chains significantly decreases the translocation barrier for the TLP. (*D*) Dependence of translocation barriers on the hydrophobic residues present in the MP. Phenylalanine stands out as the most effective in enhancing TLP translocation among the residues studied. For detailed free-energy profiles of TLP translocation in the presence of these MPs, see the supporting material. To see this figure in color, go online.

where one centrally located serine was substituted with a negatively charged glutamate. The sequences of these MPs are detailed in Table 1.

A single charged residue within MPs significantly affects TLP translocation (see Fig. 3 *C*). The inclusion of just one charged residue, be it glutamate, lysine, or anti-glutamate, affects the translocation barrier nearly as much as having five serines in the SAGLS sequence. Incorporating a charged residue into an MP already possessing a hydrophilic patch, as with MP SAGLS6E1, further amplifies its ability to enhance translocation. For computed free-energy profiles and free-energy differences, refer to Fig. S9 *A* and Table S10, respectively.

The pronounced influence of glutamate, lysine, and anti-glutamate primarily arises from the charge of their side chains. Neutralizing the charge markedly diminishes the MP's capability to enhance translocation. These charged side chains induce a significant TD, as detailed in Table S10, accounting for this enhancement in translocation. However, the charge of these residues does not notably alter the interaction strength between the TLP and the MP, as indicated in Fig. S9 *B*.

Type of hydrophobic residues

To assess the impact of hydrophobic residues on translocation enhancement, we designed six MPs primarily varying in their hydrophobic residue composition. We compared the effects of leucine (SAGLS, L23), phenylalanine (SAGFS, F23), valine (SAGVS), and alanine (SAGAS). The MPs L23 and F23 consisted solely of leucines and phenylalanines, respectively. In contrast, the other MPs incorporated a hydrophilic serine patch. Refer to Table 1 for the sequences of the MPs under consideration.

Rather unexpectedly, hydrophobic residues can markedly influence translocation enhancement, as illustrated in Fig. 3 *D*. Although leucines and valines demonstrate similar translocation-enhancing capabilities, alanines marginally reduce the TLP's translocation barrier when compared to leucines. Phenylalanines, however, have the most pronounced impact on TLP translocation. This is further demonstrated by MP F23, which consists solely of phenylalanines. F23 proved to be a very strong translocation enhancer, in contrast to MP L23, a fully hydrophobic peptide composed of 23 leucines. See Fig. S10 *A* for the calculated free-energy profiles and Table S11 for the relevant free-energy differences in the profiles.

Phenylalanine's marked influence on translocation enhancement arises from a combination of two factors. Firstly, the presence of phenylalanines induces a notable membrane disruption, as observed with both F23 and SAGFS (see Table S11). This disruption is likely attributed to the bulky nature of phenylalanine's side chains, which interfere with the lipid packing of the membrane. Secondly, the aromatic nature of the phenylalanine side chains facilitates strong interactions with the hydrophobic residues of the TLP, resulting in substantial enthalpic stabilization, as shown in Fig. S10 *B*.

Optimizing the MP sequence

We designed several MPs that combined multiple translocation-enhancing properties and evaluated their effect on TLP translocation. ENHTM1 features a compact hydrophilic patch composed of asparagines, a centrally positioned charged glutamate, and phenylalanines as hydrophobic residues. ENHTM2 is similar to ENHTM1 but has a smaller hydrophilic patch and contains three glutamates instead of one. ENHTM3 possesses more modest features, with leucines, a large and compact hydrophilic patch composed of serines, and a single positively charged lysine at the center of the peptide. The sequences of these MPs are detailed in Table 1.

As demonstrated in Fig. 4, all of the MPs significantly reduce the translocation free-energy barrier of the TLP. ENHTM1 and ENHTM2 offer similar stabilization with translocation barriers of just 35 and 31 kJ mol^{-1}, respectively. ENHTM3 has a translocation barrier of 58 kJ mol^{-1}, which is comparable to the MP SAGLS6E1 mentioned earlier.

Overall, these results suggest that the translocation-enhancing properties described herein are largely additive and can be synergistically combined for further translocation enhancement.

Exploring the relationship between $\Delta\Delta G_{BM}$ and ΔG_{TM}

To further explore the translocation-enhancing abilities of the MPs, we examined the relationship between the translocation barriers, $\Delta\Delta G_{\mathrm{BM}}$, and the free energy of transmembrane states, ΔG_{TM}, across all simulated systems. As shown in Fig. S11, we found that these properties are linearly correlated, indicating that there are no MPs that stabilize the insertion barrier of the TLP without also stabilizing its transmembrane state, or vice versa. This implies that the difficulty of the TLP translocating along an MP, given by the height of the insertion maxima, can be estimated by analyzing the stability of the TLP's transmembrane state compared to its adsorbed state.

Translocation across POPC membranes

All of our above simulations employed a POPE:POPG (3:1) membrane to mimic the bacterial inner membrane. For several MPs, we also explored TLP translocation across a POPC membrane. We observed that trends for POPC membranes mirrored those for POPE:POPG membranes, with stronger translocation enhancement being associated with larger membrane disruption. Overall, translocation of the LS peptide through the POPC membrane was easier, with translocation barriers consistently roughly 10 kJ mol^{-1} lower than those in the corresponding POPE:POPG systems. See Fig. S12 and Table S12 for the free-energy profiles and free-energy differences calculated for systems with the POPC membrane.

Lipid scrambling by translocation enhancers

Finally, we probed the potential of a chosen MP, ENHTM3, to promote the translocation of other amphiphilic

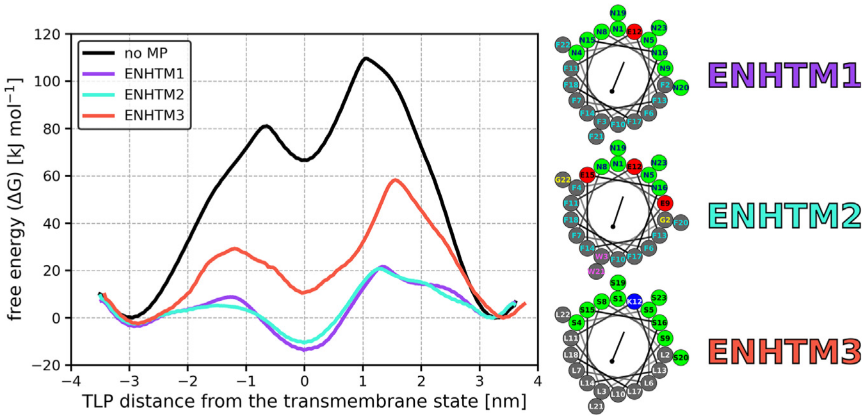

FIGURE 4 Comparison of MPs combining multiple translocation-enhancing properties. Free-energy profiles of TLP LS9 translocating through the membrane alone (*black*) or in the presence of MP ENHTM1 (*purple*), ENHTM2 (*teal*), or ENHTM3 (*red*). Profile errors are below 5 kJ mol^{-1} based on the asymmetry of the adsorbed states. ENHTM1 and ENHTM2 decrease the translocation barrier more than any other simulated MP. The helical wheels showing the composition of the MPs and the position of their residues are shown to the right of the chart. To see this figure in color, go online.

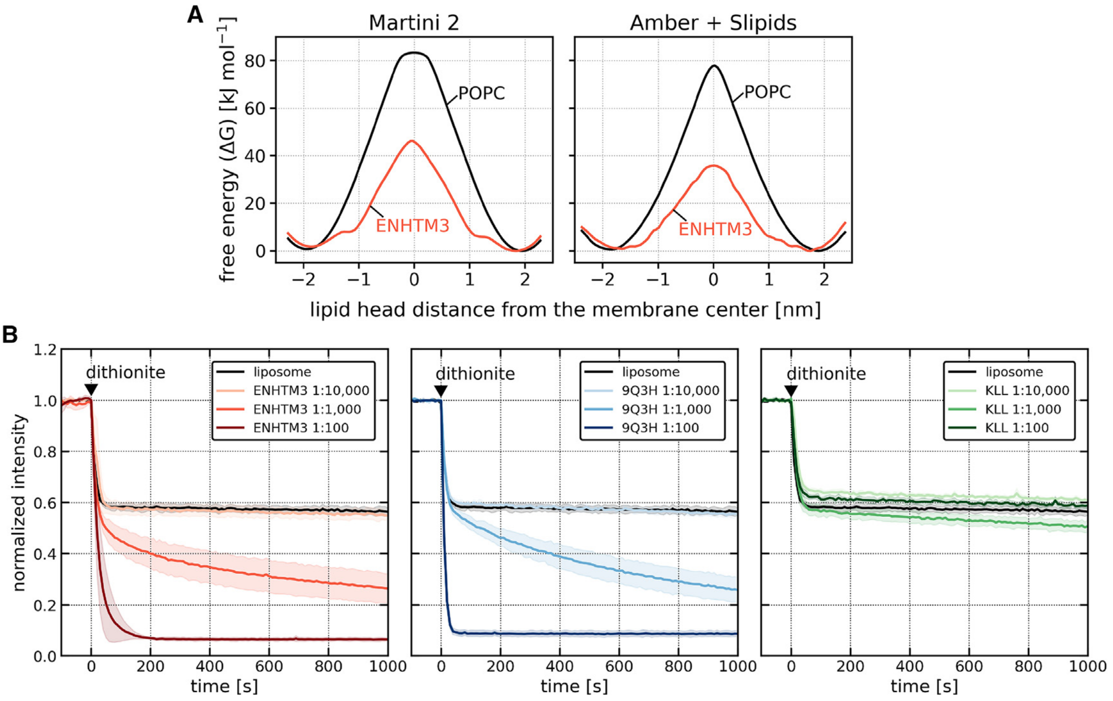

FIGURE 5 Lipid flip-flop facilitated by MPs. (*A*) Free-energy profiles of lipid flip-flop in a pure POPC bilayer (*black curve*) versus a POPC membrane with the embedded MP ENHTM3 (*red curve*). The left chart presents results from the coarse-grained Martini 2 force field, whereas the right chart corresponds to the atomistic Amber + Slipids force field. (*B*) Time course of NBD-PC fluorescence, normalized to its initial value, after dithionite addition. Black curves reflect fluorescence in pure LUVs, whereas colored curves represent LUVs containing peptide ENHTM3 (*left*), 9Q3H (*middle*), or KLL (*right*) at three distinct peptide:lipid (mol:mol) ratios. Decreased fluorescence intensity is indicative of increased scrambling rate. Each line represents the mean normalized intensity derived from three independent measurements, with the shading indicating ± 1 standard deviation from the mean. To see this figure in color, go online.

molecules, specifically phospholipids. We opted for the ENHTM3 over the ENHTM1 and ENHTM2 MPs. This decision was underpinned by its feasibility for synthesis for subsequent experimental validation and by its predicted greater propensity to form an α helix, attributed to the presence of leucines rather than phenylalanines. Indeed, the ENHTM3 remained in α-helical conformation during our atomistic simulations described below.

Our initial step involved calculating the free energy associated with the flip-flop of a POPC lipid when facilitated by the MP ENHTM3, employing both the coarse-grained Martini 2 force field and the atomistic Amber 99SB-ILDN force field complemented with Slipids 2020 force field. Fig. 5 *A* illustrates a pronounced reduction in the free-energy barrier for the flip-flop of POPC in the presence of ENHTM3 across both computational models (with a decrease from 83 to 46 kJ mol^{-1} in Martini 2, and from 78 to 36 kJ mol^{-1} in Amber + Slipids).

This computational observation was subsequently validated through a fluorescence assay employing NBD-PC lipids embedded within large unilamellar vesicles (LUVs) (10,43). For this experimental validation, we also used a polyleucine MP KLL, devoid of any translocation-enhancing attributes, as a benchmark for negative control. Simultaneously, lipid-scrambling peptide 9Q3H (8) was introduced as a positive control. See Table 1 for the sequences of these peptides. Experimentally, the scrambling rate recorded for ENHTM3 was similar to that observed for the positive control 9Q3H, as seen in Fig. 5 *B*. In contrast, the KLL peptide, our negative control, exhibited negligible lipid scrambling regardless of its concentration.

These findings demonstrate that the general features of translocation-enhancing MPs assist not only in peptide translocation but also in inducing lipid flip-flop, and the simulation results of decreased free-energy barriers are in agreement with experiments.

DISCUSSION

We investigated the effect of various properties of α-helical transmembrane proteins/peptides (MPs) on the membrane translocation of a representative leucine-serine α-helical TLP utilizing the Martini 2 coarse-grained model. Our analysis centered on the type of the hydrophilic residues, their position along the α helix (residue depth), the compactness of the hydrophilic patch of the MP, the incorporation of charged residues, and the type of hydrophobic residues within the MP. Each simulation specifically addressed the translocation of one TLP along one MP, underscoring our focus on the translocation process at low peptide concentrations. We focused on peptide translocation across POPE:POPG (3:1)

membranes, which mimic bacterial inner membranes, and also conducted several simulations with POPC membranes. No qualitative differences were observed between the two. We postulate that our general conclusions are valid for all biologically relevant phospholipid membrane types.

We observed the same translocation pathway as in our prior research (5), where the TLP, initially adsorbed on the membrane surface in orientation parallel with the membrane plane, shifts to a perpendicular orientation in the transmembrane state while interacting with the MP. This mode of translocation aligns with findings from previous peptide translocation studies (26,39,40,42,44). However, it is worth noting that nonamphiphilic or non-α-helical TLPs might navigate through the phospholipid bilayer differently based on their distinct attributes, such as using the mechanism outlined for NAF-1 (45).

Based on our simulations, peptide translocation through biological membranes can be facilitated by incorporating an MP with specific properties into the membrane. These properties encompass the presence of hydrophilic, charged, and/or aromatic hydrophobic residues. Although we have tested only phenylalanines among the aromatic residues, it is reasonable to assume that other similar aromatic residues, such as tryptophan, would exhibit similar behavior.

As previously demonstrated, increasing the number of hydrophilic residues in the MP's sequence increases its translocation-enhancing abilities (5). In addition to this, the translocation enhancement can further be improved by incorporating hydrophilic residues with lower hydrophobicity, such as asparagines in place of serines. This observation is somewhat applicable to hydrophobic residues as well; for instance, using the less hydrophobic alanines instead of leucines slightly elevates the MP's translocation-enhancing abilities. Nonetheless, it is important to highlight that phenylalanine remains a significantly more effective translocation-enhancing residue than alanine, despite its higher hydrophobicity.

The arrangement of translocation-enhancing residues within the MP's structure is also crucial. For optimal translocation enhancement, the hydrophilic residues need to form a patch that guides the TLP's translocation. Spreading hydrophilic residues in varying patterns around the MP's α helix consistently results in reduced translocation enhancement. Additionally, for superior translocation enhancement, it is more beneficial to position the hydrophilic or charged residues near the membrane's center rather than its surface.

We observed that the employed MPs generally stabilize both the insertion barrier and the transmembrane state of the TLP. This indicates that the translocation ability of the TLP can be estimated by examining the stability of its transmembrane state, eliminating the need to calculate the entire translocation pathway. However, this method should be limited to comparing the translocation of a single TLP along various MPs. It is not suitable for comparing the translocation abilities of different TLPs, as the linear relationship between translocation difficulty and the stability of the transmembrane state often does not apply to them (26).

Increasing the translocation-enhancing capabilities of an MP can often result in two outcomes: 1) reduced MP stability within the membrane, and 2) a decrease in the α-helical propensity of the MP. Concerning the first challenge, it is essential to strike a careful balance between the MP's stability in the membrane and its translocation-enhancing properties. In our studies, all the MPs from Table 1 remained stable in their transmembrane states in the POPE:POPG (3:1) membrane. Additionally, we propose that MPs with even more potent translocation-enhancing features, perhaps due to the addition of more hydrophilic/charged residues, might be viable if incorporated into a larger, adequately hydrophobic protein structure. This would anchor the translocation-enhancing segment securely within the membrane.

Given the constraints of the employed coarse-grained model, which maintains the secondary structure of our MPs and TLPs during simulations, we have not explored the second concern regarding the α-helical propensity of the MPs. Still, we postulate that the identified translocation-enhancing attributes are also applicable to MPs without an α-helical conformation in their transmembrane part as well as to more extensive protein structures. This notion stems from the fact that the guiding geometrical principles, such as the ideal position and orientation of the translocation-enhancing residues within the structure, are not reliant on a specific secondary structure. Instead, they are based on the relative positioning of residues concerning one another and the membrane's normal.

In addition to establishing the guidelines for designing translocation-enhancing MPs, we delved into the mechanistic underpinnings of the translocation enhancement. In our previous work (5), we demonstrated that translocation-enhancing MPs disrupt the membrane, leading to water insertion into the membrane without the formation of continuous water channels. This phenomenon can be measured by evaluating the water defect (i.e., the average water density surrounding the MP). Disrupted membrane offers a more accessible path for the TLP, resulting in a strong correlation between the water defect and the MP's translocation-enhancing capacities. Formation of a water defect, even if formed by the TLP itself, has, in fact, been recently suggested to enhance the translocation of charged peptides (46). Additionally, water defect is related to membrane thinning, which has been proposed to enhance the insertion and translocation of proteins by certain protein translocases (47). Other than water defect, our study also revealed that the MP offers enthalpic stabilization to the TLP during translocation, stemming from the attractive interactions between the residues of the TLP and MP.

In this study, we not only validate our prior observations but also demonstrate that, when the membrane is disrupted away from its surface, this disturbance does not always appear as a water defect. Instead, it can manifest as a reduced density of lipid tails at the membrane's core.

Such a tail defect (TD) still plays a pivotal role in translocation by making it easier for the TLP to traverse the membrane. Furthermore, we underscore that enthalpic stabilization does not solely depend on interactions between hydrophilic residues. Aromatic residues, particularly phenylalanine, can also provide potent enthalpic stabilization to the TLP.

We propose that enhancement of translocation originates from three primary factors: 1) wet membrane disruption, i.e., membrane thinning, which relates to water insertion into the membrane core and arises due to translocation-enhancing residues positioned sufficiently close to the membrane surface; 2) dry membrane disruption, which does not relate to water insertion and is attributed to centrally positioned translocation-enhancing residues; and 3) stabilizing interactions between the TLP and the MP.

It should be noted that the membranes used in this study were all symmetric in their lipid composition, whereas many biological membranes are asymmetric (48,49). In asymmetric membranes, the disruption caused by MPs in each membrane leaflet could vary, leading to complex effects on the TLP translocation. Furthermore, TLPs have been reported to be affected by membrane asymmetry during the translocation process (50). Although we expect that our general conclusions would hold for all phospholipid membranes, a detailed exploration of the relationship between MPs and asymmetric membranes would need further research.

The design of our MPs drew inspiration from scramblases, proteins that enable the bidirectional flip-flop of phospholipids (6–10). We therefore tested scrambling activity of one of our MPs using coarse-grained simulations, all-atom simulations, and fluorescence experiments. In the presence of this MP, we saw a substantial reduction in the flip-flop barrier and an accelerated rate of scrambling. These results indicate that translocation-enhancing properties can also boost phospholipid flip-flop. We propose that the translocation-enhancing properties and the structural mechanisms we discussed might be typical for proteins aiding in both peptide membrane insertion and lipid scrambling. Indeed, recent experimental data, unbiased molecular dynamics simulations (51), and free-energy calculations (52) suggest that insertases, which assist in embedding proteins or protein domains into membranes, can also promote lipid scrambling through their hydrophilic patches or cavities. Additionally, a very recent preprint hints at scramblases being able to also facilitate the translocation of amphipathic drug molecules (53). This introduces the possibility that the mechanisms facilitating peptide translocation and lipid scrambling might extend to the membrane translocation of amphiphiles in general.

Finally, we suggest that scramblases, insertases, or similar proteins naturally occurring in biological membranes could be exploited by antimicrobial peptides or CPPs to more easily enter the interior of the cell. Alternatively, peptide mixtures containing translocation enhancers could be designed for enhanced peptide transport into cells. Given that the majority of natural AMPs carry a positive charge (54), utilizing specifically charged MPs—either positive or negative—could result in selective translocation enhancement, offering more precise control over the peptide translocation process. Exploring this option, however, falls outside the scope of this study.

CONCLUSIONS

We explored the influence of transmembrane proteins/peptides with diverse properties on peptide translocation using coarse-grained molecular dynamics coupled with free-energy calculations. Our findings reveal that the presence of a compact hydrophilic patch, charged residues, and aromatic residues with bulky side chains in the transmembrane protein/peptide significantly enhances peptide translocation across the membrane. Based on this, we offer detailed guidelines for designing translocation-enhancing transmembrane proteins/peptides.

Moreover, we pinpoint three primary mechanisms underlying this enhanced translocation: membrane disruption linked to water defects, membrane disruption without water defects but with reduced lipid tail density, and stabilizing interactions between peptides/proteins.

Through both coarse-grained and atomistic free-energy calculations, complemented by experimental fluorescence assays, we demonstrate that these translocation-enhancing properties also aid in phospholipid scrambling. This leads us to postulate that the characteristics we have outlined might be ubiquitous among proteins that facilitate the insertion or translocation of amphiphilic molecules into or across phospholipid membranes, such as scramblases and insertases.

We further propose that scramblases and insertases could be exploited to enhance the transport of antimicrobial peptides or CPPs through cell membranes. Another prospect is the potential synergistic effect of using a pair of peptides: a highly hydrophobic peptide with a hydrophilic patch and/or charged residues could embed into the membrane and subsequently enhance the translocation of another, more hydrophilic peptide.

SUPPORTING MATERIAL

Supporting Material can be found online at https://doi.org/10.1016/j.bpj.2024.04.009.

AUTHOR CONTRIBUTIONS

L.B. carried out the molecular dynamics simulations and analyzed the data. M.D. performed the fluorescence assay experiments. R.V. designed the research. L.B., M.D., and R.V. wrote the article.

ACKNOWLEDGMENTS

This work was supported by the European Research Council (ERC) under the European Union's Horizon 2020 research and innovation programme

(grant agreement no. 101001470) (R.V.) and the project National Institute of virology and bacteriology (Programme EXCELES, ID project no. LX22NPO5103) funded by the European Union - Next Generation EU (R.V.). Computational resources were provided by the CESNET, CERIT Scientific Cloud, and IT4 Innovations National Supercomputing Center by MEYS CR through the e-INFRA CZ (ID: 90254). We acknowledge IT4 Innovations National Supercomputing Center for awarding this project access to the LUMI supercomputer, owned by the EuroHPC Joint Undertaking, hosted by CSC (Finland) and the LUMI consortium through the e-INFRA CZ (ID: 90254).

We acknowledge the use of the ChatGPT tool to enhance the clarity, coherence, and overall quality of the writing.

DECLARATION OF INTERESTS

The authors declare no competing interests.

REFERENCES

1. Wang, J., X. Dou, …, A. Shan. 2019. Antimicrobial peptides: Promising alternatives in the post feeding antibiotic era. *Med. Res. Rev.* 39:831–859. https://doi.org/10.1002/med.21542.
2. Guidotti, G., L. Brambilla, and D. Rossi. 2017. Cell-Penetrating Peptides: From Basic Research to Clinics. *Trends Pharmacol. Sci.* 38:406–424. https://linkinghub.elsevier.com/retrieve/pii/S0165614717300172.
3. Kabelka, I., and R. Vácha. 2021. Advances in Molecular Understanding of α-Helical Membrane-Active Peptides. *Acc. Chem. Res.* 54:2196–2204. https://doi.org/10.1021/acs.accounts.1c00047.
4. Avci, F. G., B. S. Akbulut, and E. Ozkirimli. 2018. Membrane Active Peptides and Their Biophysical Characterization. *Biomolecules.* 8:77. https://pubmed.ncbi.nlm.nih.gov/30135402.
5. Bartoš, L., I. Kabelka, and R. Vácha. 2021. Enhanced translocation of amphiphilic peptides across membranes by transmembrane proteins. *Biophys. J.* 120:2296–2305. https://www.sciencedirect.com/science/article/pii/S0006349521003003.
6. Hankins, H. M., R. D. Baldridge, …, T. R. Graham. 2015. Role of Flippases, Scramblases and Transfer Proteins in Phosphatidylserine Subcellular Distribution. *Traffic.* 16:35–47. https://doi.org/10.1111/tra.12233.
7. Ernst, O. P., and A. K. Menon. 2015. Phospholipid scrambling by rhodopsin. *Photochem. Photobiol. Sci.* 14:1922–1931. https://doi.org/10.1039/c5pp00195a.
8. Nakao, H., C. Hayashi, …, M. Nakano. 2018. Effects of Hydrophilic Residues and Hydrophobic Length on Flip-Flop Promotion by Transmembrane Peptides. *J. Phys. Chem. B.* 122:4318–4324. https://doi.org/10.1021/acs.jpcb.8b00298.
9. Arndt, M., C. Alvadia, …, R. Dutzler. 2022. Structural basis for the activation of the lipid scramblase TMEM16F. *Nat. Commun.* 13:6692. https://www.nature.com/articles/s41467-022-34497-x.
10. Jahn, H., L. Bartoš, …, A. K. Menon. 2023. Phospholipids are imported into mitochondria by VDAC, a dimeric beta barrel scramblase. *Nat. Commun.* 14:8115. https://www.nature.com/articles/s41467-023-43570-y.
11. Abraham, M. J., T. Murtola, …, E. Lindahl. 2015. GROMACS: High performance molecular simulations through multi-level parallelism from laptops to supercomputers. *SoftwareX.* 1–2:19–25. https://linkinghub.elsevier.com/retrieve/pii/S2352711015000059.
12. Marrink, S. J., H. J. Risselada, …, A. H. de Vries. 2007. The MARTINI Force Field: Coarse Grained Model for Biomolecular Simulations. *J. Phys. Chem. B.* 111:7812–7824. https://doi.org/10.1021/jp071097f.
13. Monticelli, L., S. K. Kandasamy, …, S.-J. Marrink. 2008. The MARTINI Coarse-Grained Force Field: Extension to Proteins. *J. Chem. Theor. Comput.* 4:819–834. https://doi.org/10.1021/ct700324x.
14. de Jong, D. H., G. Singh, …, S. J. Marrink. 2013. Improved Parameters for the Martini Coarse-Grained Protein Force Field. *J. Chem. Theor. Comput.* 9:687–697. https://doi.org/10.1021/ct300646g.
15. Javanainen, M., H. Martinez-Seara, and I. Vattulainen. 2017. Excessive aggregation of membrane proteins in the Martini model. *PLoS One.* 12, e0187936. https://doi.org/10.1371/journal.pone.0187936.
16. Spinti, J. K., F. Neiva Nunes, and M. N. Melo. 2023. Room for improvement in the initial martini 3 parameterization of peptide interactions. *Chem. Phys. Lett.* 819, 140436. https://linkinghub.elsevier.com/retrieve/pii/S0009261423001410.
17. Jo, S., T. Kim, …, W. Im. 2008. CHARMM-GUI: A web-based graphical user interface for CHARMM. *J. Comput. Chem.* 29:1859–1865. https://doi.org/10.1002/jcc.20945.
18. Šali, A., and T. L. Blundell. 1993. Comparative Protein Modelling by Satisfaction of Spatial Restraints. *J. Mol. Biol.* 234:779–815. https://doi.org/10.1006/jmbi.1993.1626.
19. Gautier, R., D. Douguet, …, G. Drin. 2008. HELIQUEST: a web server to screen sequences with specific -helical properties. *Bioinformatics.* 24:2101–2102. https://doi.org/10.1093/bioinformatics/btn392.
20. Bussi, G., D. Donadio, and M. Parrinello. 2007. Canonical sampling through velocity rescaling. *J. Chem. Phys.* 126, 014101. https://doi.org/10.1063/1.2408420.
21. Berendsen, H. J. C., J. P. M. Postma, …, J. R. Haak. 1984. Molecular dynamics with coupling to an external bath. *J. Chem. Phys.* 81:3684–3690. https://doi.org/10.1063/1.448118.
22. Parrinello, M., and A. Rahman. 1980. Crystal Structure and Pair Potentials: A Molecular-Dynamics Study. *Phys. Rev. Lett.* 45:1196–1199. https://doi.org/10.1103/PhysRevLett.45.1196.
23. Parrinello, M., and A. Rahman. 1981. Polymorphic transitions in single crystals: A new molecular dynamics method. *J. Appl. Phys.* 52:7182–7190. https://doi.org/10.1063/1.328693.
24. Torrie, G. M., and J. P. Valleau. 1974. Monte Carlo free energy estimates using non-Boltzmann sampling: Application to the sub-critical Lennard-Jones fluid. *Chem. Phys. Lett.* 28:578–581.
25. Torrie, G., and J. Valleau. 1977. Nonphysical sampling distributions in Monte Carlo free-energy estimation: Umbrella sampling. *J. Comput. Phys.* 23:187–199. https://linkinghub.elsevier.com/retrieve/pii/0021999177901218.
26. Brožek, R., I. Kabelka, and R. Vácha. 2020. Effect of Helical Kink on Peptide Translocation across Phospholipid Membranes. *J. Phys. Chem. B.* 124:5940–5947. https://doi.org/10.1021/acs.jpcb.0c03291.
27. Fukunishi, H., O. Watanabe, and S. Takada. 2002. On the Hamiltonian replica exchange method for efficient sampling of biomolecular systems: Application to protein structure prediction. *J. Chem. Phys.* 116:9058–9067. https://doi.org/10.1063/1.1472510.
28. Tribello, G. A., M. Bonomi, …, G. Bussi. 2014. PLUMED 2: New feathers for an old bird. *Comput. Phys. Commun.* 185:604–613. https://doi.org/10.1016/j.cpc.2013.09.018.
29. Kumar, S., J. M. Rosenberg, …, P. A. Kollman. 1992. The weighted histogram analysis method for free-energy calculations on biomolecules. I. The method. *J. Comput. Chem.* 13:1011–1021. https://doi.org/10.1002/jcc.540130812.
30. Souaille, M., and B. Roux. 2001. Extension to the weighted histogram analysis method: combining umbrella sampling with free energy calculations. *Comput. Phys. Commun.* 135:40–57. https://linkinghub.elsevier.com/retrieve/pii/S0010465500002150.
31. Hub, J. S., B. L. de Groot, and D. van der Spoel. 2010. g_wham—A Free Weighted Histogram Analysis Implementation Including Robust Error and Autocorrelation Estimates. *J. Chem. Theor. Comput.* 6:3713–3720. https://doi.org/10.1021/ct100494z.
32. Jämbeck, J. P. M., and A. P. Lyubartsev. 2012. Derivation and Systematic Validation of a Refined All-Atom Force Field for Phosphatidylcholine Lipids. *J. Phys. Chem. B.* 116:3164–3179. https://doi.org/10.1021/jp212503e.
33. Jämbeck, J. P. M., and A. P. Lyubartsev. 2012. An Extension and Further Validation of an All-Atomistic Force Field for Biological

Membranes. *J. Chem. Theor. Comput.* 8:2938–2948. https://doi.org/10.1021/ct300342n.

34. Grote, F., and A. P. Lyubartsev. 2020. Optimization of Slipids Force Field Parameters Describing Headgroups of Phospholipids. *J. Phys. Chem. B.* 124:8784–8793. https://doi.org/10.1021/acs.jpcb.0c06386.
35. Klauda, J. B., R. M. Venable, …, R. W. Pastor. 2010. Update of the CHARMM All-Atom Additive Force Field for Lipids: Validation on Six Lipid Types. *J. Phys. Chem. B.* 114:7830–7843. https://doi.org/10.1021/jp101759q.
36. Hanwell, M. D., D. E. Curtis, …, G. R. Hutchison. 2012. Avogadro: an advanced semantic chemical editor, visualization, and analysis platform. *J. Cheminf.* 4:17. https://doi.org/10.1186/1758-2946-4-17.
37. Hess, B., H. Bekker, …, J. G. E. M. Fraaije. 1997. LINCS: A linear constraint solver for molecular simulations. *J. Comput. Chem.* 18:1463–1472. https://doi.org/10.1002/(SICI)1096-987X(199709)18:12<1463::AID-JCC4>3.0.CO;2-H.
38. Essmann, U., L. Perera, …, L. G. Pedersen. 1995. A smooth particle mesh Ewald method. *J. Chem. Phys.* 103:8577–8593. https://pubs.aip.org/aip/jcp/article/103/19/8577-8593/180219.
39. Kabelka, I., and R. Vácha. 2018. Optimal Hydrophobicity and Reorientation of Amphiphilic Peptides Translocating through Membrane. *Biophys. J.* 115:1045–1054. https://doi.org/10.1016/j.bpj.2018.08.012.
40. Ulmschneider, J. P. 2017. Charged Antimicrobial Peptides Can Translocate across Membranes without Forming Channel-like Pores. *Biophys. J.* 113:73–81. https://linkinghub.elsevier.com/retrieve/pii/S0006349517306239.
41. Irudayam, S. J., and M. L. Berkowitz. 2012. Binding and reorientation of melittin in a POPC bilayer: Computer simulations. *Biochim. Biophys. Acta.* 1818:2975–2981. https://linkinghub.elsevier.com/retrieve/pii/S0005273612002581.
42. Chetwynd, A., C. L. Wee, …, M. S. P. Sansom. 2010. The Energetics of Transmembrane Helix Insertion into a Lipid Bilayer. *Biophys. J.* 99:2534–2540. https://linkinghub.elsevier.com/retrieve/pii/S0006349510009689.
43. Wang, L., Y. Iwasaki, …, P. Bütikofer. 2018. Scrambling of natural and fluorescently tagged phosphatidylinositol by reconstituted G protein–coupled receptor and TMEM16 scramblases. *J. Biol. Chem.* 293:18318–18327. https://linkinghub.elsevier.com/retrieve/pii/S0021925820312126.
44. Hall, B. A., A. P. Chetwynd, and M. S. P. Sansom. 2011. Exploring Peptide-Membrane Interactions with Coarse-Grained MD Simulations. *Biophys. J.* 100:1940–1948. https://linkinghub.elsevier.com/retrieve/pii/S000634951100261X.
45. Cardenas, A. E., C. I. Drexler, …, R. Elber. 2022. Peptide Permeation across a Phosphocholine Membrane: An Atomically Detailed Mechanism Determined through Simulations and Supported by Experimentation. *J. Phys. Chem. B.* 126:2834–2849. https://doi.org/10.1021/acs.jpcb.1c10966.
46. Elber, R. 2023. Defect Formation and Peptide Permeation across Phospholipid Membranes. *J. Phys. Chem. B.* 127:7810–7818. https://doi.org/10.1021/acs.jpcb.3c04895.
47. Wu, X., and T. A. Rapoport. 2021. Translocation of Proteins through a Distorted Lipid Bilayer. *Trends Cell Biol.* 31:473–484. https://linkinghub.elsevier.com/retrieve/pii/S0962892421000064.
48. Devaux, P. F. 1991. Static and dynamic lipid asymmetry in cell membranes. *Biochemistry.* 30:1163–1173. https://doi.org/10.1021/bi00219a001.
49. Doktorova, M., J. L. Symons, …, I. Levental. 2023. Cell Membranes Sustain Phospholipid Imbalance Via Cholesterol Asymmetry. Preprint at bioRxiv. https://www.biorxiv.org/content/early/2023/07/31/2023.07.30.551157.
50. Bartoš, L., and R. Vácha. 2024. Peptide translocation across asymmetric phospholipid membranes. *Biophys. J.* 123:693–702. https://linkinghub.elsevier.com/retrieve/pii/S000634952400105X.
51. Li, D., C. Rocha-Roa, …, S. Vanni. 2023. Lipid scrambling is a general feature of protein insertases. Preprint at bioRxiv. https://www.biorxiv.org/content/early/2023/09/01/2023.09.01.555937.
52. Bartoš, L., A. K. Menon, and R. Vácha. 2024. Insertases scramble lipids: Molecular simulations of MTCH2. *Structure.* https://linkinghub.elsevier.com/retrieve/pii/S0969212624000364.
53. Herrero, C. G., and S. Thallmair. 2024. GPCR surface creates a favorable pathway for membrane permeation of drug molecules. Preprint at bioRxiv. https://doi.org/10.1101/2024.03.18.585530.
54. Lee, T.-H., K. N. Hall, and M.-I. Aguilar. 2016. Antimicrobial Peptide Structure and Mechanism of Action: A Focus on the Role of Membrane Structure. *Curr. Top. Med. Chem.* 16:25–39. http://www.eurekaselect.com/openurl/content.php?genre=article&issn=1568-0266&volume=16&issue=1&spage=25.

Biophysical Journal, Volume *123*

Supplemental information

Optimizing properties of translocation-enhancing transmembrane proteins

Ladislav Bartoš, Martina Drabinová, and Robert Vácha

SUPPORTING MATERIAL

Optimizing properties of translocation-enhancing transmembrane proteins

Ladislav Bartoš, Martina Drabinová, Robert Vácha

Table S1: Distribution of umbrella sampling windows along the collective variable with biasing force constants used for the peptide translocation simulations with POPE:POPG (3:1) membranes. Reference distances are in nm, force constants in kJ mol^{-1} nm^{-2}.

Reference distance	Force constant	Reference distance	Force constant
2.10	3000	0.08	8000
2.00	3000	0.04	8000
1.90	3000	0.00	8000
1.80	3000	−0.04	8000
1.70	3000	−0.08	8000
1.60	3000	−0.12	8000
1.50	3000	−0.16	8000
1.40	3000	−0.20	8000
1.30	3000	−0.24	8000
1.20	3000	−0.28	8000
1.10	3000	−0.32	8000
1.00	3000	−0.36	8000
0.90	3000	−0.40	8000
0.80	3000	−0.43	8000
0.70	3000	−0.46	8000
0.60	3000	−0.50	8000
0.55	5000	−0.55	8000
0.50	8000	−0.60	8000
0.46	8000	−0.65	5000
0.42	8000	−0.70	3000
0.38	8000	−0.80	3000
0.36	8000	−0.90	3000
0.34	8000	−1.00	3000
0.32	8000	−1.10	3000
0.30	8000	−1.20	3000
0.28	8000	−1.30	3000
0.26	8000	−1.40	3000
0.24	8000	−1.50	3000
0.22	8000	−1.60	3000
0.20	8000	−1.70	3000
0.16	8000	−1.80	3000
0.12	8000	−1.90	3000

Table S2: Distribution of umbrella sampling windows along the collective variable with biasing force constants used for the peptide translocation simulations with POPC membranes. Reference distances are in nm, force constants in kJ mol^{-1} nm^{-2}.

Reference distance	Force constant	Reference distance	Force constant
2.10	1000	0.15	5000
2.00	1000	0.10	5000
1.90	1000	0.05	5000
1.80	1000	0.00	5000
1.70	1000	−0.05	5000
1.60	1000	−0.10	5000
1.50	1000	−0.15	5000
1.40	1000	−0.20	5000
1.35	3000	−0.25	5000
1.30	3000	−0.30	5000
1.25	3000	−0.35	5000
1.20	3000	−0.40	5000
1.15	3000	−0.45	5000
1.10	3000	−0.50	3000
1.05	3000	−0.55	3000
1.00	3000	−0.60	3000
0.95	3000	−0.65	3000
0.90	3000	−0.70	3000
0.85	3000	−0.75	3000
0.80	3000	−0.80	3000
0.75	3000	−0.85	3000
0.70	3000	−0.90	3000
0.65	3000	−0.95	3000
0.60	3000	−1.00	1000
0.55	3000	−1.10	1000
0.50	3000	−1.20	1000
0.45	5000	−1.30	1000
0.40	5000	−1.40	1000
0.35	5000	−1.50	1000
0.30	5000	−1.60	1000
0.25	5000	−1.70	1000
0.20	5000	−1.80	1000

Table S3: Distribution of umbrella sampling windows along the collective variable with biasing force constants used for Martini 2 lipid scrambling simulations. Reference distances are in nm, force constants in kJ mol^{-1} nm^{-2}.

Reference distance	Force constant	Reference distance	Force constant
2.30	1000	−0.05	4000
2.20	1000	−0.10	4000
2.10	1000	−0.15	3000
2.00	1000	−0.20	3000
1.90	1000	−0.25	3000
1.80	1000	−0.30	3000
1.70	1000	−0.35	2000
1.60	1000	−0.40	2000
1.50	1000	−0.45	2000
1.40	1000	−0.50	2000
1.30	1000	−0.55	2000
1.20	1000	−0.60	2000
1.10	1000	−0.65	2000
1.00	2000	−0.70	2000
0.95	2000	−0.75	2000
0.90	2000	−0.80	2000
0.85	2000	−0.85	2000
0.80	2000	−0.90	2000
0.75	2000	−0.95	2000
0.70	2000	−1.00	2000
0.65	2000	−1.10	1000
0.60	2000	−1.20	1000
0.55	2000	−1.30	1000
0.50	2000	−1.40	1000
0.45	2000	−1.50	1000
0.40	2000	−1.60	1000
0.35	2000	−1.70	1000
0.30	2000	−1.80	1000
0.25	3000	−1.90	1000
0.20	3000	−2.00	1000
0.15	3000	−2.10	1000
0.10	4000	−2.20	1000
0.05	4000	−2.30	1000
0.00	4000		

Table S4: Distribution of umbrella sampling windows along the collective variable with biasing force constants used for atomistic lipid scrambling simulations with pure POPC membrane. Reference distances are in nm, force constants in kJ mol^{-1} nm^{-2}. ^{R} identifies windows in which the Hamiltonian replica exchange was applied.

Reference distance	Force constant	Reference distance	Force constant
2.30	1000	−0.03	2000^{R}
2.20	1000	−0.06	2000^{R}
2.10	1000	−0.09	2000^{R}
2.00	1000	−0.12	2000^{R}
1.90	1000	−0.15	2000^{R}
1.80	1000	−0.18	2000^{R}
1.70	1000	−0.21	2000^{R}
1.60	1000	−0.25	2000^{R}
1.50	1000	−0.30	1000
1.40	1000	−0.40	1000
1.30	1000	−0.50	1000
1.20	1000	−0.60	1000
1.10	1000	−0.70	1000
1.00	1000	−0.80	1000
0.90	1000	−0.90	1000
0.80	1000	−1.00	1000
0.70	1000	−1.10	1000
0.60	1000	−1.20	1000
0.50	1000	−1.30	1000
0.40	1000	−1.40	1000
0.30	1000	−1.50	1000
0.25	2000	−1.60	1000
0.21	2000^{R}	−1.70	1000
0.18	2000^{R}	−1.80	1000
0.15	2000^{R}	−1.90	1000
0.12	2000^{R}	−2.00	1000
0.09	2000^{R}	−2.10	1000
0.06	2000^{R}	−1.20	1000
0.03	2000^{R}	−1.30	1000
0.00	2000^{R}		

Table S5: Distribution of umbrella sampling windows along the collective variable with biasing force constants used for atomistic lipid scrambling simulations with POPC membrane containing ENHTM3. Reference distances are in nm, force constants in kJ mol^{-1} nm^{-2}. ↓ indicates windows with initial configurations from simulations where the lipid translocated from the upper to lower leaflet. ↑ signifies the opposite direction, and ^{R} denotes windows using Hamiltonian replica exchange.

Reference distance	Force constant	Reference distance	Force constant
2.30	1000↓	−0.03	2000↑^{R}
2.25	1000↓	−0.06	2000↓^{R}
2.20	1000↓	−0.09	2000↑^{R}
2.15	1000↓	−0.12	2000↓^{R}
2.10	1000↓	−0.15	2000↑^{R}
2.05	1000↓	−0.18	2000↓^{R}
2.00	1000↓	−0.21	2000↑^{R}
1.95	1000↓	−0.25	2000↓^{R}
1.90	1000↓	−0.30	1000↑
1.85	1000↓	−0.35	1000↑
1.80	1000↓	−0.40	1000↑
1.75	1000↓	−0.45	1000↑
1.70	1000↓	−0.50	1000↑
1.65	1000↓	−0.55	1000↑
1.60	1000↓	−0.60	1000↑
1.55	1000↓	−0.65	1000↑
1.50	1000↓	−0.70	1000↑
1.45	1000↓	−0.75	1000↑
1.40	1000↓	−0.80	1000↑
1.35	1000↓	−0.85	1000↑
1.30	1000↓	−0.90	1000↑
1.25	1000↓	−0.95	1000↑
1.20	1000↓	−1.00	1000↑
1.15	1000↓	−1.05	1000↑
1.10	1000↓	−1.10	1000↑
1.05	1000↓	−1.15	1000↑
1.00	1000↓	−1.20	1000↑
0.95	1000↓	−1.25	1000↑
0.90	1000↓	−1.30	1000↑
0.85	1000↓	−1.35	1000↑
0.80	1000↓	−1.40	1000↑
0.75	1000↓	−1.45	1000↑
0.70	1000↓	−1.50	1000↑
0.65	1000↓	−1.55	1000↑
0.60	1000↓	−1.60	1000↑
0.55	1000↓	−1.65	1000↑
0.50	1000↓	−1.70	1000↑
0.45	1000↓	−1.75	1000↑
0.40	1000↓	−1.80	1000↑
0.35	1000↓	−1.85	1000↑
0.30	1000↓	−1.90	1000↑
0.25	2000↓	−1.95	1000↑
0.21	2000↑^{R}	−2.00	1000↑
0.18	2000↓^{R}	−2.05	1000↑
0.15	2000↑^{R}	−2.10	1000↑
0.12	2000↓^{R}	−2.15	1000↑
0.09	2000↑^{R}	−2.20	1000↑
0.06	2000↓^{R}	−2.25	1000↑
0.03	2000↑^{R}	−2.30	1000↑
0.00	2000↓^{R}		

Type of hydrophilic residues

Table S6: Free energy differences for important points in the translocation profiles of TLP LS9 translocating through a pure membrane or in the presence of MPs containing different hydrophilic residues. Free energy values are shown relative to the TLP in the adsorbed state [kJ mol^{-1}]. The error was estimated to be below 5 kJ mol^{-1} based on the profile asymmetry. Water defect (WD) and tail defect (TD) [arb. u.] observed around the specific MP and the hydrophobicity (H) of the MP are shown in the last three columns of the table.

MP	ΔG_{IC}	ΔG_{TM}	ΔG_{IN}	$\Delta\Delta G_{BM}$	WD	TD	H
none	81	67	110	110	41.5	N/A	N/A
SAGLS	46	20	74	74	47.3	102.6	0.902
SAGLT	48	20	74	74	47.0	102.6	0.967
SAGLQ	43	28	68	69	48.4	102.2	0.863
SAGLN	34	12	58	58	49.7	102.6	0.780

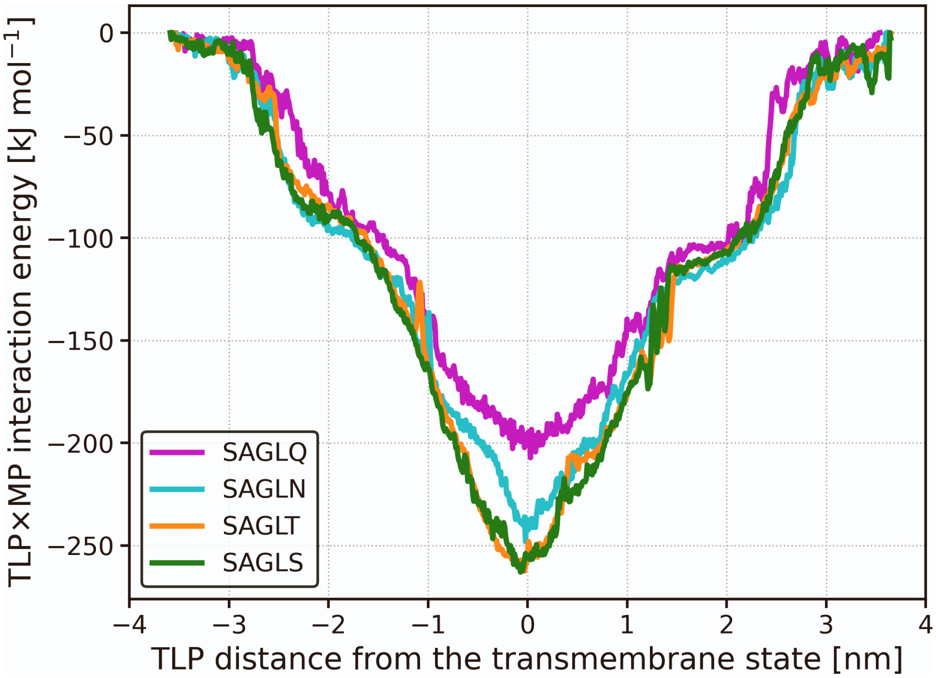

Figure S1: Average strength of the total interaction (Lennard-Jones + coulombic) between the TLP LS9 and MP SAGLQ, SAGLN, SAGLT, or SAGLS calculated from the simulated set of umbrella sampling windows and shown as a function of TLP distance from the transmembrane state. Interaction strength increases as the TLP inserts into the membrane. Note that MP SAGLQ provides significantly weaker enthalpic stabilization for the TLP in the transmembrane state than the other MPs.

Membrane disruption

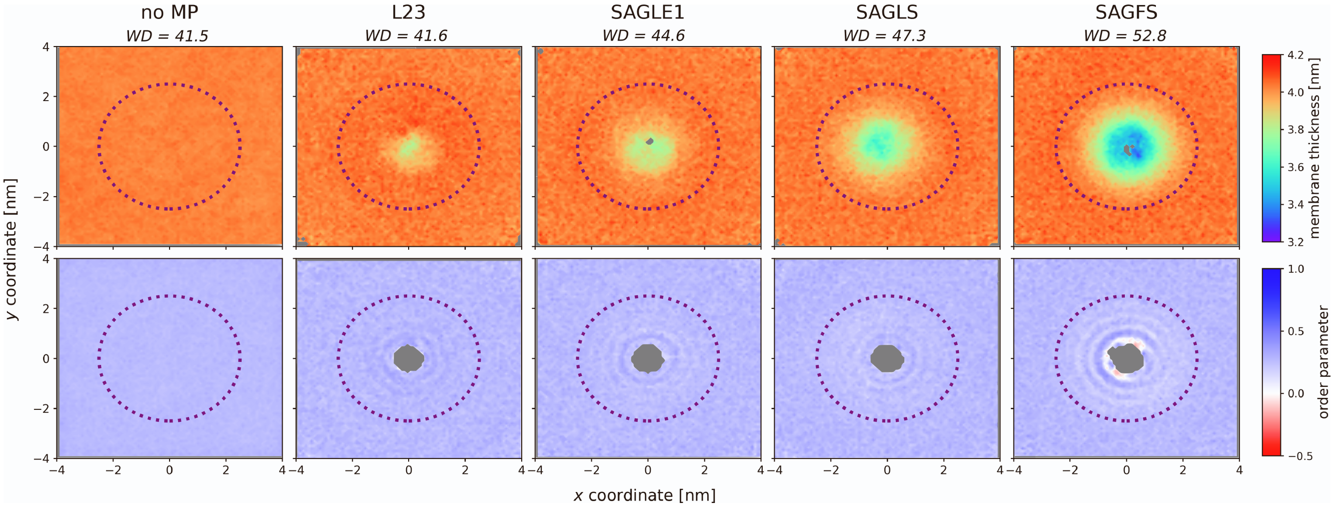

Figure S2: Average membrane thickness (upper row) and coarse-grained order parameters for the C3B-C4B bond of POPE lipids (lower row) calculated for systems both without an MP and with various MPs (L23, SAGLE1, SAGLS, and SAGFS). The water defect calculated for each system is indicated above each chart, beneath the MP's name. The purple circle in each chart highlights the area where the water defect was calculated. Areas in gray indicate insufficient data for calculating the property of interest. A clear correlation is visible between membrane thinning around the MP and the water defect. Disorder in the lipid acyl chains near the MPs, especially notable for the phenylalanine-containing SAGFS peptide, is also visible, though it is generally minor. The membrane thickness maps were generated using the `memthick` tool, available at github.com/Ladme/memdian, and the maps of coarse-grained order parameters using the `ordermap` tool, available at github.com/Ladme/order.

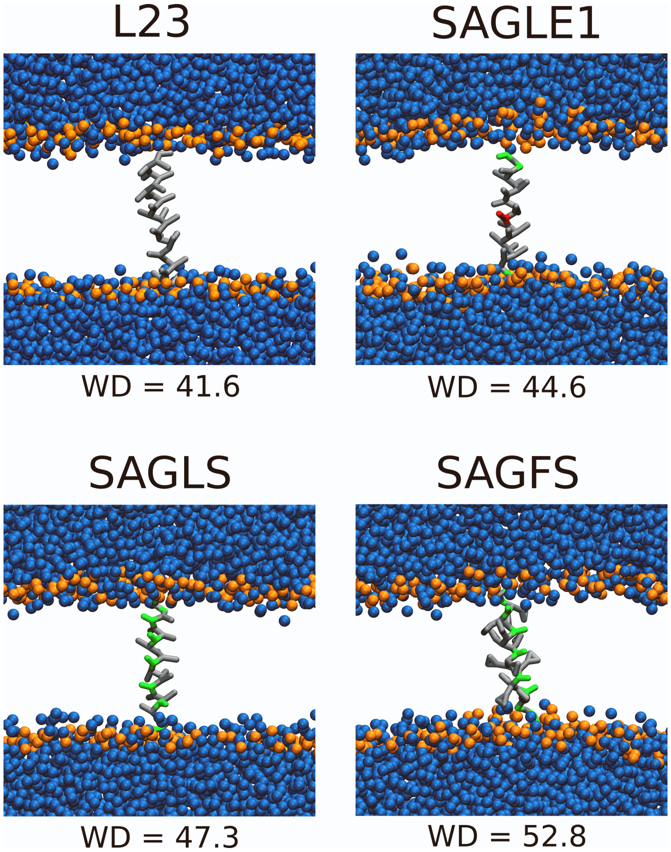

Figure S3: Representative snapshots of MPs L23, SAGLE1, SAGLS, and SAGFS simulated in POPE:POPG (3:1) membranes without the presence of TLP showing that no water channels or pores develop during the simulations with any MPs. Orange and blue spheres represent lipid phosphates and water, respectively. Lipid tails are omitted for clarity, while TLP and MP's hydrophilic and hydrophobic residues appear in green and gray, respectively. Despite displaying a wide range of water defect values, the differences in membrane disruption caused by these MPs are difficult to visually discern.

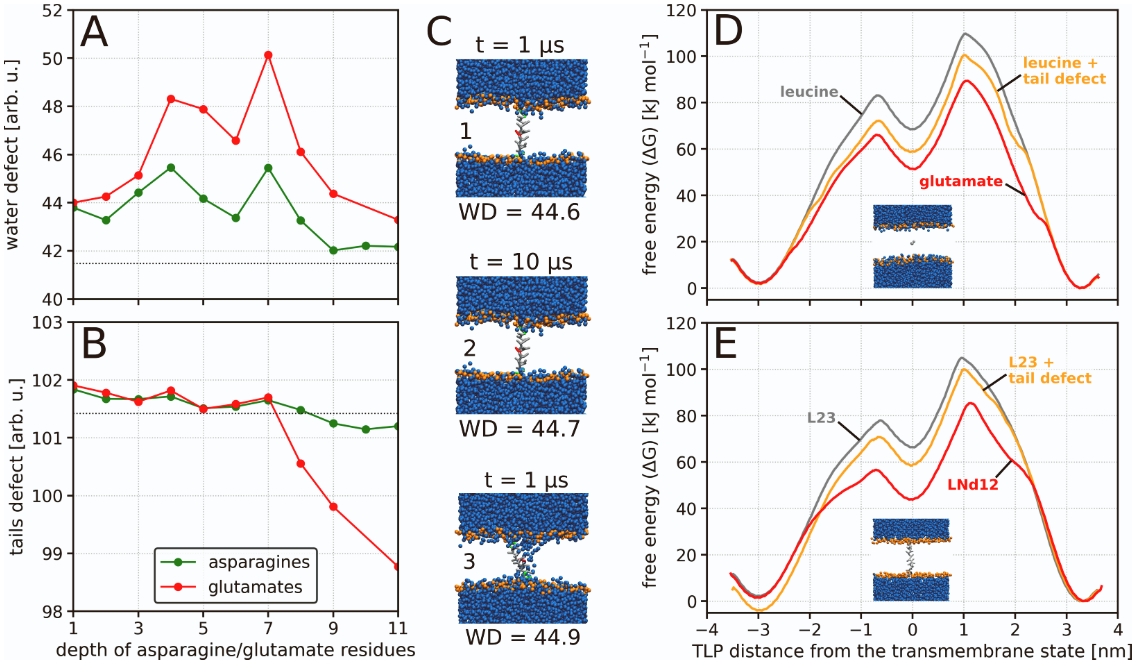

Figure S4: A. Water defects observed around symmetric leucine MPs with two asparagines (green line) or two glutamates (red line) positioned at various sequence depths (different membrane depths). Refer to Table S7 for sequences of these auxiliary MPs. The black dotted line represents the water defect in a pure membrane. The data point for glutamates at depth 10 is absent, as this specific MP (LE2d10) was unstable in the transmembrane state during simulation. B. Tail defects observed for the same MPs as in A. The black dotted line indicates the reference tail defect around MP L23, composed solely of leucines. Note that while MPs with "shallow" or "intermediate" depths of asparagines/glutamates exhibit higher tail defects than L23, those with centrally located asparagines/glutamates lead to a notable reduction in lipid tail density in the membrane center. C. To ensure the reduced water defect seen around MPs with centrally located hydrophilic/charged residues is not a result of initial conditions, we analyzed three simulations of MP SAGLE1 in a POPE:POPG (3:1) membrane: a standard 1 μs simulation (1), an extended 10 μs simulation (2), and a simulation where an artificial pore was introduced at the start of a 1 μs unbiased run (3). All simulations showed similar water defects (44.6 – 44.9 arb. u.). Note that the snapshots show *initial* configurations of the three simulated systems. In system 3, the artificially created pore quickly disappears and the system adopts configurations indistinguishable from systems 1 and 2. The initial pore in system 3 was created using an inverted cylindrical flat-bottomed potential applied to all lipid beads, centered on the membrane's center with a 1.4 nm reference distance in the xy plane and a 50 kJ mol^{-1} nm^{-2} force constant. D. Translocation profiles of the TLP LS9 in the presence of a single leucine or glutamate residue within the membrane. These amino acids were restrained to the membrane's local center of mass using a harmonic potential with a force constant of 5000 kJ mol^{-1} nm^{-2}. We also created an artificial tail defect near a leucine bead, reflecting the tail defect observed around a glutamate bead. This defect was achieved with an inverted spherical flat-bottomed potential applied to all lipid tail beads, centered on the leucine backbone bead with a 0.69 nm reference distance in xyz and a force constant of 500 kJ mol^{-1} nm^{-2}. While this disturbance did not impact the water defect or membrane thickness, the translocation barrier for TLP LS9 decreased in comparison to systems featuring leucine in a stable membrane. However, glutamate resulted in a more significant barrier reduction, indicating important enthalpic contributions and potentially other unidentified effects. E. Translocation profiles of the TLP LS9 with MP L23 or LNd12. We also created an artificial tail defect around L23, reflecting the tail defect observed around LNd12. The defect creation mirrored that in D, but with a 0.66 nm reference distance. There was a noticeable reduction in free energy caused by the artificial tail defect, yet this decrease was smaller than that caused by the charged MP LNd12. It is worth noting that replicating the tail defect around MPs is challenging, as the defect is primarily on one side of the MP, which our defect generation method does not account for.

Table S7: Names and sequences of auxiliary MPs for analysis of relationships between residue depth and water defect and between residue depth and tail defect. The number in the d* suffix denotes the residue depth as depicted in Figure S4 A, C. Although the data for LE2d10 were not utilized due to its instability in the transmembrane state, its sequence is provided for completeness.

Name	Sequence	Name	Sequence
LN2d1	NLLLLLLLLLLLLLLLLLLLLLN	LE2d1	ELLLLLLLLLLLLLLLLLLLLLE
LN2d2	LNLLLLLLLLLLLLLLLLLLLNL	LE2d2	LELLLLLLLLLLLLLLLLLLLEL
LN2d3	LLNLLLLLLLLLLLLLLLLLNLL	LE2d3	LLELLLLLLLLLLLLLLLLLELL
LN2d4	LLLNLLLLLLLLLLLLLLLNLLL	LE2d4	LLLELLLLLLLLLLLLLLLELLL
LN2d5	LLLLNLLLLLLLLLLLLLNLLLL	LE2d5	LLLLELLLLLLLLLLLLLELLLL
LN2d6	LLLLLNLLLLLLLLLLLNLLLLL	LE2d6	LLLLLELLLLLLLLLLLELLLLL
LN2d7	LLLLLLNLLLLLLLLLNLLLLLL	LE2d7	LLLLLLELLLLLLLLLELLLLLL
LN2d8	LLLLLLLNLLLLLLLNLLLLLLL	LE2d8	LLLLLLLELLLLLLLELLLLLLL
LN2d9	LLLLLLLLNLLLLLNLLLLLLLL	LE2d9	LLLLLLLLELLLLLELLLLLLLL
LN2d10	LLLLLLLLLNLLLNLLLLLLLLL	LE2d10	LLLLLLLLLELLLELLLLLLLLL
LN2d11	LLLLLLLLLLNLNLLLLLLLLLL	LE2d11	LLLLLLLLLLELELLLLLLLLLL

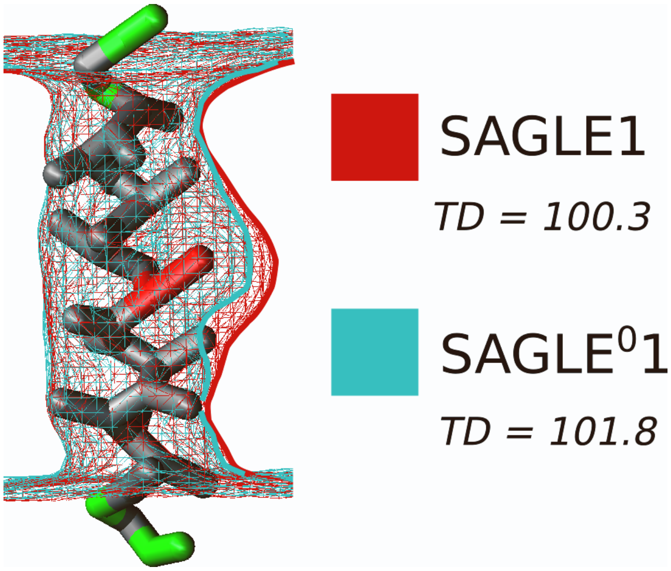

Figure S5: Isosurface representation of lipid tail densities calculated around the MPs SAGLE1 (dark red) and SAGLE^{0}1 (teal). The peptides differ only in the charge of the central glutamate residue, with SAGLE1's glutamate being charged and SAGLE^{0}1's being uncharged. As a result, the lipid density near SAGLE1 is lower ("tail defect" of 100.3 arb. u.) compared to that near SAGLE^{0}1 ("tail defect" of 101.8 arb. u.). This decrease in lipid tail density near SAGLE1 manifests as a pronounced protrusion in the density isosurface at the membrane's center. This density change is minor and not visible in individual simulation snapshots, but still affects peptide translocation.

Depth of hydrophilic residues

Table S8: Free energy differences for important points in the translocation profiles of TLP LS9 translocating in the presence of MPs with different depths of hydrophilic residues. Both translocation directions are shown separately. Free energy values are shown relative to the TLP in the adsorbed state [kJ mol^{-1}]. The error was estimated to be below 5 kJ mol^{-1}. Water defect (WD), upper leaflet water defect (uWD), lower leaflet water defect (lWD), tail defect (TD), upper leaflet tail defect (uTD), and lower leaflet tail defect (lTD), all in arb. u., are shown in the last six columns.

MP	direction	ΔG_{IC}	ΔG_{TM}	ΔG_{IN}	$\Delta\Delta G_{\mathrm{BM}}$	WD	uWD	lWD	TD	uTD	lTD
LNd3	N↓C↑	73	58	98	98	44.0	22.2	21.8	101.6	51.0	50.6
	N↑C↓	69	56	96	97						
LNd6	N↓C↑	68	50	91	91	46.0	23.2	22.8	101.6	50.9	50.7
	N↑C↓	68	55	92	92						
LNd9	N↓C↑	59	42	84	84	44.6	22.7	21.9	101.4	50.8	50.6
	N↑C↓	59	46	83	83						
LNd12	both	57	44	85	85	42.2	21.9	20.3	101.2	50.7	50.5

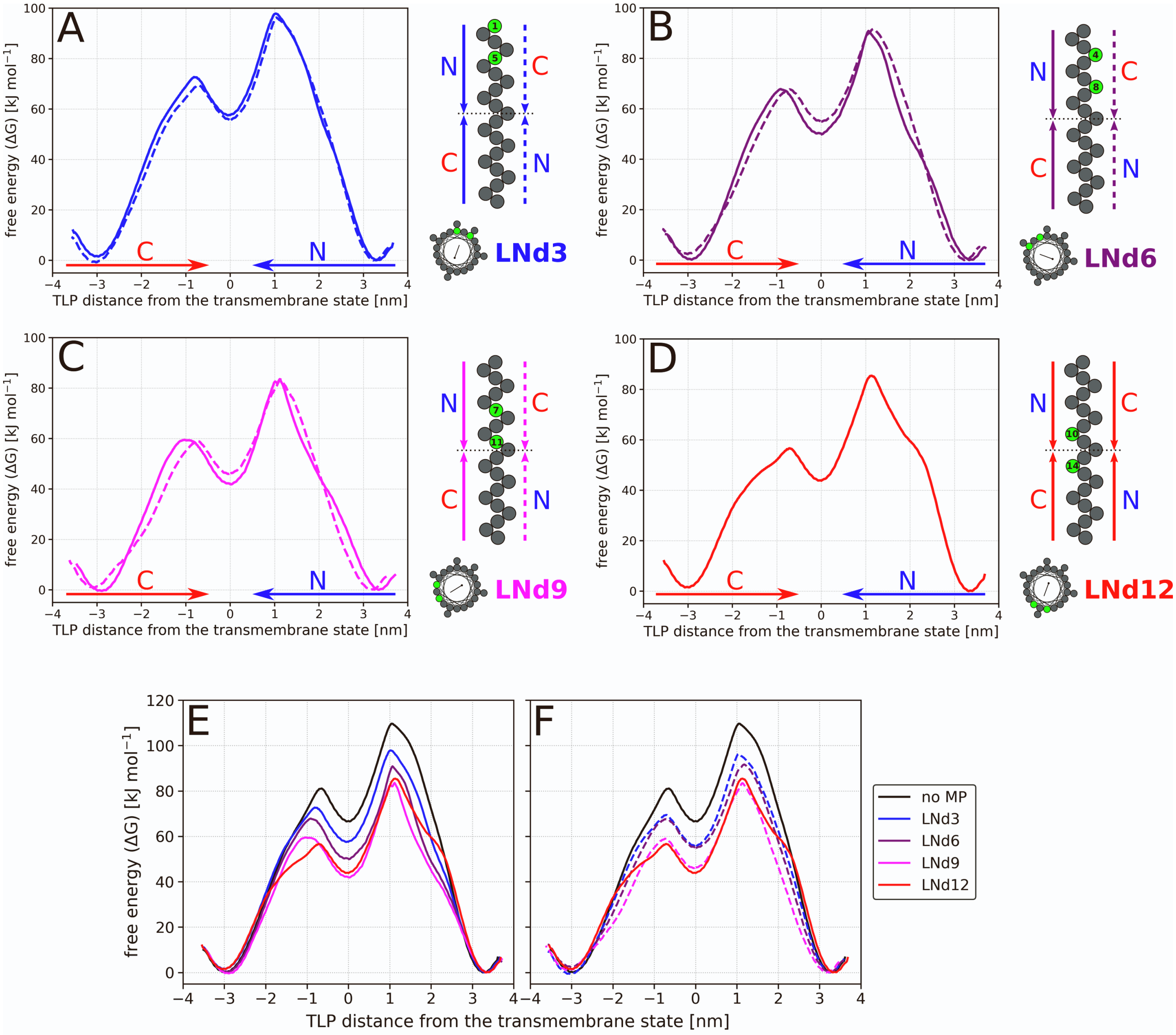

Figure S6: A—D. Translocation profiles of the TLP LS9 in the presence of MP LNd3 (A), LNd6 (B), LNd9 (C), and LNd12 (D). For asymmetric MPs (A–C), both translocation directions are presented. The N↓C↑ direction is represented with solid lines, and the N↑C↓ direction with dashed lines. The negative portion of each profile indicates C-terminus insertion, and the positive indicates N-terminus insertion. A schematic representation of the respective MP, highlighting the depth of its hydrophilic residues and the translocation directions, is positioned to the right of each chart. The MP's name and its helical wheel are displayed beneath the schematic. It is noteworthy that with asymmetric MPs (LNd3, LNd6, LNd9), the free energy profiles of the TLP for both translocation directions appear quite similar, potentially due to the apparent symmetrization of membrane disruption when these MPs are present. E—F. Translocation profiles of TLP LS9 traversing the membrane alone (in black) or along MP LNd3 (in blue), LNd6 (in purple), LNd9 (in pink), or LNd12 (in red). E outlines the translocation profiles for the N↓C↑ direction, while F depicts the N↑C↓ translocation direction. A deeper average position of the asparagines results in enhanced translocation.

Hydrophilic patch compactness

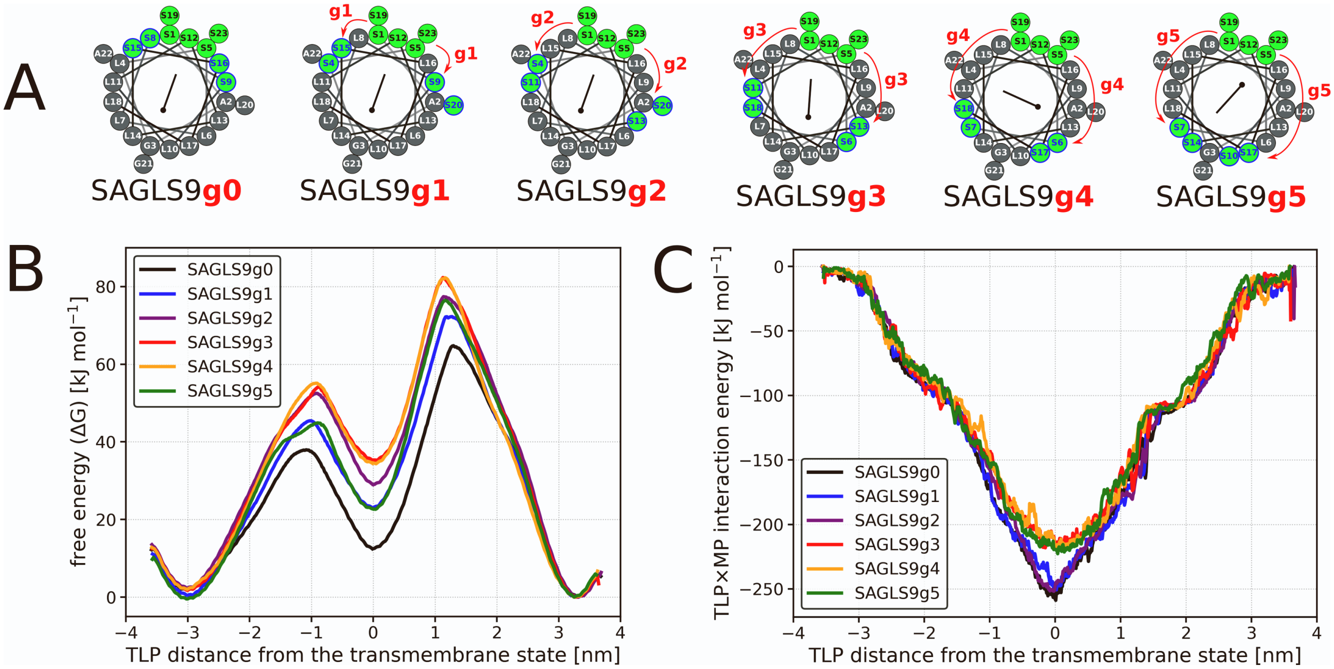

Figure S7: A. Helical wheel diagrams of the MPs SAGLS9g0–5. Each MP features a hydrophilic patch composed of serines 1, 5, 12, 19, and 23, with additional four serines (marked in blue) which positions vary. The g* suffix designates the number of hydrophobic residues in the helical wheel that separate the primary hydrophilic patch from the supplementary serines, termed a "gap". B. The translocation profiles of TLP LS9 as it moves through the membrane in the presence of MPs that have a disrupted hydrophilic patch, SAGLS9g0–5. Disrupting the hydrophilic patch of the MP notably diminishes the MP's ability to enhance translocation. C. The average strength of the total interaction (Lennard-Jones + coulombic) between TLP LS9 and MP SAGLS9g0–5. The interaction strength is computed from the simulated set of umbrella sampling windows and is represented as a function of the TLP's distance from its transmembrane state. It is worth noting that MPs with considerable disruptions to their hydrophilic patches (such as SAGLS9g3–5) offer significantly reduced enthalpic stabilization for the TLP when in a transmembrane state. This is due to the diminished count of hydrophilic residues in optimal positions that can interact with the TLP.

Table S9: Free energy differences for important points in the translocation profiles of TLP LS9 translocating in the presence of MPs with various hydrophilic patch compactness. Free energy values are shown relative to the TLP in the adsorbed state [kJ mol^{-1}]. The error was estimated to be below 5 kJ mol^{-1}. Water defect (WD) and tail defect (TD) [arb. u.] are shown in the last two columns of the table.

MP	ΔG_{IC}	ΔG_{TM}	ΔG_{IN}	$\Delta\Delta G_{BM}$	WD	TD
SAGLS9g0	38	12	65	65	48.5	102.8
SAGLS9g1	45	23	72	72	48.4	103.0
SAGLS9g2	53	29	77	77	47.8	102.9
SAGLS9g3	54	35	82	82	48.5	102.9
SAGLS9g4	55	35	82	82	49.2	103.1
SAGLS9g5	45	23	77	77	47.9	102.8

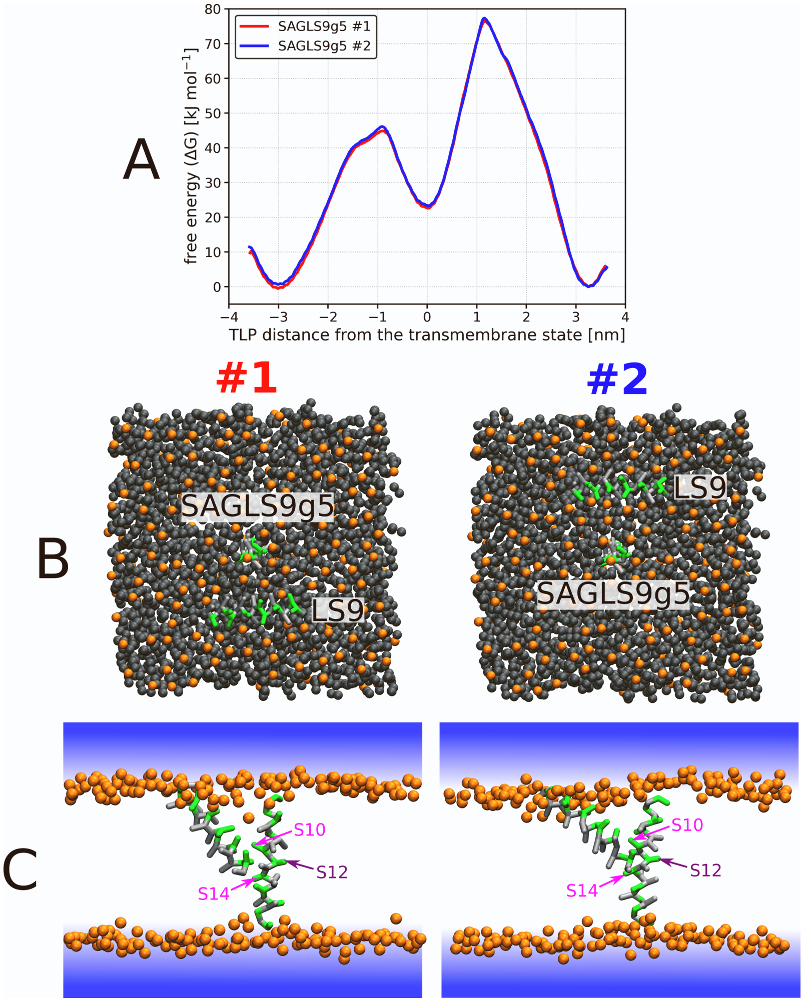

Figure S8: The ability of MP SAGLS9g5 to enhance translocation is not as diminished as we anticipated, considering the disruption of its hydrophilic patch. This phenomenon can be attributed to the four supplementary serines with varied positions that come very close to one another and generally point in the same direction (as illustrated in Figure S7 A). These additional serines create a secondary hydrophilic patch on the opposite side of the primary patch. Given that these specific serines (S7, S10, S14, and S17) are, on average, more centrally positioned within the membrane than those of the primary patch (S1, S5, S12, S19, S23), they offer greater stabilization for the TLP. As a result, the TLP tends to translocate along this secondary hydrophilic patch. To ensure that the TLP's preference for the secondary patch over the primary one is not merely an artifact of initial conditions, we performed two simulations of the TLP with SAGLS9g5, each with a different initial position of the TLP in its adsorbed state (B). The translocation profiles calculated from these two systems with differing starting conditions are nearly indistinguishable (A). In both simulations, the TLP selects the same translocation pathway (C). For all subsequent analyses, we have chosen to use the free energy profile from system #1 as the standard translocation profile for TLP LS9 when in the presence of SAGLS9g5.

Presence of charged residues

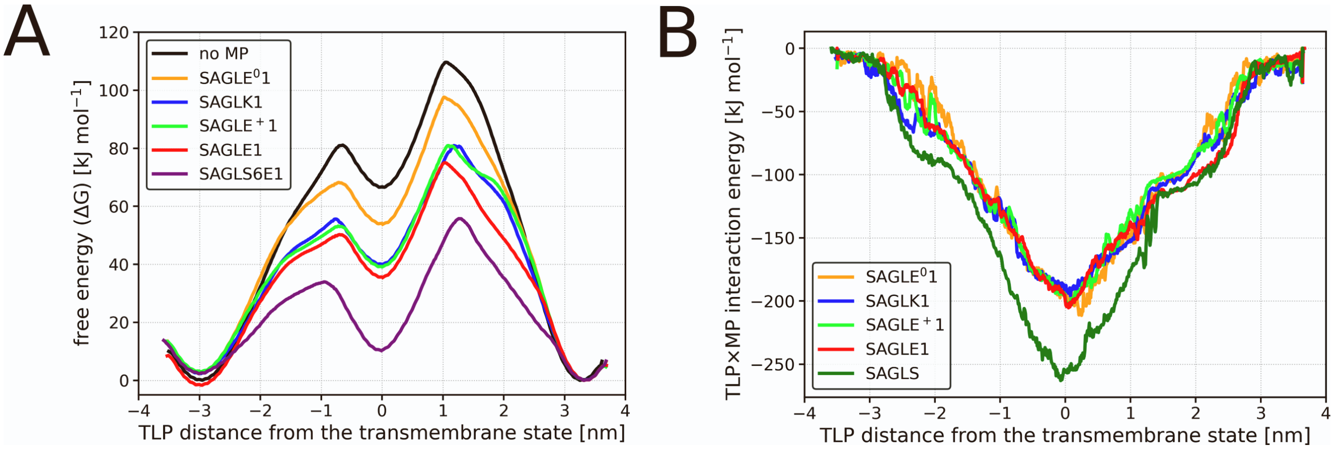

Figure S9: A. Translocation profiles of the TLP LS9 moving through the membrane alone or in the presence of MP SAGLE1, SAGLK1, SAGLE^{0}1, SAGLE$^+$1, or SAGLS6E1. Even a single charged residue in the MP reduces the translocation barrier, but its effect on the TLP's stability in the transmembrane state is less pronounced. Incorporating a charged residue into an MP with a hydrophilic patch can also enhance translocation. B. Average strength of the total interaction (Lennard-Jones + coulombic) between the TLP LS9 and the MP SAGLK1, SAGLE^{0}1, SAGLE$^+$1, SAGLE1, or SAGLS, calculated from simulated umbrella sampling windows and represented as a function of the TLP's distance from its transmembrane state. Note that the interaction between the TLP and the MP SAGLS is notably stronger than interaction between the TLP and the other MPs, especially in the transmembrane state of the TLP. This results in decreased stability of the TLP in its transmembrane state during translocation along MPs like SAGLK1, SAGLE$^+$1, SAGLE^{0}1, or SAGLE1, compared to the MP SAGLS.

Table S10: Free energy differences for important points in the translocation profiles of TLP LS9 translocating in the presence of MPs with charged residues or with a protonated glutamate. Free energy values are shown relative to the TLP in the adsorbed state [kJ mol^{-1}]. The error was estimated to be below 5 kJ mol^{-1}. Water defect (WD) and tail defect (TD) [arb. u.] are shown in the last two columns of the table.

MP	ΔG_{IC}	ΔG_{TM}	ΔG_{IN}	$\Delta\Delta G_{\mathrm{BM}}$	WD	TD
SAGLE1	50	35	75	77	44.6	100.3
SAGLE^{0}1	68	54	98	98	44.4	101.8
SAGLE$^+$1	53	39	81	81	44.9	100.3
SAGLK1	55	40	81	81	45.3	100.8
SAGLS6E1	34	10	56	56	47.8	101.3

Type of hydrophobic residues

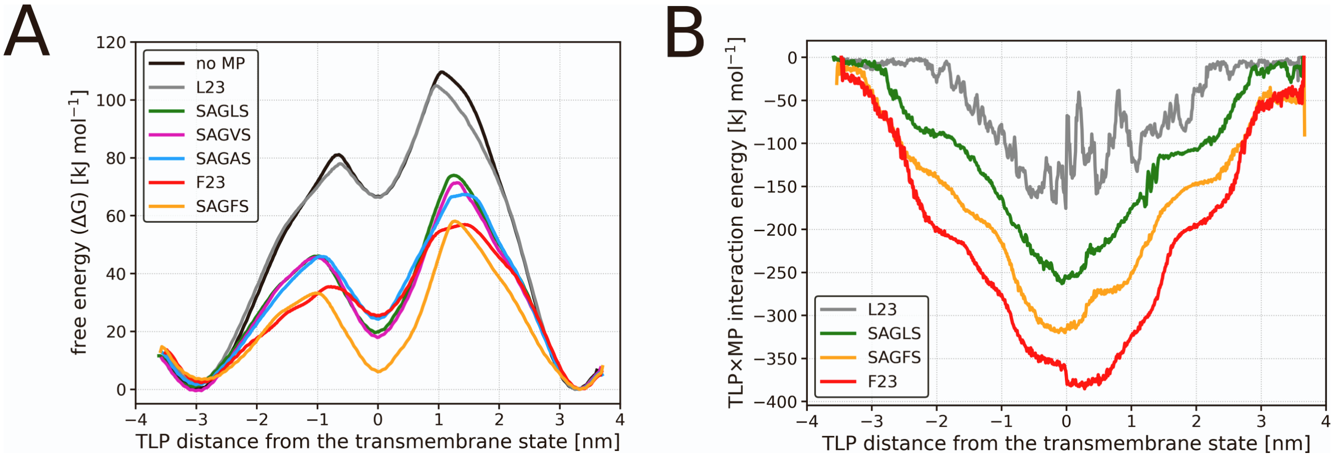

Figure S10: A. Translocation profiles of the TLP LS9 moving through the membrane alone or in the presence of MPs L23, SAGLS, SAGVS, SAGAS, F23, or SAGFS. The character of the hydrophobic residues composing the MP can in many cases significantly affect the translocation-enhancing abilities of the MP. B. Average strength of the total interaction (Lennard-Jones + coulombic) between the TLP LS9 and MP L23, SAGLS, SAGFS, or F23 calculated from the simulated set of umbrella sampling windows and shown as a function of the TLP distance from the transmembrane state. Observe the difference in enthalpic stabilization between MPs L23 and F23, as well as between SAGLS and SAGFS.

Table S11: Free energy differences for important points in the translocation profiles of TLP LS9 translocating in the presence of MPs containing different hydrophobic residues. Free energy values are shown relative to the TLP in the adsorbed state [kJ mol^{-1}]. The error was estimated to be below 5 kJ mol^{-1}. Water defect (WD) and tail defect (TD) [arb. u.] are shown in the last two columns of the table. Note that the values of the tail defect are heavily biased for this set of MPs due to the different sizes of the sidechains of the hydrophobic residues.

MP	ΔG_{IC}	ΔG_{TM}	ΔG_{IN}	$\Delta\Delta G_{\mathrm{BM}}$	WD	TD
SAGLS	46	20	74	74	47.3	102.6
SAGVS	46	18	71	72	47.2	103.3
SAGAS	41	18	66	67	47.3	106.5
SAGFS	33	6	58	58	52.8	100.0
F23	35	25	57	57	45.9	97.3
L23	78	66	105	105	41.6	101.4

Relationship between $\Delta\Delta G_{\mathbf{BM}}$ and $\Delta G_{\mathbf{TM}}$

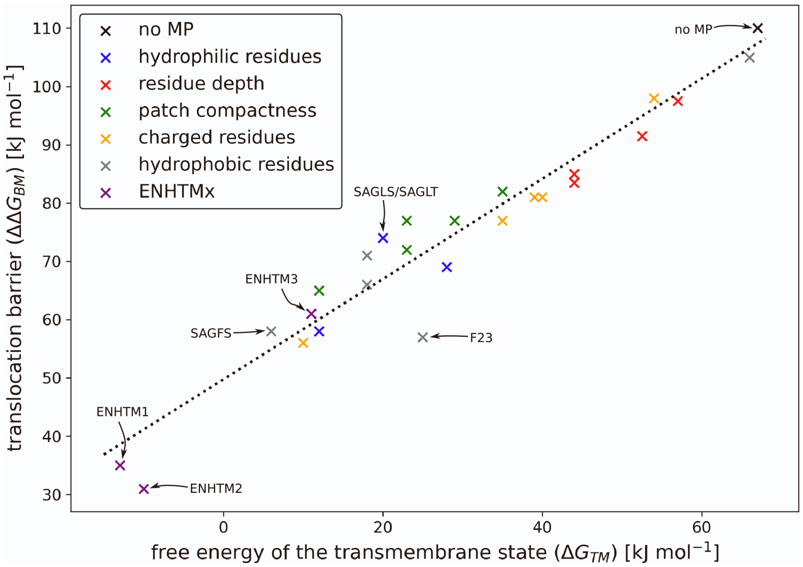

Figure S11: The relationship between the translocation barrier, $\Delta\Delta G_{\mathrm{BM}}$, and the free energy of the transmembrane state, ΔG_{TM}, calculated for all simulated systems. Individual points represent the systems, with colors indicating the “type” of MP present. Several MPs of interest are labeled. The relationship between these properties is largely linear, as evidenced by a correlation coefficient of 0.96.

Translocation across POPC membranes

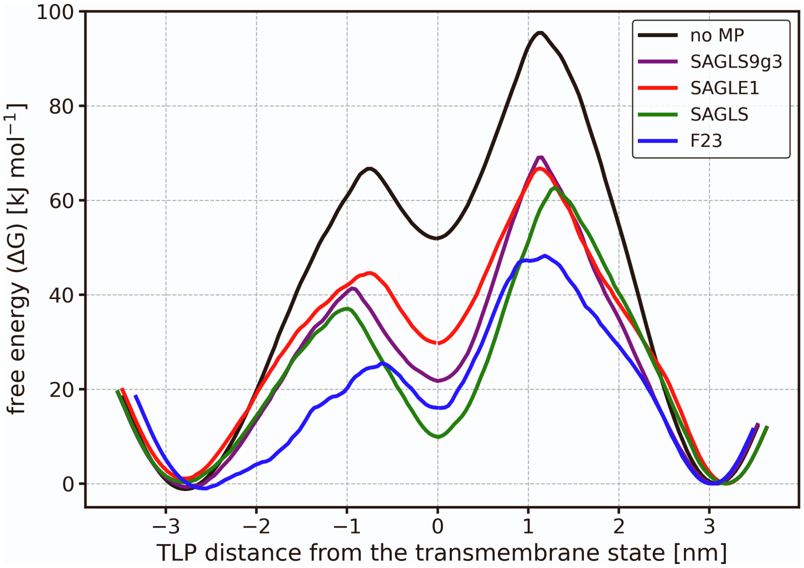

Figure S12: Translocation profiles of the TLP LS9 moving through a POPC membrane alone or in the presence of various MPs (SAGLS9g3, SAGLE1, SAGLS, F23). The translocation barriers across the POPC membrane are generally lower than those across the POPE:POPG (3:1) membrane, but MPs continue to exhibit their translocation-enhancing abilities.

Table S12: Free energy differences for important points in the translocation profiles of TLP LS9 translocating through pure POPC membrane or through POPC membrane in the presence of various MPs. Free energy values are shown relative to the TLP in the adsorbed state [kJ mol^{-1}]. The error was estimated to be below 5 kJ mol^{-1}. Water defect (WD) and tail defect (TD) [arb. u.] are shown in the last two columns of the table.

MP	ΔG_{IC}	ΔG_{TM}	ΔG_{IN}	$\Delta\Delta G_{\mathrm{BM}}$	WD	TD
no MP	67	52	95	97	64.0	N/A
SAGLS9g3	41	22	69	70	72.0	102.9
SAGLE1	45	30	67	67	66.0	100.3
SAGLS	37	10	63	63	69.6	102.6
F23	25	16	48	49	66.4	97.1
